# Comprehensive Sieve Analysis of Breakthrough HIV-1 Sequences in the RV144 Vaccine Efficacy Trial

**DOI:** 10.1371/journal.pcbi.1003973

**Published:** 2015-02-03

**Authors:** Paul T. Edlefsen, Morgane Rolland, Tomer Hertz, Sodsai Tovanabutra, Andrew J. Gartland, Allan C. deCamp, Craig A. Magaret, Hasan Ahmed, Raphael Gottardo, Michal Juraska, Connor McCoy, Brendan B. Larsen, Eric Sanders-Buell, Chris Carrico, Sergey Menis, Meera Bose, RV144 Sequencing Team, Miguel A. Arroyo, Robert J. O’Connell, Sorachai Nitayaphan, Punnee Pitisuttithum, Jaranit Kaewkungwal, Supachai Rerks-Ngarm, Merlin L. Robb, Tatsiana Kirys, Ivelin S. Georgiev, Peter D. Kwong, Konrad Scheffler, Sergei L. Kosakovsky Pond, Jonathan M. Carlson, Nelson L. Michael, William R. Schief, James I. Mullins, Jerome H. Kim, Peter B. Gilbert

**Affiliations:** 1 Vaccine and Infectious Disease Division, Fred Hutchinson Cancer Research Center, Seattle, Washington, United States of America; 2 US Military HIV Research Program, Silver Spring, Maryland, United States of America; 3 The Shraga Segal Dept. of Microbiology, Immunology and Genetics, Faculty of Health Sciences, and The National Institute for Biotechnology in the Negev, Ben-Gurion University of the Negev, Beer-Sheva, Israel; 4 Department of Microbiology, University of Washington, Seattle, Washington, United States of America; 5 Department of Biochemistry, University of Washington, Seattle, Washington, United States of America; 6 IAVI Neutralizing Antibody Center and Department of Immunology and Microbial Sciences, The Scripps Research Institute, La Jolla, California, United States of America; 7 Royal Thai Army Component, AFRIMS, Bangkok, Thailand; 8 US Army Component, AFRIMS, Bangkok, Thailand; 9 Faculty of Tropical Medicine, Mahidol University, Bangkok, Thailand; 10 CDC Department, Thai Ministry of Public Health, Nonthaburi, Thailand; 11 Vaccine Research Center, NIAID, NIH, Bethesda, Maryland, United States of America; 12 Department of Medicine, University of California, San Diego, La Jolla, California, United States of America; 13 eSience Research Group, Microsoft Research, Redmond, Washington, United States of America; 14 Ragon Institute of MGH, MIT and Harvard, Cambridge, Massachusetts, United States of America; La Jolla Institute for Allergy and Immunology, UNITED STATES

## Abstract

The RV144 clinical trial showed the partial efficacy of a vaccine regimen with an estimated vaccine efficacy (VE) of 31% for protecting low-risk Thai volunteers against acquisition of HIV-1. The impact of vaccine-induced immune responses can be investigated through sieve analysis of HIV-1 breakthrough infections (infected vaccine and placebo recipients). A V1/V2-targeted comparison of the genomes of HIV-1 breakthrough viruses identified two V2 amino acid sites that differed between the vaccine and placebo groups. Here we extended the V1/V2 analysis to the entire HIV-1 genome using an array of methods based on individual sites, k-mers and genes/proteins. We identified 56 amino acid sites or “signatures” and 119 k-mers that differed between the vaccine and placebo groups. Of those, 19 sites and 38 k-mers were located in the regions comprising the RV144 vaccine (Env-gp120, Gag, and Pro). The nine signature sites in Env-gp120 were significantly enriched for known antibody-associated sites (p = 0.0021). In particular, site 317 in the third variable loop (V3) overlapped with a hotspot of antibody recognition, and sites 369 and 424 were linked to CD4 binding site neutralization. The identified signature sites significantly covaried with other sites across the genome (mean = 32.1) more than did non-signature sites (mean = 0.9) (p < 0.0001), suggesting functional and/or structural relevance of the signature sites. Since signature sites were not preferentially restricted to the vaccine immunogens and because most of the associations were insignificant following correction for multiple testing, we predict that few of the genetic differences are strongly linked to the RV144 vaccine-induced immune pressure. In addition to presenting results of the first complete-genome analysis of the breakthrough infections in the RV144 trial, this work describes a set of statistical methods and tools applicable to analysis of breakthrough infection genomes in general vaccine efficacy trials for diverse pathogens.

## Author Summary

We present an analysis of the genomes of the HIV viruses that infected some participants of the RV144 Thai trial, which was the first study to show efficacy of a vaccine to prevent HIV infection. We analyzed the HIV genomes of infected vaccine recipients and infected placebo recipients, and found differences between them. These differences coincide with previously-studied genetic features that are relevant to the biology of HIV infection, including features involved in immune recognition of the virus. The findings presented here generate testable hypotheses about the mechanism of the partial protection seen in the Thai trial, and may ultimately lead to improved vaccines. The article also presents a toolkit of methods for computational analyses that can be applied to other vaccine efficacy trials.

## Introduction

The HIV pandemic is responsible for more than 34 million deaths worldwide. Analysis of the RV144 vaccine trial yielded an estimated efficacy to prevent HIV infection of 31%, with a 95% confidence interval (CI) of 1% to 51% [1]. In this phase III efficacy trial, 16,402 Thai HIV-1-negative volunteers were randomized to receive a prime-boost vaccine regimen that consisted of four priming injections of a recombinant canarypox vector [ALVAC-HIV vCP1521: subtype B *gag, pro* (from HIV-1 strain LAI) and CRF01_AE gp120 (92TH023)], and two booster injections of a recombinant gp120 subunit vaccine [AIDSVAX B/E: subtype B (MN) and CRF01_AE (CM244)]. Follow-up studies highlighted possible mechanisms behind the modest RV144 protection. Multiple sources of evidence indicated a role for vaccine-induced antibody responses targeting the V2 region of the envelope glycoprotein (Env): (1) the case-control study of immune correlates of risk showed that the magnitude of IgG antibodies binding to the V1/V2 region of Env was inversely correlated with risk of infection [2–5]; (2) the magnitude of binding of IgG antibodies to linear peptides in the V2 loop was inversely correlated with risk of infection [3,6]; and (3) sieve analysis targeted to the V2 region (described below) demonstrated vaccine pressure at two sites [7]. The case-control correlates study also showed that IgA antibodies to envelope and to the C1 region of Env were directly correlated with risk of infection [3]. In addition, among vaccine recipients with low IgA antibody responses to Env, HIV-1 infection risk was inversely correlated with IgG Env antibody avidity, antibody-dependent cellular cytotoxicity, neutralizing antibodies, and Env-specific CD4+ T cell responses [3], as well as with IgG to V3 linear peptides [6].

“Sieve analysis” is the statistical assessment of whether and how the efficacy of a vaccine depends upon characteristics of the pathogen. Genomic sieve analysis compares breakthrough HIV-1 sequences between the infected vaccine and infected placebo groups. A sieve analysis of the HVTN 502/Step trial, with a vaccine inducing cytotoxic T-lymphocyte (CTL) responses, found evidence of CTL epitope-specific variation [8,9]. Based on HIV-1 breakthrough infections in the RV144 trial sequenced at the time of HIV-1 diagnosis, a sieve analysis that focused on the V1/V2 region of Env identified two sites in the V2 loop (HXB2 amino acids Env 169 and 181) at which the level of efficacy of the vaccine significantly differed depending on whether the genome of the infecting HIV-1 virus matched the vaccine immunogen sequence at the site [7].

**Figure 1:**
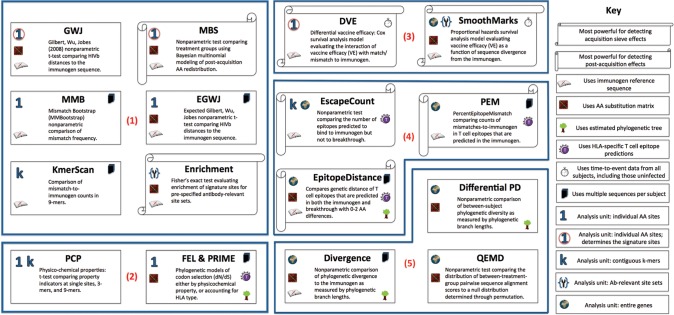
Analysis methods. Methods evaluate (1) differential deviation (vaccine versus placebo) from the immunogen sequences at specific loci or in peptide regions that are relevant to antibody binding; (2) differential codon selection, and differences in physico-chemical properties across vaccine and placebo; (3) differential vaccine efficacy versus HIV-1 sequences that do not match immunogen sequences at individual sites and in each of several pre-specified antibody-relevant protein regions; (4) greater or more rapid viral escape (vaccine versus placebo) at predicted class I and class II HLA-restricted T cell epitopes; and (5) differences in phylogenetic diversity of the breakthrough amino acid sequences (vaccine versus placebo) or differential evolutionary divergence from the vaccine immunogen sequences. T cell and tree images are from openclipart.org.

Here we present a comprehensive genome-wide exploratory sieve analysis of the breakthrough HIV-1 sequences of 109 of the 110 RV144 participants who were infected with CRF01_AE (excluding one subject whose infection was epidemiologically linked and secondary to another study participant’s infection [7]). Our investigation was based on a pre-specified analysis plan, and included multiple sieve analysis methods, each of which evaluates a different immunological hypothesis (Fig. 1). The analysis focused on amino acid (AA) site-, peptide-, and protein-specific methods, with investigation of (1) differential deviation (vaccine versus placebo) from the immunogen sequences at specific loci or in peptide regions that are relevant to antibody binding; (2) differential vaccine efficacy versus HIV-1 sequences that do not match immunogen sequences at individual sites and in each of several pre-specified antibody-relevant protein regions; (3) differential codon selection, and differences in physico-chemical properties across treatment groups; (4) greater or more rapid viral escape (vaccine versus placebo) at predicted class I and class II HLA-restricted T cell epitopes; and (5) differences in phylogenetic diversity of the breakthrough amino acid sequences or differential evolutionary divergence from the vaccine immunogen sequences. The results of these analyses generate testable hypotheses about the mechanisms underlying the modest protection induced by the RV144 vaccine regimen and about potential paths to more effective HIV-1 vaccines to be investigated in future research.

## Results

### Novel methods and discoveries

We applied an array of methods designed to evaluate distinct hypotheses regarding vaccine-induced effects on the genetic sequences of the breakthrough HIV-1 viruses. The methods and their relative merits are outlined in Fig. 1 and in the Materials and Methods section. All analysis methods used here have been described previously except for the “Expected Gilbert, Wu, Jobes” (EGWJ) method, the “Quasi-Earth Mover’s Distance” (QEMD) method, and the “Physico-chemical Properties” method (PCP), which are described here for the first time. The EscapeCount method was also developed for this analysis; it has been reported previously [10], but is described more thoroughly here. This paper is the also first published application of the PRIME method (http://hyphy.org/w/index.php/PRIME).

All results reported here have not been reported previously except for the V2 crown signature sites (Env 169 and Env 181) [7], and the V3 signature site (Env 317) [11]. In addition, one epitope region found by the EscapeCount method (at the crown of the V2 loop) was reported previously [10]. The SmoothMarks method has been applied to evaluate a related genetic distance, but as described below the results shown here are novel.

### Amino acid and peptide signatures in Env-gp120, Pro and Gag and in non-vaccine proteins

**Figure 2:**
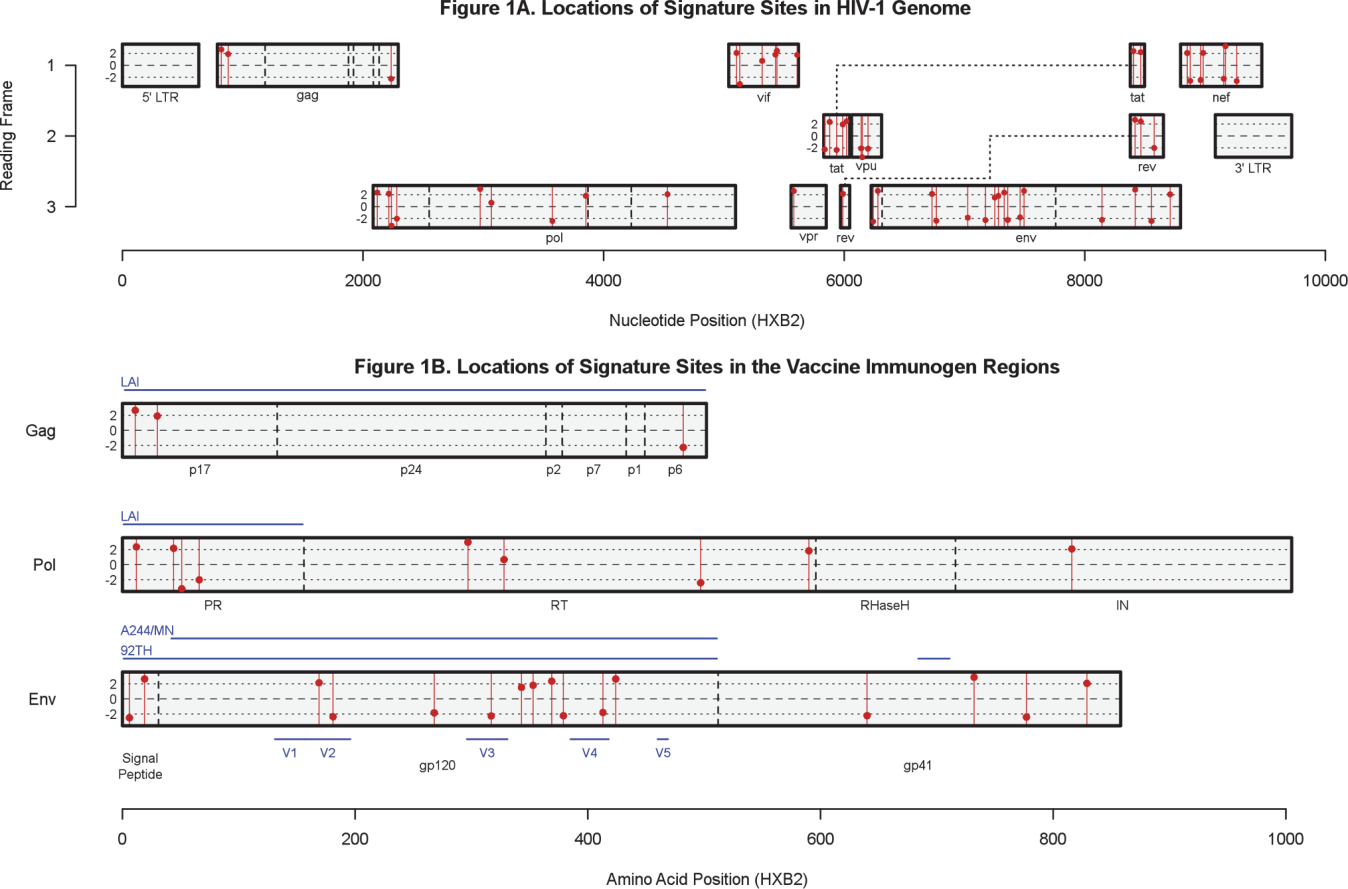
Signature sites by HIV-1 protein. All sites with evidence for a different amino acid distribution in vaccine versus placebo sequences relative to a reference residue (unadjusted p < 0.05) by any of the site-scanning methods DVE, GWJ, or MBS. Panel (A) depicts signature sites found in the full HIV-1 genome, including in vaccine-immunogen regions (relative to a vaccine immunogen sequence) and non-vaccine-immunogen regions (relative to the consensus AE sequence) and (B) depicts a more detailed view of the vaccine immunogen regions in Gag, Pol, and Env. The blue horizontal lines above the protein regions in panel (B) indicate the regions included in the vaccine immunogen sequences, and the blue lines below the Env protein region indicate the positions of the variable loops. Signature sites are indicated by red vertical lines, with a red point that is placed on the line as an indicator of the magnitude of the site’s test statistic using the GWJ method, which is a t statistic comparing substitution weights across treatment groups. For sites with multiple reference AAs, the red point indicates the largest magnitude of the multiple test statistics. The black dashed horizontal lines in the middle of the gene and protein regions indicate the zero-point for the test statistic, so the farther away the point is from the center line, the more significant it was observed to be with this method. Points above the dashed line indicate that a site was found to have a “vMatch” sieve effect, while points below the dashed line indicate “vMismatch” signature sites.

**Figure 3:**
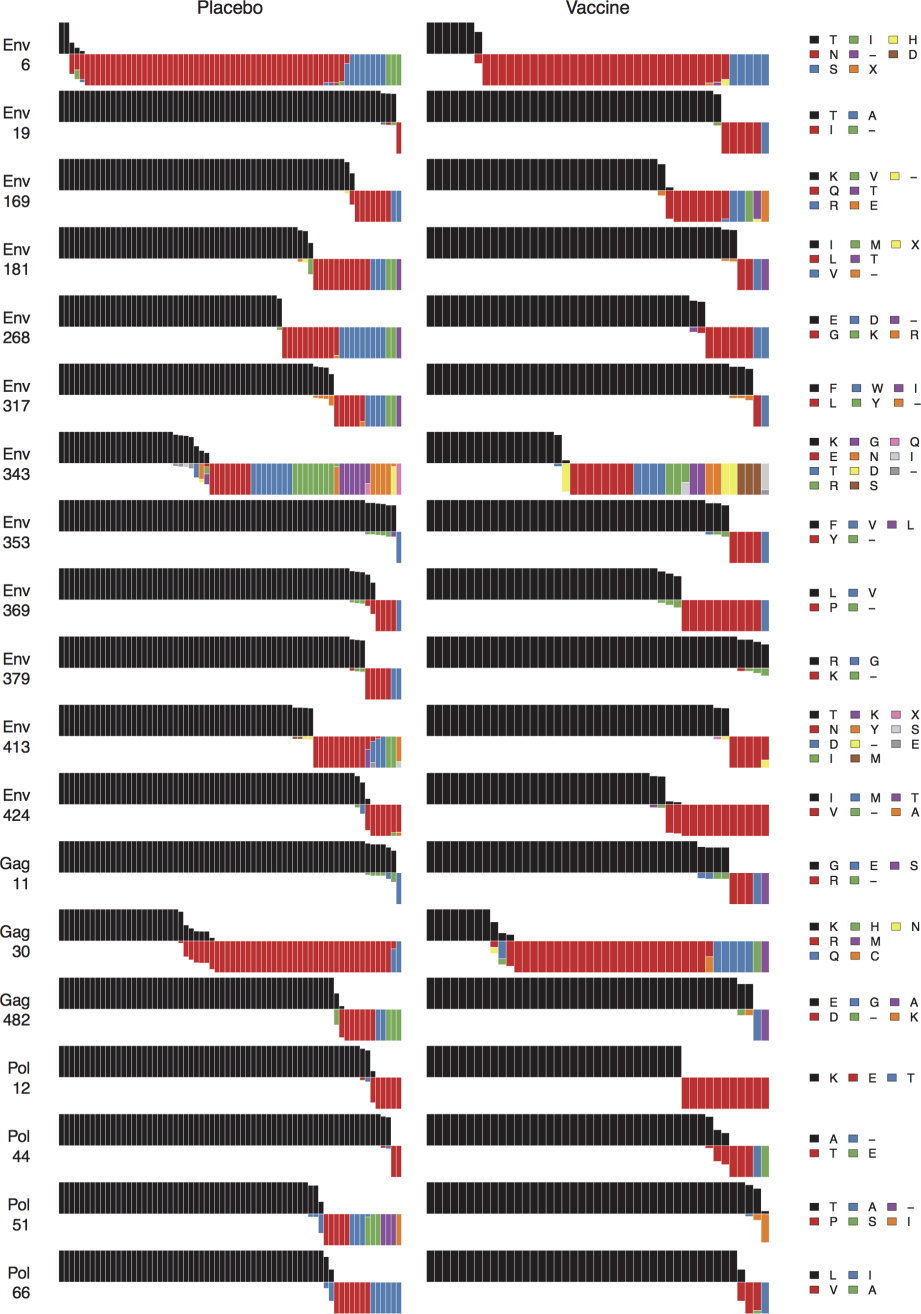
Vaccine protein signature sites AA distributions. For the vaccine protein signature sites shown in Fig. 2, Fig. 3 shows distributions of amino acids relative to the vaccine sequences for vaccine versus placebo recipient sequences: Each subject is represented by a bar. Bars all have equal height. The vaccine sequence AA residue, in black, is shown above the midline. Within a bar, colors depict the fraction of the subject’s sequences with that AA residue (or insertion or deletion, indicated by a “−“). The widths of the bars are scaled so that the total width of the vaccine-recipient part of the plot is the same as for the placebo-recipient part.

**Figure 4:**
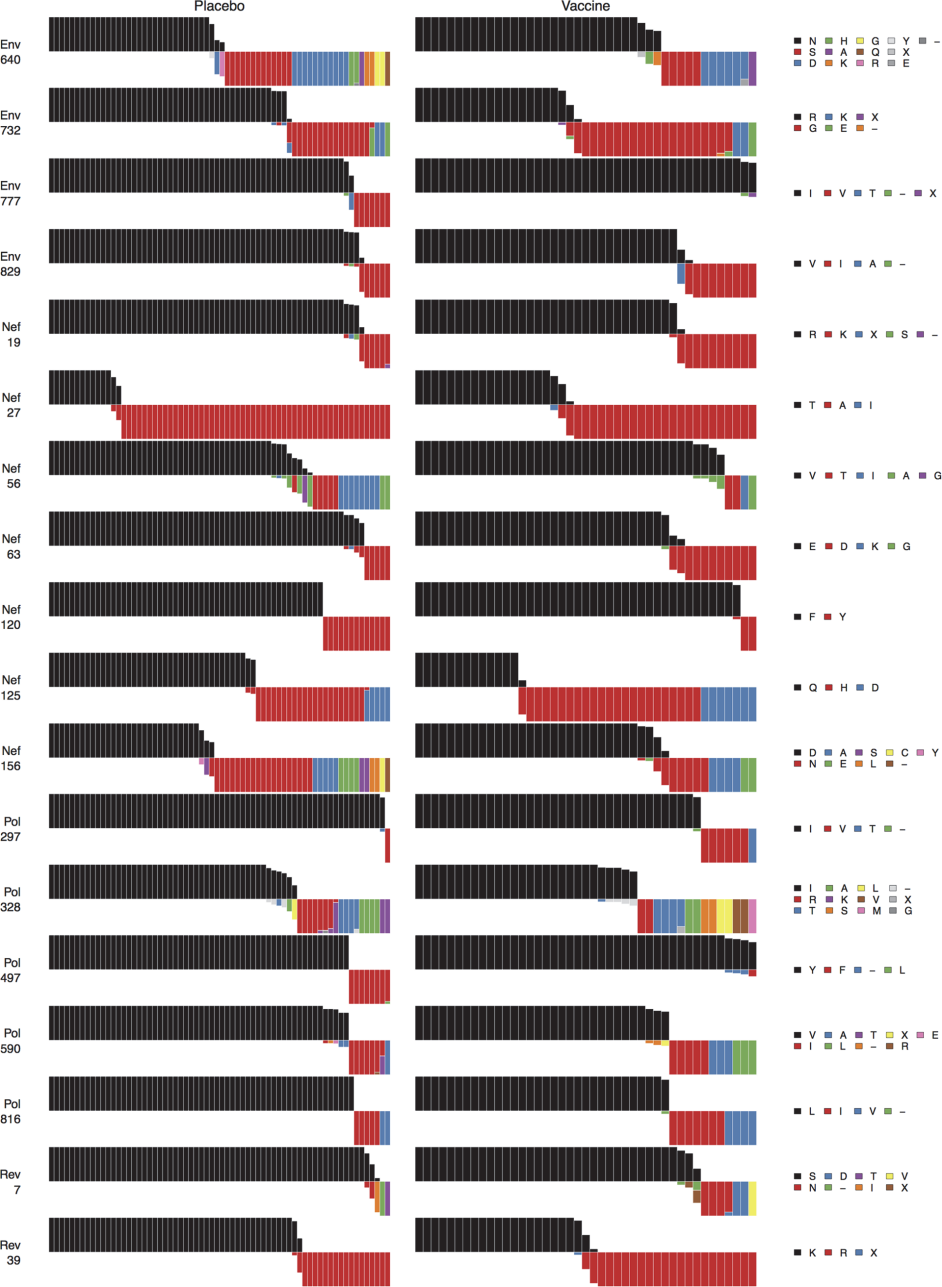
Non-vaccine protein signature sites AA distributions: First half. For the first half of the non-vaccine protein signature sites shown in Fig. 2, Fig. 4 shows distributions of amino acids relative to the vaccine sequences for vaccine versus placebo recipient sequences: Each subject is represented by a bar. Bars all have equal height. The vaccine sequence AA residue, in black, is shown above the midline. Within a bar, colors depict the fraction of the subject’s sequences with that AA residue (or insertion or deletion, indicated by a “−“). The widths of the bars are scaled so that the total width of the vaccine-recipient part of the plot is the same as for the placebo-recipient part.

**Figure 5:**
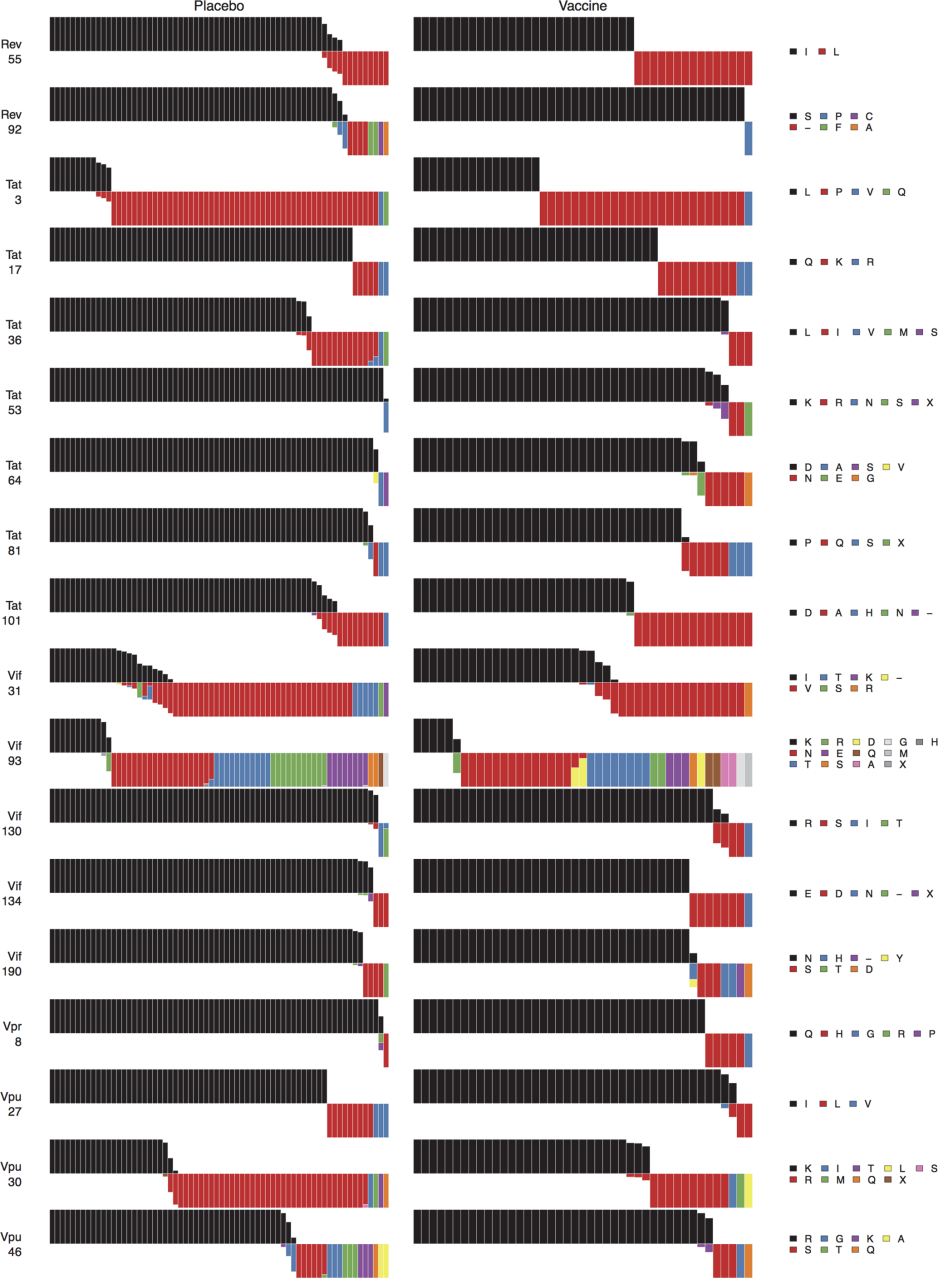
Non-vaccine protein signature sites AA distributions: Second half. For the second half of the non-vaccine protein signature sites shown in Fig. 2, Fig. 5 shows distributions of amino acids relative to the vaccine sequences for vaccine versus placebo recipient sequences: Each subject is represented by a bar. Bars all have equal height. The vaccine sequence AA residue, in black, is shown above the midline. Within a bar, colors depict the fraction of the subject’s sequences with that AA residue (or insertion or deletion, indicated by a “−“). The widths of the bars are scaled so that the total width of the vaccine-recipient part of the plot is the same as for the placebo-recipient part.

Our dataset includes genome sequences from 109 of the 110 subjects previously identified with HIV-1 CRF01_AE infections [7]. All sequences were obtained from the earliest available sample for sequencing, and all were prior to the subjects’ initiation of antiretroviral therapy. There were a median of 10 sequences available for analysis per subject in vaccine proteins (S1 Table) and also a median of 10 in non-vaccine proteins (S2 Table). We found 19 signature AA sites contained within the vaccine immunogens (Fig. 2) – 12 in Env-gp120, 3 in Gag and 4 in Pro – that showed a p-value ≤ 0.05 in at least one of the three primary site-scanning methods (Table 1). In addition, proteins that were not included in the vaccine immunogens were scanned for sieve effects versus the consensus CRF01_AE sequence (CON-AE) [12]: 37 sites were significant by at least one of the primary methods, and these were distributed across all non-vaccine proteins (Table 2 and Fig. 2; complete results in S1 Dataset). Four pairs of sites overlap different proteins across reading frames: one pair in the immunogen region, three pairs outside of the immunogen region. These overlapping sites are described in the “context” columns in Tables S3 and S4. 10 of the 19 immunogen signature sites were more likely to match a vaccine immunogen AA in the placebo group (a “vMatch” or “typical” sieve effect), and 10 of 19 in the vaccine group (a “vMismatch” or “atypical” sieve effect), with one site (Env 369) having both a vMatch effect vs the CRF AE immunogen AA and a vMismatch effect versus the subtype B immunogen AA, see Fig. 3; additional information about each site is provided in S3 Table (see Figs. 4 and 5 and S4 Table for non-immunogen signature sites). In contrast to the hypothesis-driven V1/V2 study, in this exploratory analysis we used uncorrected p-values at the 0.05 significance level to identify putative signature sites, a strategy taken to maximize sensitivity. To control for false positives, we used a conventional 0.20 false discovery rate (FDR) significance threshold [13], evaluated separately by gene for each analysis method. Only 1 of the 19 signature sites within the immunogen region, Pol 51, had q-value < 0.20 (Table 1, Fig. 2).

**Table 1:** Signature sites in vaccine proteins: 2-sided unadjusted p-values calculated by five methods.

**Position** **^1^**	**Ref:AA** **^2^**	**DVE**	**GWJ**	**MBS**	**EGWJ**	**MMB**
Env 6	92TH:(T)	0.027	0.026	0.011	0.72	0.027
Env 19	92TH:T	0.032	0.029	0.029	0.006	0.006
Env 169	BothAE:K	0.039	0.028	0.062	0.017	0.16
Env 181	All:(I)	0.027	0.02	0.02	0.049	0.042
Env 268	All:(E)	0.07	0.056	0.035	0.133	0.088
Env 317	All:(F)	0.04	0.038	0.027	0.034	0.037
Env 343	BothAE:K; MN:R	K:0.89; R:0.15	K:0.73; R:0.084	K:0.040; R:0.008	K:0.38; R:0.061	K:0.82; R:0.26
Env 353	All:F	0.095	0.118	0.049	0.019	0.04
Env 369	BothAE:L; MN:(P)	L:0.026; P:0.027	L:0.044; P:0.046	L:0.039; P:0.038	L:0.007; P:0.24	L:0.004; P:0.005
Env 379	BothAE:(R)	ND^3^	0.048	0.053	0.047	0.04
Env 413	92TH:(T)	0.08	0.06	0.033	0.101	0.081
Env 424	All:I	0.013	0.018	0.022	0.005	0.011
Gag 11	LAI:G	0.032	0.009	0.018	0.011	0.017
Gag 30	LAI:K	0.065	0.034	0.016	0.022	0.04
Gag 482	LAI:(E)	0.04	0.026	0.526	0.057	0.072
Pol 12	LAI:K	0.026	0.04	0.042	0.014	0.032
Pol 44	LAI:A	0.051	0.049	0.052	0.018	0.038
Pol 51^4^	LAI:(T)	0.014	0.002	0.003	0.003	0.004
Pol 66	LAI:(L)	0.056	0.072	0.042	0.108	0.22

^1^HXB2 numbering; all variable sites were analyzed, these included: Env [248 total; 235 92TH023, 200 CM244, 168 MN]; Gag [143], Pol [62]

^2^A sieve effect was detected for the indicated vaccine sequence; for Env positions, these are 92TH023, CM244, both of the CRF01_AE sequences, the subtype B sequence MN, or all three. The corresponding vaccine amino acids are given after the colon. They are in parentheses if the effect was “vMismatch” (with greater divergence from the vaccine AA among placebo recipients).

^3^The p-value for Env 379 was not calculable for the DVE method because one of the treatment groups (the vaccine-recipient group) exhibited no variation at the site.

^4^The signature at Pol 51 was the only vaccine-protein site with site-scanning results that passed the pre-specified false discovery rate (FDR) q-value threshold of 0.20: Pol 51 GWJ q = 0.15, MBS q = 0.16, EGWJ q = 0.195.

**Table 2:** Signature sites in non-vaccine proteins: 2-sided unadjusted p-values calculated by five methods.

**Positions Analyzed**	**Position** **^1^**	**Ref:AA** **^2^**	**DVE**	**GWJ**	**MBS**	**EGWJ**	**MMB**
Env: 165	Env 640	ConAE:(N)	0.03	0.022	0.011	0.089	0.011
	Env 732	ConAE:R	0.005	0.004	0.012	0.005	0.008
	Env 777	ConAE:(I)	ND^3^	0.033	0.03	0.033	0.037
	Env 829	ConAE:V	0.048	0.056	0.058	0.029	0.07
Nef: 93	Nef 19	ConAE:R	0.048	0.076	0.079	0.026	0.045
	Nef 27	ConAE:(T)	0.012	0.019	0.02	0.079	0.017
	Nef 56	ConAE:(V)	0.02	0.018	0.015	0.058	0.046
	Nef 63	ConAE:E	0.045	0.073	0.076	0.011	0.038
	Nef 120	ConAE:(F)	0.04	0.044	0.044	0.043	0.035
	Nef 125	ConAE:Q	0.002	0.002	0.002	<0.001	0.004
	Nef 156	ConAE:(D)	0.011	0.012	0.003	0.031	0.02
Pol: 170	Pol 297	ConAE:I	0.02	0.01	0.013	0.002	0.005
	Pol 328	ConAE:I	0.399	0.528	0.01	0.28	0.4
	Pol 497	ConAE:(Y)	ND^3^	0.033	0.03	0.036	0.046
	Pol 590	ConAE:V	0.076	0.043	0.081	0.039	0.075
	Pol 816	ConAE:L	0.045	0.051	0.063	0.023	0.011
Rev: 81	Rev 7	ConAE:S	0.027	0.023	0.007	0.014	0.031
	Rev 39	ConAE:K	0.007	0.007	0.007	0.005	0.004
	Rev 55	ConAE:I	0.019	0.032	0.031	0.012	0.046
	Rev 92	ConAE:(S)	0.078	0.028	0.069	0.054	0.034
Tat: 47	Tat 3	ConAE:(L)	0.029	0.03	0.029	0.32	0.011
	Tat 17	ConAE:Q	0.024	0.035	0.058	0.009	0.052
	Tat 36	ConAE:(L)	0.029	0.022	0.023	0.038	0.027
	Tat 53	ConAE:K	0.054	0.052	0.037	0.028	0.06
	Tat 64	ConAE:D	0.027	0.033	0.008	0.009	0.015
	Tat 81	ConAE:P	0.027	0.042	0.021	0.008	0.007
	Tat 101	ConAE:D	0.032	0.053	0.053	0.021	0.055
Vif: 76	Vif 31	ConAE:(I)	0.003	0.002	0.027	0.084	0.014
	Vif 93	ConAE:K	0.47	0.354	0.029	0.068	0.18
	Vif 130	ConAE:R	0.095	0.17	0.048	0.08	0.48
	Vif 134	ConAE:E	0.027	0.029	0.031	0.009	0.11
	Vif 190	ConAE:N	0.092	0.084	0.028	0.066	0.142
Vpr: 36	Vpr 8	ConAE:Q	0.032	0.025	0.028	0.008	0.01
Vpu: 51	Vpu 27	ConAE:(I)	0.056	0.068	0.046	0.079	0.18
	Vpu 30	ConAE:(K)	0.001	0.001	0.029	0.006	0.001
	Vpu 46	ConAE:(R)	0.04	0.042	0.025	0.067	0.101

^1^HXB2 numbering

^2^The sieve effect was detected for the CRF01_AE consensus sequence. The corresponding vaccine amino acids are given after the colon. They are in parentheses if the effect was “vMismatch” (with greater divergence from the vaccine AA among placebo recipients).

^3^The p-values for Env 777 and Pol 497 were not calculable for the DVE method because one of the treatment groups (the vaccine-recipient group) exhibited no variation at these sites.

The first of the three primary site-scanning sieve analysis methods, differential vaccine efficacy (DVE), uses Cox survival analysis to test whether vaccine efficacy (VE) for preventing infection by viruses that are AA-matched to the vaccine immunogen sequence at a particular locus is significantly different from the VE versus mismatched infections, [14,15]. Point estimates of VE associated with each mutation at which the DVE is significant show that VE can be eliminated or greatly strengthened with the mutation of a single residue (Table 3 and Table 4). Because this method evaluates all trial time-to-event data (including all randomized subjects HIV negative at baseline) and yielded a p-value for differential VE close to that for testing overall VE, the strength of evidence is comparable to the strength of evidence for overall efficacy, with the important caveat that multiple testing could lead to false discoveries due to chance variation. The other two primary methods compare the AA distribution of breakthrough infections at an individual site. Both methods employ numeric weights determined by the substitution frequency of the immunogen AA to the breakthrough sequence AA [16]. The Gilbert, Wu, Jobes (GWJ) method compares these substitution weights across treatment groups [17], and the Model-Based Sieve (MBS) method employs the weights in a Bayesian model comparison that is more sensitive to detect treatment effects that alter the distribution among non-vaccine-matched amino acid categories [18]. These three primary methods evaluate a single representative sequence (the *mindist* sequence) per subject. This sequence, chosen as the closest actual sequence to the consensus of a subject’s multiple sequences (S1 Text), is selected to represent the founder of the subject’s infection. To more fully represent the viral population, two secondary site-scanning methods utilize all available sequence data: the Mismatch Bootstrap (MMBootstrap, or simply MMB) method, which compares the frequency of vaccine-mismatched AAs in all of the subjects’ sequences across treatment groups (this is the method employed previously in the V2-focussed analysis [7]), and the new Expected GWJ (EGWJ) method that generalizes the GWJ method by replacing subject weights with weight averages over a subject’s multiple sequences (Table 1 and Table 2). Only 9 of the 19 sites identified in proteins represented in the vaccine were significant with all five scanning methods: five in Env (19, 181, 317, 369, 424), along with site 11 in Gag and sites 12, 44 and 51 in Pro, reflecting the variety of alternative hypotheses tested by the five methods (Fig. 1).

**Table 3:** Vaccine efficacy at the signature sites in the vaccine proteins.

**Position** **^1^**	**Ref:AA** **^2^**	**DVE p-value**	**VE vs Match p-value**	**VE** **^3^** **vs Match Estimate (95% CI)**	**VE vs Mismatch p-value**	**VE** **^3^** **vs Mismatch Estimate (95% CI)**	
Env 6	92TH:(T)	0.027	0.097	−71% (−94% to 28%)	0.005	44% (16% to 63%)
Env 19	92TH:T	0.032	0.005	43% (15% to 62%)	0.06	−83% (−98% to 28%)
Env 169	BothAE:K	0.039	0.004	48% (18% to 66%)	0.4	−30% (−70% to 39%)
Env 181	All:(I)	0.027	0.377	17% (−21% to 46%)	0.003	78% (35% to 93%)
Env 268	All:(E)	0.07	0.355	19% (−21% to 48%)	0.007	65% (23% to 85%)
Env 317	All:(F)	0.04	0.208	23% (−14% to 49%)	0.004	85% (32% to 97%)
Env 343	BothAE:K; MN:R	K:0.887; R:0.146	K:0.128; R:0.034	K:37% (−13% to 66%); R:78% (−2% to 95%)	K:0.103; R:0.102	K:34% (−8% to 60%); R:28% (−7% to 52%)
Env 353	All:F	0.095	0.009	41% (12% to 60%)	0.26	−60% (−92% to 52%)
Env 369	BothAE:L; MN:(P)	L:0.026; P:0.027	P:0.200; L:0.003	P:−50% (−83% to 32%); L:47% (18% to 65%)	P:0.004; L:0.229	P:46% (18% to 65%); L:−45% (−80% to 33%)
Env 379	BothAE:(R)	ND**^4^**	0.108	27% (−7% to 51%)	0.008	100% (−100% to 100%)
Env 413	92TH:(T)	0.08	0.231	23% (−15% to 49%)	0.01	71% (21% to 89%)
Env 424	All:I	0.013	0.002	49% (21% to 67%)	0.183	−46% (−78% to 26%)
Gag 11	LAI:G	0.032	0.005	43% (15% to 62%)	0.059	−83% (−98% to 28%)
Gag 30	LAI:K	0.065	0.006	64% (23% to 83%)	0.408	17% (−23% to 48%)
Gag 482	LAI:(E)	0.04	0.208	23% (−14% to 49%)	0.004	85% (32% to 97%)
Pol 12	LAI:K	0.026	0.003	47% (18% to 65%)	0.229	−45% (−80% to 32%)
Pol 44	LAI:A	0.051	0.007	42% (14% to 62%)	0.159	−67% (−93% to 40%)
Pol 51	LAI:(T)	0.014	0.393	16% (−21% to 44%)	<0.001	94% (53% to 99%)
Pol 66	LAI:(L)	0.056	0.204	23% (−14% to 49%)	0.007	79% (26% to 94%)

^1^HXB2 numbering

^2^The sieve effect was detected for the indicated vaccine sequence; for Env positions, these are 92TH023, CM244, both of the CRF01_AE sequences, the subtype B sequence MN, or all three. The corresponding vaccine amino acids are given after the colon. They are in parentheses if the effect was “vMismatch” (with greater divergence from vaccine AA among placebo recipients).

^3^Symmetrized estimated vaccine efficacies (for hazard ratio (HR) above 1, symmetrized VE = [1 − hazard ratio (HR)]×100%; for HR below 1, symmetrized VE = − [1 − (1/(HR))]×100%) to prevent infection with specific HIV-1 genotypes.

^4^The p-value for Env 379 was not calculable for the DVE method because one of the treatment groups (the vaccine-recipient group) exhibited no variation at the site.

**Table 4:** Vaccine efficacy at the signature sites in the non-vaccine proteins.

**Position** **^1^**	**Ref:AA** **^2^**	**DVE p-value**	**VE vs Match p-value**	**VE** **^3^** **vs Match Estimate (95% CI)**	**VE vs Mismatch p-value**	**VE** **^3^** **vs Mismatch Estimate (95% CI)**
Env 640	ConAE:(N)	0.03	0.786	7% (−35% to 43%)	0.002	62% (28% to 80%)
Env 732	ConAE:R	0.006	<0.001	61% (33% to 77%)	0.557	−16% (−54% to 34%)
Env 777	ConAE:(I)	ND**^3^**	0.13	26% (−9% to 50%)	0.005	100% (−100% to 100%)
Env 829	ConAE:V	0.048	0.005	45% (16% to 64%)	0.321	−40% (−78% to 40%)
Nef 19	ConAE:R	0.048	0.005	45% (16% to 64%)	0.321	−40% (−78% to 40%)
Nef 27	ConAE:(T)	0.012	0.39	−26% (−63% to 32%)	0.001	54% (25% to 72%)
Nef 56	ConAE:(V)	0.02	0.378	17% (−21% to 46%)	0.002	79% (38% to 93%)
Nef 63	ConAE:E	0.045	0.004	46% (17% to 65%)	0.35	−36% (−75% to 39%)
Nef 120	ConAE:(F)	0.04	0.208	23% (−14% to 49%)	0.004	85% (32% to 97%)
Nef 125	ConAE:Q	0.002	<0.001	68% (39% to 83%)	0.603	−13% (−49% to 32%)
Nef 156	ConAE:(D)	0.011	0.988	0% (−39% to 39%)	0.001	66% (34% to 82%)
Pol 297	ConAE:I	0.02	0.004	45% (17% to 63%)	0.034	−86% (−98% to 14%)
Pol 328	ConAE:I	0.502	0.021	42% (7% to 64%)	0.472	23% (−36% to 61%)
Pol 497	ConAE:(Y)	ND**^4^**	0.13	26% (−9% to 50%)	0.005	100% (−100% to 100%)
Pol 590	ConAE:V	0.076	0.006	45% (15% to 64%)	0.496	−27% (−71% to 45%)
Pol 816	ConAE:L	0.045	0.004	46% (17% to 65%)	0.351	−36% (−75% to 39%)
Rev 7	ConAE:S	0.047	0.004	45% (16% to 63%)	0.209	−57% (−89% to 40%)
Rev 39	ConAE:K	0.01	0.001	58% (30% to 75%)	0.537	−18% (−56% to 35%)
Rev 55	ConAE:I	0.019	0.002	50% (22% to 68%)	0.322	−33% (−70% to 33%)
Rev 92	ConAE:(S)	0.078	0.126	27% (−9% to 51%)	0.011	89% (13% to 99%)
Tat 3	ConAE:(L)	0.029	0.456	−25% (−64% to 37%)	0.003	50% (21% to 69%)
Tat 17	ConAE:Q	0.024	0.003	48% (19% to 66%)	0.255	−41% (−77% to 33%)
Tat 36	ConAE:(L)	0.029	0.283	20% (−17% to 47%)	0.003	81% (36% to 95%)
Tat 53	ConAE:K	0.091	0.007	42% (13% to 61%)	0.182	−75% (−97% to 56%)
Tat 64	ConAE:D	0.027	0.005	44% (16% to 63%)	0.097	−71% (−94% to 28%)
Tat 81	ConAE:P	0.027	0.004	45% (17% to 64%)	0.169	−55% (−86% to 31%)
Tat 101	ConAE:D	0.032	0.003	49% (20% to 68%)	0.439	−26% (−66% to 38%)
Vif 22	ConAE:N	0.046	0.005	46% (16% to 65%)	0.377	−33% (−73% to 39%)
Vif 31	ConAE:(I)	0.003	0.373	−24% (−58% to 28%)	<0.001	62% (34% to 78%)
Vif 93	ConAE:K	0.47	0.132	55% (−23% to 84%)	0.075	31% (−4% to 54%)
Vif 130	ConAE:R	0.095	0.009	41% (12% to 60%)	0.259	−60% (−92% to 52%)
Vif 134	ConAE:E	0.027	0.004	45% (16% to 63%)	0.134	−62% (−90% to 30%)
Vif 190	ConAE:N	0.092	0.008	43% (13% to 62%)	0.41	−37% (−79% to 48%)
Vpr 8	ConAE:Q	0.032	0.005	43% (15% to 62%)	0.06	−83% (−98% to 28%)
Vpu 27	ConAE:(I)	0.056	0.176	24% (−12% to 50%)	0.007	83% (26% to 96%)
Vpu 30	ConAE:(K)	0.001	0.424	−20% (−53% to 27%)	<0.001	69% (43% to 83%)
Vpu 46	ConAE:(R)	0.04	0.32	19% (−19% to 47%)	0.004	74% (30% to 90%)

^1^HXB2 numbering

^2^The sieve effect was detected for the CRF01_AE consensus sequence. The corresponding vaccine amino acids are given after the colon. They are in parentheses if the effect was “vMismatch” (with greater divergence from the vaccine AA among placebo recipients).

^3^Symmetrized estimated vaccine efficacies (for hazard ratio (HR) above 1, symmetrized VE = [1 − hazard ratio (HR)]×100%; for HR below 1, symmetrized VE = − [1 − (1/(HR))]×100%) to prevent infection with specific HIV-1 genotypes.

^4^The p-values for Env 777 and Pol 497 were not calculable for the DVE method because one of the treatment groups (the vaccine-recipient group) exhibited no variation at these sites.

In a related analysis, we used 9-mer scanning (the KmerScan method as previously described [8]) to compare all 9-mers in subjects’ sequences to the corresponding 9-mer in each reference sequence (the vaccine sequences for immunogen proteins and the CRF01_AE consensus sequence, CON-AE, for non-immunogen proteins). This analysis evaluates contiguous amino acids that could be the target of a CTL response, but does so without incorporating subject-specific HLA information. For a given pair of 9-mers the similarity score was the sum of HIVb similarity scores [16] over the nine sites. We compared the distribution of these scores for all of the individual sequences between the infected vaccine group and the infected placebo group. S5 Table lists 9-mers that had significantly different similarity to a vaccine immunogen sequence 9-mer across treatment groups (38 9-mers) and S6 Table lists 9-mers outside of the vaccine immunogen regions that significantly differed versus CON-AE (82 9-mers). Thirty 9-mers in Tat (involving sites 1–72) and one 9-mer in Vpu (sites 30–38) passed the pre-specified 20% q-value multiplicity adjustment threshold. The significant 9-mers in Tat comprised seven distinct contiguous regions ranging in length from 9 to 25 AA. In four of these seven regions there was a signature site that could explain the 9-mer scanning results (with concordance of “vMatch” or “vMismatch” sieve effects) while in three of the regions at least one of the 9-mers did not overlap a signature site.

### No clear evidence that vaccine efficacy declined with genetic distances to antibody-relevant regions of the immunogens, and increased evidence for some vaccine efficacy

We sought to test the hypothesis that vaccine efficacy declined as a function of the distance between the HIV-1 breakthrough viruses and the immunogen sequences. The SmoothMarks method [19,20] (S2 Text) evaluates VE as a continuous function of each of several distances between the *mindist* sequences and each immunogen sequence. In addition to distances corresponding to all gp120 sites, we considered four of the pre-specified immunologically-relevant subsets of gp120 amino acid sites: *contactsites, contactsites-augmented, hotspots, EPIMAP* (Table 5). For all of these analyses the first 41 sites of Env were excluded, because they were not present in CM244 and MN and the first approximately 30 sites corresponded to the signal peptide, which is cleaved from the mature protein. S1 Fig. shows boxplots of the genetic distances for the vaccine and placebo groups for the five sets of sites against the two CRF01_AE vaccine sequences (92TH023 and CM244), computed using the HIVb substitution matrix. These distances are tightly correlated with Hamming distances (percent amino acid mismatch), with Spearman rank correlations ranging between 0.91 and 0.95 across the 10 distances. The distances are approximately equal when measured to the 92TH023 and CM244 reference sequences, and all of the distances except *hotspots* are approximately equal across the sets, whereas the *hotspots* distances tend to be lower. The median (range) number of amino acid mismatches to the reference sequences are 13.4 (4.3–24.6) per 100 sites for all of the distances except the *hotspots* distances, and are 9.2 (3.6–14.8) per 100 sites for the *hotspots* distances. The likely reason for the closer *hotspots* distances is that the linear peptides used to measure antibody binding reactivity included 7 distinct HIV-1 subtypes, indicating that *hotspots* sites are sites with cross-subtype-reactivity, and such sites are expected to be relatively conserved because the vaccine can more readily induce cross-reactive antibodies to more conserved peptides. While the hypothesis testing analyses presented next are of main interest given they assess vaccine efficacy directly, we note that the distances between HIV-1 breakthrough and immunogen sequences did not significantly differ between infected vaccine recipients and infected placebo recipients.

**Table 5:** Immunologically relevant subsets of sites.

**Subset**	**No. Sites**	**Definition**
Env-immunogen	248	Alignable sites in a vaccine sequence, with sufficient variability
Gag-immunogen	143	Alignable sites in LAI sequence, with sufficient variability
Pol-immunogen	62	Alignable sites in LAI sequence, with sufficient variability
*Contactsites*	99	Contact residues for monoclonal antibodies [54,55]
*nAb-sites*	37	Additional sites known to affect antibody neutralization from published literature [54–56]
*contactsites-augmented*	112	Union of *contactsites* and *nAb-sites*
*Hotspots*	96	Antibody binding reactivity for RV144 vaccine recipients based on peptide microarrays [6]
*EPIMAP*	37	Structural biology predictions of conformational antibody epitope contact sites [7]
*Focus*	40	Intersection of *hotspots* and *contact sites-augmented*

For each site set we provide a brief description with references, as well as numbers of sites in each set that were alignable, sufficiently variable, and that corresponded to a vaccine immunogen sequence locus.

**Figure 6:**
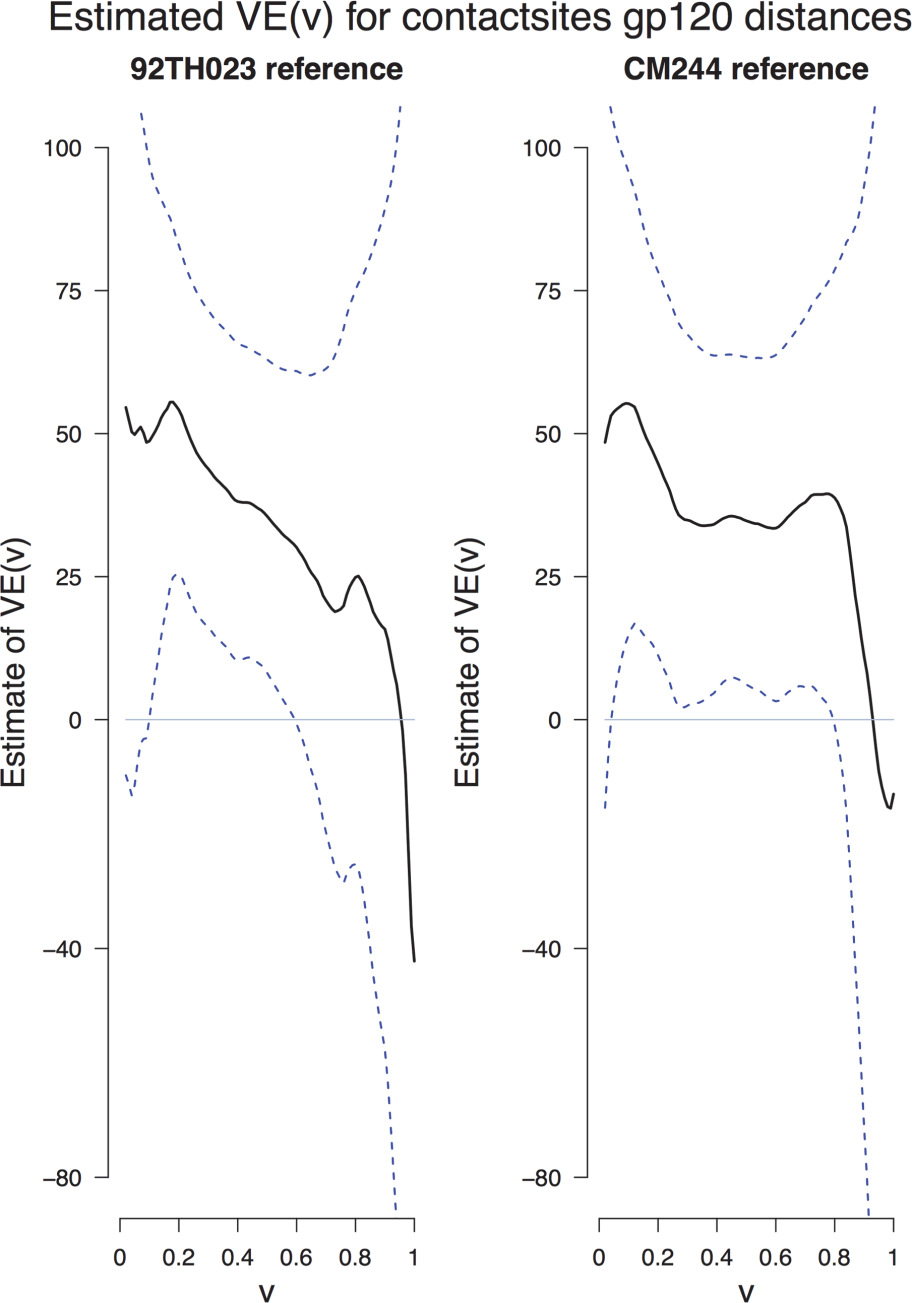
Estimated vaccine efficacy as a function of distance to *contactsites* of the CRF01_AE vaccine sequences. SmoothMarks [20] estimates of vaccine efficacy (VE) against acquisition with an HIV-1 CRF01_AE virus with genetic distance *v* from the 92TH023 or CM244 vaccine sequences, with 95% confidence intervals, using Env *mindist* amino acid sequences and computed with the HIVb PAM substitution matrix[16] across the *contactsites*. For each panel, the first p-value is for testing whether there is any VE against any virus genotype, and the second p-value is for testing whether VE declines with the distance *v*. The PAM distances are directly proportional to Hamming distances, where a PAM distance of *v* approximately equals a Hamming distance of 0.85×*v*. Given that *contactsites* contains 176 residues, the span of *contactsites* distances 0.08 to 0.25 correspond to 13–39 amino acid mismatches.

Vaccine efficacy was estimated as a function of genetic distance *v* for each of the ten distances (Fig. 6 for *contactsites*, S2 Fig. for all ten distances). A similar analysis of a subset of these antibody contact sites was previously reported in Gilbert and Sun [20], using a set of monoclonal antibody contact sites that was current through 2011; here we analyzed distances of Ab contact sites based on information that is up-to-date as of August 2014. The tests for distance-variability of vaccine efficacy were all non-significant (p-values > 0.20). These results support no strong sieve effects but cannot rule out moderate sieve effects, as power calculations showed that for the setting of the RV144 trial, the SmoothMarks method has only 50% power to detect VE declining from 67% to 0%. However, these distance-based analyses contribute additional evidence supporting the hypothesis that the vaccine regimen conferred some protection. In particular, overall vaccine efficacy against CRF01_AE HIV-1 ignoring the genetic distances resulted in a p-value for positive VE of 0.026, whereas the tests of Gilbert and Sun [20] for positive vaccine efficacy against at least one HIV-1 genotype (10 tests) gave p-values ranging from 0.006 to 0.024, with median p = 0.013. This shows that accounting for the genetic distances increased the evidence for positive vaccine efficacy against CRF01_AE HIV-1.

### Combined impact of the Env-gp120 signature sites on vaccine efficacy

To complement the site-scanning sieve analysis that identified individual Env-gp120 signature sites as potential discriminators of vaccine efficacy, we combined the signature sites into a global distance and assessed how the vaccine efficacy varied with this distance. In particular, the global sieve analysis above was repeated for the distances calculated over just the 10 identified Env-gp120 signature sites (listed in Table 1), excluding Env 6 and Env 19 because they are not in the CM244 and MN inserts and they are part of the signal peptide. Because 5 of the 10 signature sites had “vMatch” sieve effects and 5 had “vMismatch” sieve effects, we do not expect the vaccine efficacy curves to exhibit the “classical” sieve effect shape with vaccine efficacy highest for smallest distances and waning to zero for the greatest distances; rather the vaccine efficacy curves could take many shapes depending on the joint distribution/covariation of the amino acids at the 10 sites, and the curves provide new information about the aggregate impact of the non-contiguous decapeptides on vaccine efficacy.

S3 Fig. shows the distributions of the signature-site distances to 92TH023 and CM244 together with the estimated vaccine efficacy curves. For 92TH023, the estimated curve is approximately horizontal, indicating that the 5 “vMatch” and 5 “vMismatch” signature sites have a “balancing” effect, with the net impact being that the combined decapeptide patterns do not associate with vaccine efficacy. However, for CM244 the estimated VE peaks against HIV-1s with an intermediate number of mismatches to the vaccine (zenith at estimated VE = 59% for genetic distance 0.28, an average of 2 mismatched residues) and declines to zero against HIV-1s with increasing distance (estimated VE = 0% for genetic distance 0.53, an average of 5 mismatched residues). To help interpret this relationship, S4 Fig. shows the signature site decapeptide AA patterns for each of the 109 infected subjects, aligned to the vaccine efficacy curve for reference. S4 Fig. indicates that the “vMismatch” signature site Env 413 has the greatest influence to create the increasing VE curve in the distance region 0.066 to 0.166 and the vMismatch signature sites Env413, Env 268, and Env 317 have the greatest influence to create the declining VE curve in the distance region 0.28 to 0.53.

### Physicochemical property differences in Env-gp120, Pro, Gag and non-vaccine proteins

To search for functional sequence differences in the vaccine and placebo groups, we evaluated treatment-group differences in the physicochemical properties of amino acids in the *mindist* sequences. Unlike the methods with results presented in Table 1 and Table 2, which compare divergences of breakthrough AA from a vaccine AA between treatment groups, the physicochemical properties (PCP) method compares the sequences between groups directly, without regard for the vaccine reference sequences, on a per-property basis. We evaluated each of two different property scales: (1) the vector of ten indicator values determined by Taylor [21], indicating the presence or absence of ten particular physicochemical properties for each amino acid; and (2) the vector of five “z scales” for each amino acid, principal components of observed physicochemical properties used to determine quantitative structure-activity relationships between peptides [22–24]. We scanned the sequences at individual sites (S7 and S8 Table) as well as at contiguous 3-mers (S9 and S10 Table) and 9-mers (S11 and S12 Table) across the HIV-1 proteome, comparing counts of each of the ten Taylor properties and five z-scale components across treatment groups (complete results are included in S1 Dataset). The results of this method can help interpret the physicochemical and structural differences between the vaccine and placebo viral populations.

Of the 19 vaccine immunogen signature sites shown in Table 1, only Pol 51 was also found to have site-specific significant PCP results (S3 Table), and of the 37 out-of-immunogen signature sites shown in Table 2, eight coincided with site-specific PCP results (S4 Table). A total of 16 individual sites were significant at the p ≤ 0.05 level (S7 and S8 Table), two of which with q-values below 0.2: Pol 51 as noted above (property z3, q = 0.19) and Vpu 30 (hydrophobicity, q = 0.10). These two sites were also the only locations with q-values below 20% in the scanning of 3-mer peptides (Peptide starting at Pol 49: property z5, q = 0.024; Peptides starting at Vpu 28, 29 and 30: hydrophobicity, q = 0.050). Additional sites had q-values below 20% when scanning 9-mer peptides, all of which were located in the non-immunogen proteins, concordant with the KmerScan 9-mer results. A negatively charged region in the vicinity of Nef 150 differed between the treatment groups (q = 0.059) as did component z4 in the vicinity of Tat 73 (q = 0.19). In addition, hydrophobic residues in the vicinity of Vpu 30 spanning positions Vpu 25 through Vpu 30 differed between the treatment groups (the q-values in this region ranged from a minimum of q = 0.021 for the 9-mer starting at Vpu 25 to a maximum of q = 0.23 for the 9-mer starting at position Vpu 29).

**Figure 7:**
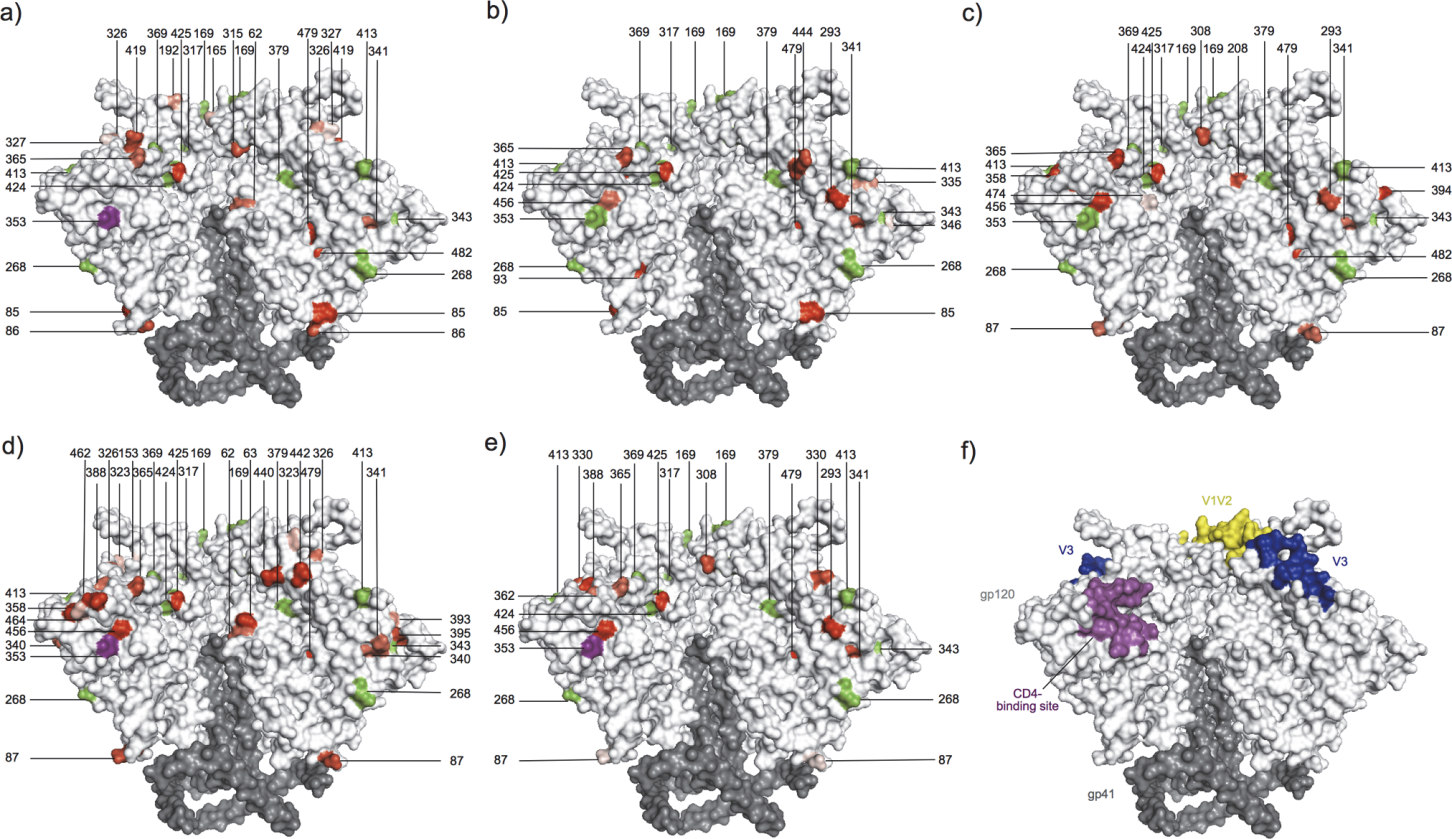
Mapping of signature sites and sites under selection on an Env trimer structure. Trimer (PDB id: 4NCO [27]) is shown in surface representation, with gp120 in grey and gp41 in dark grey. (a-e) Panels correspond to the five physico-chemical properties analyzed for evidence for property importance: (a) chemical composition, (b) polarity, (c) volume, (d) isoelectric point or (e) hydropathy [25]. Signature sites identified in Env-gp120 are colored in green, and sites that were under selection are colored from pink to red (corresponding p-values from 0.05 to < 0.0001). (f) Visualization of the major sites of vulnerability on the HIV-1 Env.

To further elucidate the role of selection for particular physicochemical properties, we conducted a codon-based phylogenetic analysis that detected Env-gp120 sites at which natural selection has operated to preserve or change one or more of five physicochemical properties: chemical composition, polarity, volume, iso-electric point or hydropathy [25,26]. To do so, we modeled the rate of nonsynonymous substitution from codon x to codon y, *β*(*x*, *y*) at a site as a function of the difference in properties between x and y: *β*(*x*, *y*) = exp(−∑_*p*_ *c*_*p*_ *d*_*p*_[*x*, *y*]), where p indexes the five properties. If *c_p_* is significantly different from 0 for a particular property *p* at a site (measured by a likelihood ratio test, with 5-fold multiple testing correction at each site using the Holm-Bonferroni procedure) along the vaccine-group lineages, then we conclude that the property is preserved (*c_p_* >0) or driven to change (*c_p_* <0) by natural selection. Fig. 7 shows the sites found to have selection acting on one or more properties, along with signature sites on a crystal structure of Julien et al.[27], and S5 Fig. provides an alternate viewing angle. Notably, at several sites almost all physicochemical properties tested were under selection, including Env 85–87, 353, 365, and 425 (S13 Table and S1 Dataset).

### Immunological relevance of signature sites found in Env-gp120

Many of the signature sites described above are located in genomic regions of known functional or immunological relevance. Specifically, those in HIV-1 Env-gp120 include sites in the antibody binding regions at the crowns of the V2 (Sites 169, 181) and V3 (Site 317) variable loops, sites in the co-receptor binding site (Sites 317, 353), and in the CD4 binding loop motif (Site 369). We considered six pre-specified subsets of Env sites known to be immunologically relevant (Table 5). We found that the Env-gp120 signature sites were significantly more likely to be found in a subset of Env sites known for their relevance to neutralization potency (*nAb-sites* set) (Fisher’s exact test p = 0.0035). Signature sites were also more likely to be part of the *focus* set that includes only those sites that were identified as *hotspots* of antibody binding reactivity in RV144 and are either in the known antibody *contactsites* set or are in the *nAb-sites* set (p = 0.0049). Results for all six site sets are presented in Table 6.

**Table 6:** Tests for enrichment of signature sites in biologically-defined subsets compared to all other sites.

**Subset**	**Signature sites found in the given subset, Fisher’s exact test**
*contactsites*	5 inside (5 of 99); 7 outside (7 of 354), p = 0.15
*nAb-sites*	**6 inside (6 of 37); 6 outside (6 of 416), p = 0.0001**
*contactsites-augmented*	**9 inside (9 of 112); 3 outside (3 of 341), p = 0.0003**
*Hotspots*	**7 inside (7 of 96); 5 outside (5 of 357), p = 0.0049**
*EPIMAP*	3 inside (3 of 37); 9 outside (9 of 416), p = 0.07
*Focus*	**5 inside (5 of 40); 7 outside (7 of 413), p = 0.0021**

For each of the six pre-specified site sets, we compared the number of the 12 vaccine immunogen signature sites in Env that are in the set to the number that are outside of the set, to test whether membership in the set is independent of the “signature site” designation. For example, 7 of the 12 signature sites are in the *Hotpots* site set, while 89 of the 441 tested non-signature sites are in that set.

Using tests for codon selection that identify important biochemical properties as discussed above, as well as a method that estimates the ratio of non-synonymous and synonymous substitution rates (dN/dS) separately in internal and terminal branches of the tree connecting these sequences [25], we found 91 sites in gp160 that were under differential selective pressure between the vaccine and the placebo groups (54 among the 511 sites of gp120 and 37 among the 345 sites of gp41, S13 Table and S1 Dataset), including signature sites Env 6 and Env 353. Interestingly, a high proportion of these sites were located in V3 (8/54, significantly more than the proportion of sites located outside of V3: Fisher’s exact test two-sided p-value = 0.049).

Covariation was assessed within proteins between pairs of sites, both pooling over the vaccine and placebo groups and separately, together with a test for whether the degree of covariation differed for vaccine versus placebo, which could imply vaccine-induced functional or structural constraints (S3 Text and S2 Dataset). Covariation was generally weak, and there was no evidence that covarying site pairs were restricted to the vaccine group. Among gp160 residues, there were 630 covarying site pairs with p < 0.05 but only two had a q-value below 0.2: between sites 276 and 343 (q = 0.10), and between sites 65 and 181 (q = 0.12). Both sites 181 and 343 were identified above as signature sites. An interesting pattern was seen when we considered how many associations (defined as treatment-arm-pooled covariation p < 0.05) linked each residue. While most Env sites (n = 584) showed no association with any other site, 22 sites interacted with more than five other sites (19, 169, 181, 276, 307, 308, 317, 332, 343, 347, 353, 360, 365, 369, 379, 412, 413, 424, 465, 564, 658, 822). Ten of these 22 sites were also signature sites, and there is evidence for a hub of covariation in V2 (S3 Text).

Importantly, the signature sites identified showed significantly more associations with other signature sites (mean = 32.13) than with non-signature sites (mean = 0.88) (p < 0.0001). This difference was also significant if we considered only gp120 (p < 0.0001). Interestingly, there were more associations between residues in gp120 (mean = 2.03), which corresponded to the vaccine sequence, than in gp41, which was not included in the vaccine (mean = 0.63) (p = 0.10; p = 0.001 if zero values are excluded).

### Evidence of T-cell sieve effects at class I HLA-associated binding Env epitopes

We have developed multiple methods to evaluate potential T cell-driven sieve effects based on comparisons of computationally-predicted epitopes in sequences from vaccine and placebo recipients. Results are shown in S14 and S15 Tables. For all methods, we begin by predicting T cell epitopes in the vaccine and breakthrough sequences using the HLA haplotypes of the infected trial participants.

First we evaluated each viral protein using the novel EscapeCount method (S4 Text), which counts the number of high-affinity predicted epitopes in the vaccine sequence that bind with much lower affinity to the corresponding k-mer in the subject’s *mindist* sequence. Since the power of this method is improved when there is more variability in the predicted epitope binding affinities, for class I predictions we used the adaptive double threading (ADT) epitope prediction software [28] rather than the more well-known NetMHCpan software [29] that we used in this and previous applications of the EpitopeDistance method (discussed below). Using the EscapeCount method, we found significant evidence of greater class I binding escape among placebo recipients in Env versus the CM244 reference sequence than among vaccine recipients (p = 0.031).

We also applied the EscapeCount method to evaluate individual k-mers for evidence of greater class I (9-mers) and class II (15-mers) binding escape in vaccine versus placebo groups (S14 and S15 Tables). Twelve 9-mers (ten in Env and two in Gag) showed an unadjusted p-value <0.05 for differential binding escape, though none of these surpassed the q-value threshold of 20% (0.24 < q-value < 0.93). Seven 9-mers with a q-value < 0.5 were found in Env-gp120 (start positions: 5, 128, 299, 328, 335, 363, 445). In S6 Fig. we show as an example the V3 loop 9-mer “PSNNTRTSI” (PI9, HXB2 start position 299) at which there was a greater number of HLA binding escapes in vaccine versus placebo recipients (p = 0.0084). Vaccine to placebo differences are concentrated in the 9^th^ position, site 307, which forms part of the core of the V3 loop. Although site 307 did not qualify as a signature site, its DVE p-value was 0.065 and its EGWJ p-value was significant at 0.029. This site forms (with Env 308 and 317) the core of the V3 loop and is a well-studied target of antibody binding [6,30]. Since also many of the infected RV144 subjects had HLA types capable of binding the CRF01_AE vaccine sequence epitope, and the variation at site 307 abrogated class I HLA binding in vaccine but not placebo recipients, the V3 sieve effect may be partially due to T cell mediated effects.

As the second of three methods, we applied the PercentEpitopeMismatch procedure, which was applied previously to the HVTN 502/Step sieve analysis [8] (S14 and S15 Tables). This method complements the EscapeCount method by considering any class I epitope that is predicted (for a given subjects’ HLA type) in the vaccine sequence, regardless of whether it is also predicted in the breakthrough sequence, and regardless of the number of changes between the breakthrough and vaccine k-mers. The percent of vaccine-predicted epitopes for which there is any change in the corresponding breakthrough sequence was computed for all of a subject’s sequences and these were compared across treatment groups as previously described [8] (S5 Text). Using the PercentEpitopeMismatch method, we found no evidence of T cell escape in insert-relevant genes when using the NetMHCpan- or ADT-predicted epitopes.

For the third method we applied the EpitopeDistance procedure that was also previously applied for the HVTN 502/Step trial [8]. This method compares the predicted epitopes in each subject’s breakthrough sequences to HLA-matched epitopes estimated in the vaccine sequence (S6 Text). In summary, we found no concordant evidence for a T cell-driven sieve effect across Gag, Pro and Env (S14 Table) or the non-vaccine proteins (S15 Table). However, some significant results were found in the V2 region of Env when binding affinities were considered (CM244 p = 0.022; 92TH023 p = 0.047), although there was only a trend suggesting a difference between the vaccine and placebo groups when evolutionary distances were considered (CM244 p = 0.058; 92TH023 p = 0.23).

### Vaccine-recipient breakthrough Gag sequences trend to greater phylogenetic divergence and diversity than placebo-recipient Gag sequences

To analyze sequences at the gene/protein level, we assessed whether sequences from vaccine recipients were (a) more phylogenetically diverse or (b) more divergent from the vaccine insert sequences than sequences found in placebo recipients. For the phylogenetic diversity, we constructed maximum-likelihood phylogenetic trees using all amino acid sequences available for each subject; we then subset the leaves of these trees to retain only the *mindist* sequences. For each tree, we then computed the differential amino acid phylogenetic diversity (PD) [31], defined as the difference in the total branch length of two subtrees: one corresponding to placebo recipients and the vaccine insert, and the complementary sub-tree corresponding to vaccine recipients and the vaccine insert, and compared this to an estimated null distribution as described in Methods. We found trending evidence of greater phylogenetic diversity in Gag for the vaccine group compared to the placebo group (p = 0.089) (S17 Table).

In addition to the phylogenetic diversity, we also calculated the phylogenetic divergence from the vaccine sequence based on the same *mindist* trees (S7 Fig. shows the distributions of these distances to the CM244 reference sequence for each tree). We compared these values across treatment groups for each tree, and found trending evidence of greater divergence from the LAI sequence in Pro among placebo-recipient sequences (p = 0.059) (S18 Table), a “vMismatch” result. We repeated this analysis using the median distance over the multiple sequences available from each subject (n = 3–14) rather than the *mindist* sequence distance (as previously described [8]), and found that the results were consistent. When applying a variant of this analysis using nucleotide sequence trees, we found a trend toward greater divergence of vaccine recipient Gag sequences to the LAI insert sequence (p = 0.072) and no significant or trending result in Pro.

### Insert-independent alignment score differentiates vaccine and placebo recipient breakthrough sequences

In addition to the phylogenetic analyses, we also evaluated whether the pairwise alignment similarity scores between all vaccine recipient sequences versus all placebo recipient sequences were different than what would be expected under the null hypothesis that the vaccine and placebo sequences came from the same distribution. We computed the Quasi-Earth Mover’s Distance (QEMD) using BLOSUM90 [32] pairwise alignment scores and compared it to the null distribution of the QEMD computed based on repeatedly permuting treatment assignments; this approach does not use the vaccine reference sequences and does not depend on an estimated phylogeny. While the PD analysis evaluates the across-group difference between within-group phylogenetic diversity, the QEMD analysis evaluates the between-group sequence variation. We found significantly less QEMD similarity in Gag sequences than would be expected under the null hypothesis (p = 0.041), consistent with the trend toward a sieve effect found via the phylogenetic analysis. We did not find significant evidence for QEMD dissimilarity for Pol (p = 0.16) or Env (p = 0.54).

### Variable loop lengths, numbers of cysteine residues, and frequencies of potential N-linked glycosylation sites are similar in vaccine and placebo recipient Envelope sequences

We compared variable loop lengths, numbers of cysteines, and frequencies of potential N-linked glycosylation sites (PNG sites) between vaccine and placebo recipient sequences. There were no significant differences in the *mindist* variable loop lengths in Env-gp120 between vaccine and placebo recipients in any of the five variable loops (Wilcoxon rank sum p-values > 0.20). Next, based on *mindist* sequences we compared the per-subject median number of cysteines in gp120 between the treatment groups; this analysis was motivated by the finding in Vax004 that 20% of trial participants had atypical cysteine variants [33]. The distributions of per-subject median numbers of cysteines were similar in the two treatment groups (average number of cysteines = 19.45 in vaccine recipients and 19.73 in placebo recipients).

Next, we identified PNG sites by searching each breakthrough sequence for tripeptide motifs of the form N-X-S or N-X-T, where X is any amino acid other than proline [34]. We compared the numbers of PNG sites between the treatment groups using the *mindist* sequences as well as with all sequences using the multiple outputation (MO) method [35], and found no significant or trending difference. We also tested for a difference across treatment groups in the distribution of PNG sites at each of the sites at which any subject had a PNG site, restricting to sites with sufficient diversity (defined as at least 4 sequences with a PNG site and at least 4 sequences without a PNG site). Of the 75 sites tested, only one had an unadjusted Fisher’s exact test p ≤ 0.05 (site 186s, p = 0.04). S8 Fig. shows the percentage of *mindist* sequences with a PNG site at each alignment position that had one or more PNG site.

## Discussion

Sieve analysis is a powerful tool for the evaluation of breakthrough infections in vaccine studies and complements related studies of immune correlates of infection risk among vaccine recipients. Sieve analysis leverages the randomized design of the trial by comparing features of infections across treatment groups, and can further suggest testable hypotheses about the targets of vaccine-induced immunity. By comparing HIV-1 breakthrough viruses that were isolated from vaccine and placebo recipients in the RV144 trial, we identified HIV-1 genetic determinants potentially associated with (unmeasured) vaccine-induced immune responses. Scanning across the HIV-1 proteome, we identified 19 signature sites in the vaccine proteins Env-gp120, Gag, and Pro that differed between the vaccine and placebo groups. In addition, we identified 37 signature sites in parts of the proteome that were not included in the vaccine. Four pairs of signature sites overlapped in different proteins across reading frames, resulting in a total of 52 unique sites across the proteome. Because our exploratory study was designed to identify all potential sieve effects, we reported all sites with unadjusted p-values below 0.05. Of the signature sites identified in vaccine immunogen regions, only Pol 51 passed the q-value ≤ 0.20 threshold.

Sieve analysis, by comparing breakthrough HIV-1 viruses across treatment groups, can test some of the specific hypotheses generated by correlates of risk analyses, such as the Haynes et al. [3] study that identified anti-V2 antibodies as a correlate of risk. For example, sieve analysis can test whether the breakthrough infections in the vaccine group are less viable targets for the vaccine-induced anti-V2 antibodies than the infections in the placebo group. The V1/V2 focused sieve analysis that identified V2 signature sites 169 and 181 [7] and follow-up studies [36] lent support to the hypothesis that anti-V1/V2 antibodies were involved in a mechanism of partial vaccine protection and that these sites are important for antibody binding. Confirming our previous study [7], the full proteome site-scanning analysis also identified signature sites 169 and 181, although unlike the previous V1V2-focused analysis results, the exploratory results reported here did not pass multiplicity correction, partly due to the much larger number of analyzed sites (8 compared to 248 in Env alone). We hope that additional follow-up experiments will further elucidate the role, if any, of the other newly-identified signatures in the partial protection conferred by the vaccine regimen.

Among all the signature sites, some are worth singling out because they were found by multiple methods and/or there is biological evidence supporting their potential vaccine-associated immunological relevance. In particular, Env 19, 169, 181, 317, 413 and 424 appear important because they are known antibody contact sites or belong to functionally important regions of the HIV envelope (S3 Table). The finding that Env signatures preferentially map to sites known to have a role in antibody neutralization or binding supports the hypothesis that the results are biologically meaningful. For example, the tridimensional structure of Env-gp120 showed that site 169 was in the vicinity of site 317. Env 169 is located at the crown of the V2 loop and was previously identified in the V1/V2-focused sieve analysis [7]. It is contained in a linear binding antibody epitope *hotspot* for RV144 vaccine-induced antibodies, and is a known contact site for neutralizing and binding antibodies. It is also part of a predicted HLA binding hotspot in the MN vaccine immunogen for both class I and class II alleles. Furthermore, this position is in the seventh position of a 9-mer that had significant treatment group differential binding escape versus the subtype B immunogen sequence (MN), a surprising discovery that motivated further analysis, leading to the finding that the differential vaccine efficacy at Env 169 was significantly associated with the class I HLA allele A*02 [10]. Env 317, identified by all three of the primary site-specific sieve methods, is in the core of the V3 loop and is part of the conserved co-receptor binding site. It is also known to be a contact site for neutralizing antibodies (*nAb-sites*), is part of an antibody *hotspot* defined using antigen microarrays [6], and is predicted to be on antibody interfaces using the *EPIMAP* method [7]. It exhibited a “vMismatch” sieve effect, in that there was greater divergence from the vaccine immunogen AA among the placebo recipient sequences than among the vaccine recipient sequences. It has been shown previously that mutations in V2 can interact with V3, and thereby have an impact on phenotypic changes such as co-receptor usage [37,38]. In addition, mutations in V3 can modulate the neutralization sensitivity of the conserved V2 epitope that is recognized by PG9/PG16-like antibodies. Interestingly, some antibodies isolated in RV144 vaccine recipients mapped to the same V2 region as PG9/PG16-like antibodies, implying that the mutations that we identified in V2 and V3 may have a synergistic impact on the neutralization sensitivity of breakthrough viruses [39].

Signature site Env 413 had the strongest influence on the variation of vaccine efficacy against HIV-1 as a function of genetic distance to CM244 computed using the Env-gp120 signature sites, and exhibited a vMismatch sieve effect. Env 413 is close to the CCR5 and 17b binding sites, and, together with signature site Env 424, it surrounds the binding motif RIKQ (residues 419–422). Most importantly, Env 413 was linked to the breadth of neutralizing antibody responses in a study that compared subjects with strong or weak neutralizing antibody responses [40]: an increase in breadth was associated with asparagine (N) at position 413. Here the consensus residue in CRF01_AE was T and the second most frequent residue found at that site was asparagine (N), which creates a site for potential N-linked glycosylation.

The signature sites identified with the site-scanning methods were characterized by greater amino acid covariation with other sites; there were more interactions with signature sites than at other sites as well as more interactions in gp120 (in the vaccine) than in gp41 (not in the vaccine). When across-protein interactions were considered, vaccine proteins showed greater connectedness: they covaried with more proteins. We conjecture that a vaccine-induced constraint at a highly connected site would have a greater impact on viral fitness than a change at a weakly connected site.

The differential vaccine efficacy (DVE) analysis allowed us not only to identify sites that differed between the infected vaccine and infected placebo groups but also to estimate the site-specific vaccine efficacy against viruses with a matched or mismatched residue to that present in a vaccine reference sequence at this given site. In Env, the DVE analysis identified six sites where estimated vaccine efficacy was increased to at least 43% (Env 19) and up to 85% (Env 317) against viruses with a specific residue at that site. Conversely, the vaccine efficacy was abolished with a different residue at these sites (point estimates ranging from less than zero percent to 17%). These results suggest that vaccine efficacy can essentially disappear with a single mutation. Better evaluating the VE/mutation relationship is critical for our understanding of vaccine immunity as it pertains to HIV-1. Knowing the genetic diversity of HIV-1, the disappearance of vaccine efficacy with a point mutation raises important questions as to the future efficacy of a successful vaccine. By analogy with drug-resistance mutations, we can envisage that the broad usage of a vaccine may lead to the increased frequency of mutations such as those that we found to be associated with null vaccine efficacy, and that such mutations would rapidly be selected in the population, hence reducing the vaccine efficacy. This also emphasizes that it may be necessary to have vaccines with multiple specificities in order to avoid the focusing of immune responses, which may lead to more rapid escape from vaccine-induced immunity, or that it may be important to include only essential protein sequences that cannot be mutated without impacting viral fitness.

The SmoothMarks sieve analysis did not find significant evidence that vaccine efficacy varied against HIV-1 genotypes with genotype defined by the genetic distance of breakthrough viruses to the CRF01_AE vaccine inserts. However, we found evidence of global T-cell based sieve effects relative to the CM244 and MN Env gp120 vaccine inserts using the EscapeCount T-cell sieve method. These results are surprising, since CD8+ T-cell response rates of RV144 vaccine recipients were low overall; depending on the sample time point and the assay that was used, 12–63% of vaccine recipients had a T-cell response to Env peptides, but these responses were predominantly CD4 responses [1,41,42]. One explanation is that the vaccine primed natural infection and an anamnestic response caused earlier escape in the vaccine group. We also note that these results were not found with the EpitopeDistance method. This may be due to differences in the definition of a T-cell sieve effect by the two methods. The global effects found here are also related to a V2-specific T-cell sieve analysis using the EscapeCount method that reported evidence of a T-cell sieve effect in the V2 region of the MN immunogen sequence [10].

By employing methods that incorporate T cell epitope binding predictions, our analysis indicates that vaccine-primed T cells and participants’ HLA alleles may have played a role either in early T cell escape or in modulating vaccine efficacy, even possibly at sites that are part of known antibody binding epitopes, such as the tips of the V2 and V3 loops and the CD4 binding site. Identification of potential sieve effects and vaccine-induced T cell epitopes motivates further study both experimentally and computationally, including, for example, testing for amino acid covariation within the PI9 peptide among infected participants (S6 Fig.). The putative effect within Env 299 PSNNTRTSI, along with those identified by the EscapeCount method in other 9-mers (S16 Table), generates hypotheses that can be further investigated computationally and tested experimentally with T cell assays. The suggestion of a trend toward greater phylogenetic diversity and divergence in Gag sequences for vaccine than placebo recipients could reflect the genetic effect of some T-cell mediated responses, although the signal is weak and there was no evidence of a sieve effect at predicted T-cell epitopes in Gag. In addition, our finding of 30 9-mers in Tat with a T cell sieve effect (passing the 20% q-value multiplicity adjustment) could possibly be explained by the fact that Tat is a viral regulatory factor for HIV gene expression and CD8 T-cell responses have been shown to select for viral escape variants in Tat during acute HIV and SIV infection [43].

It remains unclear whether the observed vaccine sieve effects are due to an acquisition barrier, reflecting the selection of viruses that managed to break through the protective effects of vaccination by the RV144 vaccine regimen, versus reflecting early post-acquisition immune pressures that affected within-host viral evolution after infection, or some combination of the two. We note that significant results found using methods that focus on T cell epitopes are not necessarily driven by T-cell pressure, and that signatures in Env may be driven by either T cell pressure, antibody pressure, or by a combination of the two.

Given that one could expect that sieve effects would be restricted to the proteins included in the RV144 vaccine, how can the 37 non-vaccine signature sites be interpreted? In addition, how can we explain that there are an approximately equal number of “vMismatch” and “vMatch” effects? Additionally, the physico-chemical property sieve effect sites tended to occur in non-vaccine proteins, as did all thirty-one sieve effect 9-mers that were significant after multiplicity correction. While some of these signature sites and 9-mers are false positives, others may be true effects. Certain non-immunogen sites/9-mers may be implicated because they are in linkage with other mutations in vaccine sequences; for instance such sites could act as compensatory mutations for vaccine-associated mutations that would be destabilizing. In addition, certain residues that are matched to the vaccine may be required for HIV-1 to be infectious/transmittable. Alternatively, some non-vaccine-immunogen signature sites/9-mers and vMismatch effects could reflect true effects of post-acquisition immune pressure that affect vaccine recipients more strongly or more rapidly than placebo recipients.

This comprehensive whole-genome sieve analysis generates additional testable hypotheses about the nature and mechanism of the vaccine’s partial efficacy, by identifying individual sites, peptide regions and proteins at which the genomic sequences significantly differed between vaccine and placebo recipients. By using a variety of methods, each tailored to detect different types of signals, we both increased the chance of finding differences and provided means for potentially explaining the differences. With additional support from independent analyses, such as viral inhibition experiments based on individual amino acid substitutions, a subset of the site-specific sieve effects identified here may prove to reflect vaccine immune pressure and thus be significant for future vaccine design and analysis. Directions for future research include experimental determination of vaccine-induced antibody binding in identified Env regions, evaluating functional consequences of the observed mutations, and further elucidating the extent to which differences in non-vaccine-immunogen regions of the breakthrough viruses could be directly attributed to vaccination, or indirectly attributed to constraints on the virus or to chance sampling variability.

The list of specific testable hypotheses includes evaluation of all of the signature sites for evidence of vaccine-induced immune pressure targeting each site. While in the absence of an additional efficacy trial it is not possible to directly evaluate the statement that “vaccine efficacy can essentially disappear with a single mutation”, it is possible to test the hypothesis that the vaccine-induced antibodies bind viruses differentially depending on individual mutations. This has been done for V2-targeting antibodies by Liao and colleagues [36] as well as for V3-targeting antibodies as reported by Susan Zolla-Pazner [11]) and could in principle be done for any of the (Env) signature sites. Neutralization assays could also be applied to assess differential neutralization, though the antibodies induced by RV144 appear to be non-neutralizing. More generally, effector function assays (e.g., ADCC) could be applied to assess differential functional responses.

After over 30 years of effort to develop an effective public health vaccine to prevent infection by HIV-1, the only vaccine to show statistically significant efficacy was the regimen used in the RV144 Thai trial. The borderline significant p-value of this result leaves open the possibility that the regimen had no overall efficacy. It would be possible for a vaccine with no overall vaccine efficacy to nevertheless have differential efficacy against different viruses. One example is a balancing effect, in which the vaccine has both protective and harmful effects, depending on the virus. Another possibility is that subjects who experience multiple exposures to HIV are protected against some viruses but ultimately become infected despite this partial protection (but later than they otherwise would have been); if the time delay is short (if the multiple exposures are close together in time), this could lead to negligible overall efficacy despite true acquisition sieve effects. In our view the strength of evidence for overall VE is increased by the findings of this study, for example the SmoothMarks analysis provided smaller p-values for overall VE by accounting for viral distances. However, experimental confirmation is still crucial, both because we are not able to prove that the observed sieve effects are acquisition effects and because of the expectation that many of these are false discoveries. Even if an effect is a true discovery (in that the treatment group differences are not due to chance variation), it may be an effect of vaccine-induced changes to the evolutionary course of the virus after infection (post-acquisition effects) rather than effects to prevent infection (acquisition effects). There were significant effects found in the sieve analysis of the Step 502 trial that are presumed to be post-acquisition effects because the vaccine immunogen had no Envelope component (and no evidence of antibody induction) and because the strongest effect was found at a known T-cell epitope and was strongest in subjects with the necessary HLA types to target that epitope [8,9]. In the absence of confirmatory studies, the signature site analysis would not increase the strength of evidence. However, the strength of evidence for overall efficacy has already been increased, in our view, by the experimental confirmation of RV144-induced V2-targeting mAbs that differentially bind depending on the amino acid at site Env 169, in conjunction with the significant correlation of vaccine efficacy with induction of V2-targeting Abs.

It has become clear that future vaccine studies should be designed for a more rapid iterative process, to maximize the information gleaned from each trial and ultimately to minimize the time to an effective global intervention strategy [44,45]. The correlates and sieve analyses of the RV144 trial demonstrate the importance of designing future trials with sufficient power to conduct such analyses. In particular, both types of analyses are improved by more precise resolution of the timing of HIV infection (e.g., accomplished through more frequent visits for diagnostic testing of HIV-1 infection that capture a sizable fraction of HIV infection events in the pre-seroconversion acute phase), which would allow use of statistical methods that can help tease apart acquisition sieve effects from post-acquisition differential within-host viral evolution [19].

## Materials and Methods

### Ethics statement

The protocol was approved by the Institutional Review Boards of the Ministry of Public Health, the Royal Thai Army, Mahidol University, and the US Army Medical Research and Materiel Command. Written informed consent was obtained from all participants.

### Study design

The study conduct and results have been published previously [1].

### RV144 vaccine regimen

The vaccine regimen was a combination of HIV-1 subtype B and HIV-1 CRF01_AE: the prime corresponded to HIV-1 Gag and Pro of subtype B LAI and the CRF01_AE HIV-1 gp120 (strain 92TH023) linked to the subtype B transmembrane domain of gp41 (strain LAI); the boost corresponded to the CRF01_AE HIV-1 gp120 strain CM244 with the subtype B HIV-1 gp120 strain MN. (CRF01_AE is subtype E in the HIV-1 Env.) We aligned these three sequences to the breakthrough sequences for analysis.

### Trial data

Of the 16,395 participants who entered the trial HIV-1 negative [the modified intention-to-treat (MITT) cohort], 125 acquired HIV-1 infection during the 3.5-year follow-up period. Of the 125 MITT infected subjects, we analyzed the subset of subjects who were infected by HIV-1 CRF01_AE viruses, for whom we have sequence data, and who were not infected by another trial participant (n = 109 subjects). Subjects infected with subtype B viruses (n = 11) were excluded because of the much larger HIV-1 genetic distances to the vaccine immunogen sequences compared to CRF01_AE, such that their inclusion would likely reduce statistical power of the sieve analysis by contributing genetic variation unrelated to a sieve effect. Four of the 125 infected subjects had no sequence data, three because the Sanger sequencing technology failed to deliver a result due to low HIV-1 viral load, and one because of drop-out. Finally, we excluded subject AA100 because this subject was the second to acquire HIV in the AA118/AA100 transmission pair; excluding AA100 avoids complications arising from the non-independence of these infections, and helps maintain plausibility of the ‘sieve conditions’, which are sufficient assumptions to justify interpretability of observed genotype-specific vaccine efficacies and infected-case sequence differences as prospective, per-contact estimates of genotype-specific vaccine efficacy [46]. The other linked transmission pair was subtype B, so both of those subjects were excluded on the grounds of their infecting subtype. Note that while most of the sieve analyses conditioned on infection (and therefore truly excluded from analysis all subjects other than the 109), the estimates of genotype-specific vaccine efficacy and differential vaccine efficacy, as well as the SmoothMarks multi-site acquisition sieve analyses, were time-to-infection analyses that included the entire MITT cohort (and right-censored, rather than truly excluded, the infected subjects outside of the 109). The vaccine efficacy to prevent acquisition of CRF01_AE HIV-1 (based on the MITT cohort and these 109 infections) was estimated to be 35.2% (95% CI 4.8% to 55.8%, score test p = 0.026).

### HIV-1 sequencing

The RV144 HIV-1 sequencing methodology has been published previously[8], and further information is provided in S1 Table and S2 Table. Sequences are available under GenBank accession numbers JX446645–JX448316.

### Representative *mindist* sequences

For each subject, we defined the *mindist* sequence to be the closest actual sequence to the consensus of that subject’s full-genome nucleotide sequences, as measured by the Tamura-Nei ‘93 (TN93) distance correction model [47]. A full description of our *mindist* selection process is presented in S1 Text. In short, subjects with a full-length nucleotide sequence that measured closest to their consensus with TN93 had that sequence used and translated for all *mindist* protein sequences. For subjects with only right-half or left-half sequences that measured closest to their consensus, the closest right- and left-half genomes were selected and thence translated into the appropriate *mindist* protein sequences. Ties were broken by (a) excluding sequences with the most ambiguous, incomplete or stop codons, (b) for right-half genomes, selecting the sequence with the shortest env distance, and (c) for left-half genomes, selecting the sequence with the shortest gag distance. Five ties remained after this procedure, which were broken randomly.

### Sieve analysis methods overview

Only the SmoothMarks and vaccine efficacy (VE) and differential VE (DVE) analyses utilize the entire “Modified Intent-to-Treat” (MITT) cohort of the RV144 trial, including subjects who did not become infected and subjects lost to followup. These methods are particularly well-suited to detect acquisition sieve effects, because under fairly general conditions these have been shown to be robust to post-hoc selection biases engendered by conditioning on infection. The other methods only include in the analysis infected subjects (who by definition are the only subjects with HIV-1 sequences available for analysis; see the Trial Data subsection of Methods for details), and (while generally applicable) are best-suited to evaluate post-acquisition sieve effects. Because of the six-monthly sampling scheme employed in the RV144 trial, the evaluated sequences are likely to have evolved between acquisition and sampling, and, of the methods applied here, only the SmoothMarks method attempts to recapitulate the genetic distance of the founder variant using missing-data methods. To our knowledge there is no existing method that can differentiate between acquisition and post-acquisition effects without incorporating longitudinal sequence data, which are not available for this trial.

The DVE method is designed to detect acquisition sieve effects of differential VE by Match vs. Mismatch of breakthrough sequences to the immunogen sequences, and the SmoothMarks method is designed to detect acquisition sieve effects of differential VE by continuous genetic distance of breakthrough sequences to the immunogen sequences. The other methods are designed to detect post-acquisition effects such as weighted mutation rates at single sites (GWJ uses a T-type test comparing AA substitution costs versus the vaccine immunogen, MBS uses a Bayesian model of post-acquisition sieve effects), incorporate multiple sequences per subject (SMMB and EGWJ), employ a phylogenetic model of sequence relatedness (divergence, diversity, PRIME, and FEL), evaluate codons for selection pressure (dN/dS and PRIME), and/or evaluate immunological hypotheses such as physico-chemical selection (PCP and PRIME), T cell escape (EscapeCount, EpitopeDistance, and PercentEpitopeMismatch), and antibody binding (signature site set enrichment, and SmoothMarks when applied to Ab site sets). The application of these varied methods provides a comprehensive exploratory evaluation of the effects of vaccination on breakthrough HIV-1 sequences.

### Phylogenetic diversity and divergence

Maximum likelihood phylogenetic trees were constructed (one tree per protein and per vaccine immunogen sequence) using PhyML (version 3.0) [48], using the HIV-between (HIVb) PAM substitution matrix[16], invariant sites, and four gamma-distributed rate categories. For each tree, the differential amino acid phylogenetic diversity (PD) [31] was defined as the difference in the total branch length of two subtrees (defined by holding the tree fixed and excluding a subset of leaves corresponding to one treatment group): the subtree excluding placebo recipients (retaining only sequences from vaccine recipients and the vaccine immunogen sequence); and the complementary subtree excluding vaccine recipients (but retaining the vaccine immunogen sequence). We estimated a null distribution by randomly permuting vaccine/placebo labels 10,000 times, and computed a (two-sided) p-value by comparing the observed difference in PDs to this null distribution.

The phylogenetic divergence analysis computed shortest-path distances between each subject’s sequence(s) and the vaccine immunogen sequence. For *mindist* analyses we used the trees computed for the PD analysis. For multiple-sequence-per-subject analyses, we constructed AA trees as above using all available sequences, and we constructed nucleotide trees using the GTR + I + G nucleotide substitution model using PhyML (version 3.0) [48] implemented in DIVEIN [49] (http://indra.mullins.microbiol.washington.edu/DIVEIN/diver.html). Tree-based distances were extracted from these trees using the NewickTermBranch algorithm (http://indra.mullins.microbiol.washington.edu/perlscript/docs/NewickTermBranch.html) and the ape package in the R computing language [50], and per-subject median distances were computed to each reference sequence. These distances were compared between the vaccine and placebo groups using a Wilcoxon rank sum test (one test per gene/reference combination).

### Quasi-Earth Mover’s Distance (QEMD) comparison of whole-gene pairwise alignment scores

We introduce a new application of the Earth-Mover’s Distance statistic to sieve analysis. The QEMD statistic equals the maximum over *W* of ∑(*S* * *W*) where *S* is the *n* by *m* matrix containing the pairwise alignment scores between vaccine and placebo *mindist* sequences, * denotes entrywise multiplication and *W* is an *n* by *m* weight matrix subject to the following constraints: *W* > 0, every row sums to 1/*n* and every column sums to 1/*m*. Note that in this case the QEMD statistic measures similarity (not distance). The QEMD hypothesis test reports two-sided “mid p-values” [51] based on random permutation of treatment assignments. We applied this approach with *n* and *m*, the total number of vaccine and placebo recipient sequences, respectively.

### SmoothMarks method

We used the *mindist* sequence as an approximation of the founder virus, and we computed distances between the immunogen sequences and the *mindist* sequence measured from blood samples drawn at or before the HIV diagnosis date. The SmoothMarks method [19,46] was used for estimation and testing of VE(*v*) over the range of distances *v* from 0 to 1, where the vaccine efficacy against HIV-1 with distance *v*, VE(*v*), is one minus the distance *v*-specific hazard ratio (vaccine/placebo) of HIV-1 infection multiplied by 100%. This method employs a missing-data framework to analyze VE(*v*) as a function of the “true distance” *v* between the transmitted founder sequence and the vaccine immunogen sequence. This can in principle improve the analysis over the other analysis methods that analyze the “observed distances” of available sequences that are measured weeks or months after infection; by not accounting for post-acquisition evolution these methods may obscure acquisition sieve effects. Since we do not have longitudinal sequence data, we are limited in our ability to estimate the transmitted founder sequence, so for the present analysis we defined “true” genetic distances as the HIVb-computed distance between the immunogen sequences and the *mindist* sequence measured from blood samples drawn at or before the HIV diagnosis date, where the 10% of infected subjects (11 of 109) with later sampled sequences were treated as missing data. See S2 Text for additional details about the method and its implementation.

### Vaccine efficacy (VE) and differential vaccine efficacy (DVE)

We assessed genotype-specific VE using the Cox proportional hazards model and score test as described by [52], and we assessed differential VE (DVE) by genotype using the same model, via the procedure described by Lunn and McNeil [14]. These were the primary analysis methods used previously [7]. Negative VE values are shown in symmetrized form (as the negative of the VE value calculated with vaccine and placebo groups interchanged).

### Other primary site-specific sieve analysis methods

Two additional primary site-scanning methods were used that assess at each site whether the amino acid distances to a reference immunogen at that site differ for vaccine compared to placebo recipient sequences: a nonparametric weighted distance comparison test (GWJ) [17], and a model-based method (MBS) [18]. Both of these methods were based on the *mindist* amino acid sequences. Code for these methods was published previously [7].

### Physico-chemical property (PCP) analysis

We introduce the PCP analysis method, which compares counts of each of the ten Taylor properties [21], and five z-scale components [22–24] across treatment groups using parametric two-sample pooled-variance two-sided t-tests. The analysis can apply to individual sites or to arbitrary site sets (we evaluated 3-mers and 9-mers), in the latter case by summing counts over sites. The resulting p-values are then Bonferroni-corrected across the properties for each of the two property scales at each site (for all k-mers overlapping that site, separately for each value of k).

### Peptide microarray *hotspots*

Peptide microarrays designed to cover the entire gp160 consensus sequences for HIV-1 Group M, subtypes A, B, C, D, CRF01_AE and CRF02_AG for a total of 1423 peptides (15-mers overlapping by 12 amino acids) were used to detect reactive regions for RV144 vaccine recipients. Using the analysis method of Imholte et al. [53], four dominant responses were detected in the C1, V2, V3 and C5 regions of gp120 [6]. These sites are listed in S3 Dataset.

### Antibody *contactsites*

This is a set of known and published monoclonal antibody contact sites provided by Ivelin Georgiev, Peter Kwong, Robin Stanfield, and Ian Wilson [54,55]. They are listed in S3 Dataset.

### Neutralizing antibody *nAb-sites*

These are the sites identified as relevant to the neutralization activity of known neutralizing antibodies in Wei et al. (2003) [54], Moore et al. (2009) [55], and Tomaras et al. (2011) [56]. They are listed in S3 Dataset.

### Antibody *contactsites-augmented*

These are the union of sites in *contactsites* and *nAb-sites*.

### *EPIMAP* predicted antibody contact sites

Potential antibody contact “patches” were calculated by the method described previously [7], but considering all of the Env protein rather than only the V1/V2 region. Sites were sorted by frequency of inclusion in these patches (by the mean of their frequency of inclusion in patches versus the 92TH023 sequence and the maximum of their frequencies of inclusion in patches versus the CM244 and MN sequences), as shown in S9 Fig.. The same threshold used previously [7] was used to select top-scoring sites. The *EPIMAP* site set contains the 71 sites that passed this threshold, 38 of which overlap vaccine sequence sites. They are listed in S3 Dataset.

### HLA-associated Sites: *HLA-I* and *HLA-II* epitope enrichment sites

We defined two sets of sites where selective pressure by T cells was putatively highest. These analyses considered only the vaccine immunogen sequences (and the HLA types of the subjects), and were conducted blinded to subject treatment assignment. They are listed in S4 Dataset.

1. *MHC-I* predicted epitopes: We predicted vaccine epitope hotspots based on either a strong (IC50<50nM) or weak (IC50<500nM) predicted MHC-I binding threshold. First, the HLA binding affinity was predicted using the adaptive double threading (ADT) method [28] for each 9-mer in the vaccine immunogen sequence and each class I HLA allele expressed by any of the 109 relevant HIV-1-infected trial participants. Every 9-mer with IC50 less than the strong (or weak) binding threshold was considered a potential vaccine epitope for each person who expressed the restricting HLA allele. The total number of potential epitopes overlapping each site, considering all 109 infected trial participants, was counted. If this number was significantly greater than the number of epitopes that would be expected to overlap the site by random chance alone, then the site was considered an epitope “hotspot.” Significance was determined by counting the total number of predicted epitopes, summing across all 4 HLA-A and HLA-B alleles for each and every participant and across the entire protein. The null hypothesis is that these putative epitopes are uniformly distributed with a per site probability equal to the number of predicted epitopes divided by the number of all potential 9-mer epitopes (adjusted for the fact that each site is contained within 9 potential 9-mer epitopes). A two-sided binomial test was used to determine if the number of predicted epitopes overlapping a single site exceeded that expected under the null hypothesis. The predicted T cell epitope set was defined using either **S**trong (<50nM) or **W**eak (<500nM) binding thresholds, where sites significant using **B**oth binding thresholds are marked with a **B** (S, W, B notation is reported in S3 and S4 Table).
2. *MHC-II* predicted epitopes. Similar to the prediction of Class I epitopes, we predicted Class II epitopes by predicting the binding affinity of MHC II with vaccine immunogen sequence 15-mers (using the NetMHCIIpan predictor [57]). The sites encompassed by 15-mers that bind to the MHC II alleles of study participants were included in this set. Again, we defined two versions of the set based on “strong binders” and on “weak binders,” as defined above.

## Supporting Information

S1 Fig Distributions of vaccine and placebo Env sequence AA distances to the 92TH023 and CM244 vaccine sequences. Distances for the *contactsites, constactsites-augmented, hotspots, EPIMAP*, and *all* Env-gp120 site sets were computed based on *mindist* amino acid sequences computed with the HIVb PAM substitution matrix[16]. Box plots show the 25^th^ percentile (lower edge of the box), 50^th^ percentile (horizontal line in the box), and 75^th^ percentile (upper edge of the box).
(TIF)

S2 Fig Estimated vaccine efficacy as a function of distance to Ab-relevant regions of the CRF01_AE vaccine sequences. SmoothMarks estimates of vaccine efficacy (VE) against acquisition with an HIV-1 CRF01_AE virus with genetic distance *v* from the 92TH023 or CM244 vaccine sequences, with 95% confidence intervals, using Env *mindist* amino acid sequences and computed with the HIVb PAM substitution matrix[11]. For each panel, the first p-value is for testing whether there is any VE against any virus genotype, and the second p-value is for testing whether VE varies with the distance *v*. With 176 residues in *contactsites*, distances 0.08 to 0.25 correspond to 13–39 amino acid mismatches; with 194 residues in *contactsites-augmented*, distances 0.10 to 0.27 correspond to 14–45 mismatches; with 196 residues in *hotspots*, distances 0.03—0.17 correspond to 7—26 mismatches; with 69 residues in *EPIMAP*, distances 0.03 to 0.29 correspond to 4—17 mismatches; and with 425 residues in *all*, distances 0.08—0.19 correspond to 29—67 mismatches.
(TIF)

S3 Fig Signature-Sites Env sequence AA distances to the 92TH023 and CM244 vaccine sequences and estimated vaccine efficacy. (A) and (B) show distributions of amino acid *signature-sites* distances based on the *mindist* sequences and the HIVb PAM substitution matrix[16]. Box plots show the 25^th^ percentile (lower edge of the box), 50^th^ percentile (horizontal line in the box), and 75^th^ percentile (upper edge of the box). Panels (C) and (D) show SmoothMarks estimates of vaccine efficacy (VE) against acquisition with an HIV-1 CRF01_AE virus with *signature-sites* distance *v* from the 92TH023 or CM244 vaccine sequences with 95% confidence intervals. With 10 residues in *signature-sites*, distances 0 to 0.66 correspond to 0—6 amino acid mismatches.
(EPS)

S4 Fig Influence analysis of Env signature sites on vaccine efficacy. The upper panel shows the AA patterns for the 10 Env signature sites for each of the 109 infected subjects. The subjects’ AAs are shown in columns, sorted by genetic distance measured over these 10 sites. Vaccine-recipient columns are shown in orange, placebo-recipient columns in yellow. The lower panel shows the SmoothMarks-estimated vaccine efficacy (VE) curve as a function of the signature sites distances to the CM244 vaccine sequence for each subject. Note that the distances have ties and are not equally spaced; see S3 Fig., panel D, for an undistorted representation. The initial increase in the VE curve must occur due to a greater proportion of placebo than vaccine recipient mismatches versus CM244 at the 5 “vMatch” sieve effect sites and/or to a greater proportion of vaccine than placebo group mismatches at the 5 “vMismatch” sieve effect sites. During the sharp period of initial increase of VE in the distance region 0.066 to 0.166, the “vMismatch” signature site Env 413 has dominant influence, with 5 more vaccine than placebo recipients having a mismatched residue (10 vs. 5), and no other sites had a differential number of mismatches for vaccine versus placebo recipients. Conversely, the declining VE curve in the distance region 0.28 to 0.53 must occur due to a greater proportion of vaccine than placebo group mismatches at the 5 “vMatch” sieve effect sites and/or a greater proportion of placebo than vaccine group mismatches at the 5 “vMismatch” sieve effect sites. Env 413, Env 268, and Env 317 have the heaviest influence to create the declining VE curve in this region, with 11 (26 vs. 15), 10 (15 vs. 5), and 9 (9 vs. 0) more placebo than vaccine recipients having a mismatched residue. Six of the other ten signature sites (4 “vMatch”, 2 “vMismatch”) also influenced the declining curve in this region to a lesser extent (“vMatch”: Env 424 with 5 more vaccine than placebo recipients having a mismatched residue; Env 169, Env 353, and Env 369 each with 3 more; “vMismatch”: Env 481 with 4 more placebo than vaccine recipients having a mismatched residue and Env 379 with 1 more). Overall, these results suggest that, among the signature sites, the “vMismatch” signature sites Env 268, Env 317, and Env 413 have the greatest influence on vaccine efficacy.
(TIF)

S5 Fig Mapping of signature sites and sites under selection on an Env trimer structure. The panels are rotated 90° compared to Fig. 6. (a–e) Panels correspond to the five physico-chemical properties analyzed for evidence of positive selection based on dN/dS: a) chemical composition, b) polarity, c) volume, d) iso-electric point or e) hydropathy [25]. Signature sites identified in Env-gp120 are colored in green, and sites that were under selection are colored from pink to red (corresponding p-values from 0.05 to < 0.0001). (f) Visualization of the major sites of vulnerability on the HIV-1 Env.
(TIF)

S6 Fig Putative T-cell driven sieve effects in HIV envelope. For 9-mer “PSNNTRTSI” (PI9, HXB2 start position 299), there was a greater number of HLA binding escapes in vaccine versus placebo recipients (p = 0.0084, A). (B) Box plots indicate HLA binding affinities of the “breakthrough” 9-mers aligned with PI9 isolated from placebo and vaccine recipients (red and blue filled circles). Plots include only those participants who express one of the HLA alleles that bind the vaccine 9-mer with high affinity (horizontal lines). (C) Within the 9-mer, amino acid substitutions relative to the vaccine underlie the shifts in “breakthrough” binding affinity; thus sieve effects appear to be driven by different substitutions in the vaccine and placebo recipients’ sequences. Identification of potential sieve effects and vaccine-induced T cell epitopes motivates further study both experimentally and computationally, including, for example, testing for amino acid covariation within PI9 among infected participants. (D) Positions on the grid indicate the quantity of scaled mutual information (M*, color scale) shared by the amino acid variation at a pair of sites [58] and the associated unadjusted p-value (white annotation).
(TIF)

S7 Fig Distributions of *mindist* AA tree divergences. Box plots show the 25^th^ percentile (lower edge of the box), 50^th^ percentile (horizontal line in the box), and 75^th^ percentile (upper edge of the box).
(EPS)

S8 Fig Frequencies of potential N-linked glycosylation sites (PNG sites) in gp120 for vaccine versus placebo sequences. Frequencies of PNG sites at all gp120 sites (excluding sites at which the multiple alignment was poorly resolved) for *mindist* amino acid sequences. Blue bars above the horizontal line are for placebo sequences and red bars below the line are for vaccine sequences. There was no evidence of differences in PNG frequencies at any sites between vaccine and placebo sequences.
(TIF)

S9 Fig Frequency of sites in potential antibody contact patches. The EPIMAP method used by[7] was applied to estimate potential antibody contact patches of Env sites. Sites were sorted by frequency of inclusion in these patches, showing that some sites are more likely to be on the surface of the Env protein and other sites are more likely to be buried and inaccessible to antibodies.
(TIFF)

S1 Table Numbers of HIV-1 protein sequences measured from the n = 109 HIV-1 CRF01_AE infected subjects in the RV144 trial: Vaccine immunogen proteins.
(DOC)

S2 Table Numbers of HIV-1 protein sequences measured from the n = 109 HIV-1 CRF01_AE infected subjects in the RV144 trial: Non-vaccine immunogen proteins.
(DOC)

S3 Table Biological annotation of the identified signature sites in vaccine proteins.
(DOC)

S4 Table Biological annotation of the identified signature sites in non-vaccine proteins.
(DOC)

S5 Table Significant 9-mer sieve effects in vaccine proteins (by KmerScan).
(DOC)

S6 Table Significant 9-mer sieve effects in non-vaccine proteins (by KmerScan).
(DOC)

S7 Table Physico-chemical Properties (PCP) site-scanning results in vaccine proteins.
(DOC)

S8 Table Physico-chemical Properties (PCP) site-scanning results in non-vaccine proteins.
(DOC)

S9 Table Physico-chemical Properties (PCP) 3-mer results in vaccine proteins.
(DOC)

S10 Table Physico-chemical Properties (PCP) 3-mer results in non-vaccine proteins.
(DOC)

S11 Table Physico-chemical Properties (PCP) 9-mer results in vaccine proteins.
(DOC)

S12 Table Physico-chemical Properties (PCP) 9-mer results in non-vaccine proteins.
(DOC)

S13 Table dN/dS by Physico-chemical Property (PCP) site scanning results.
(DOC)

S14 Table Summary of analyses of predicted T cell epitope sieve effects in vaccine proteins.
(DOC)

S15 Table Summary of analyses of predicted T cell epitope sieve effects in non-vaccine proteins.
(DOC)

S16 Table Significant EscapeCount 9-mer and 15-mer results.
(DOC)

S17 Table Comparison of phylogenetic diversity (PD) between vaccine and placebo sequences.
(DOC)

S18 Table Comparison of phylogenetic divergence between vaccine and placebo sequences.
(DOC)

S1 Text Sequence data construction and processing.
(DOCX)

S2 Text The SmoothMarks method.
(DOCX)

S3 Text HLA-dependent covariation analysis.
(DOCX)

S4 Text The EscapeCount method.
(DOCX)

S5 Text The PercentEpitopeMismatch method.
(DOCX)

S6 Text The EpitopeDistance method.
(DOCX)

S7 Text Supplementary references.
(DOCX)

S1 Dataset Exhaustive site-scanning and kmer-scanning results table, in a .zip file.
(ZIP)

S2 Dataset HLA-dependent site covariation table, in a .zip file.
(ZIP)

S3 Dataset Site masks and filters table, in a .zip file.
(ZIP)

S4 Dataset T cell epitopes per person (TCEPP) results table, in a .zip file.
(ZIP)
