# Correction: Comprehensive Sieve Analysis of Breakthrough HIV-1 Sequences in the RV144 Vaccine Efficacy Trial

**DOI:** 10.1371/journal.pcbi.1004733

**Published:** 2016-01-26

**Authors:** The PLOS Computational Biology Staff

Gustavo H. Kijak should be included as a co-author of this paper.

The author’s affiliation is as follows: US Military HIV Research Program, Silver Spring, Maryland, United States of America

The author list should read as follows:

Paul T. Edlefsen, Morgane Rolland, Tomer Hertz, Sodsai Tovanabutra, Andrew J. Gartland, Allan C. deCamp, Craig A. Magaret, Hasan Ahmed, Raphael Gottardo, Michal Juraska, Connor McCoy, Brendan B. Larsen, Eric Sanders-Buell, Chris Carrico, Sergey Menis, Gustavo H. Kijak, Meera Bose, RV144 Sequencing Team, Miguel A. Arroyo, Robert J. O’Connell, Sorachai Nitayaphan, Punnee Pitisuttithum, Jaranit Kaewkungwal, Supachai Rerks-Ngarm, Merlin L. Robb, Tatsiana Kirys, Ivelin S. Georgiev, Peter D. Kwong, Konrad Scheffler, Sergei L. Kosakovsky Pond, Jonathan M. Carlson, Nelson L. Michael, William R. Schief, James I. Mullins, Jerome H. Kim, Peter B. Gilbert

The Author Contributions section should read as follows:

Conceived and designed the experiments: MR ST ESB GHK NLM JHK JIM. Performed the experiments: GHK RJO SN PP JK SRN MLR NLM JHK BBL SN MB MAA. Analyzed the data: PTE MR TH GHK PBG BBL SLKP KS WD BSM AJG ACd CAM HA MJ JMC CM RG. Contributed reagents/materials/analysis tools: CC SM GHK WRS JSM ISG TK PDK RG. Wrote the paper: PTE MR TH ST PBG.
