# Supplementary figures and images for: Comprehensive Sieve Analysis of Breakthrough HIV-1 Sequences in the RV144 Vaccine Efficacy Trial
Source: PLoS Comput Biol. 2015 Feb 3;11(2):e1003973. doi: 10.1371/journal.pcbi.1003973 (PMC4315437; doi:10.1371/journal.pcbi.1003973)

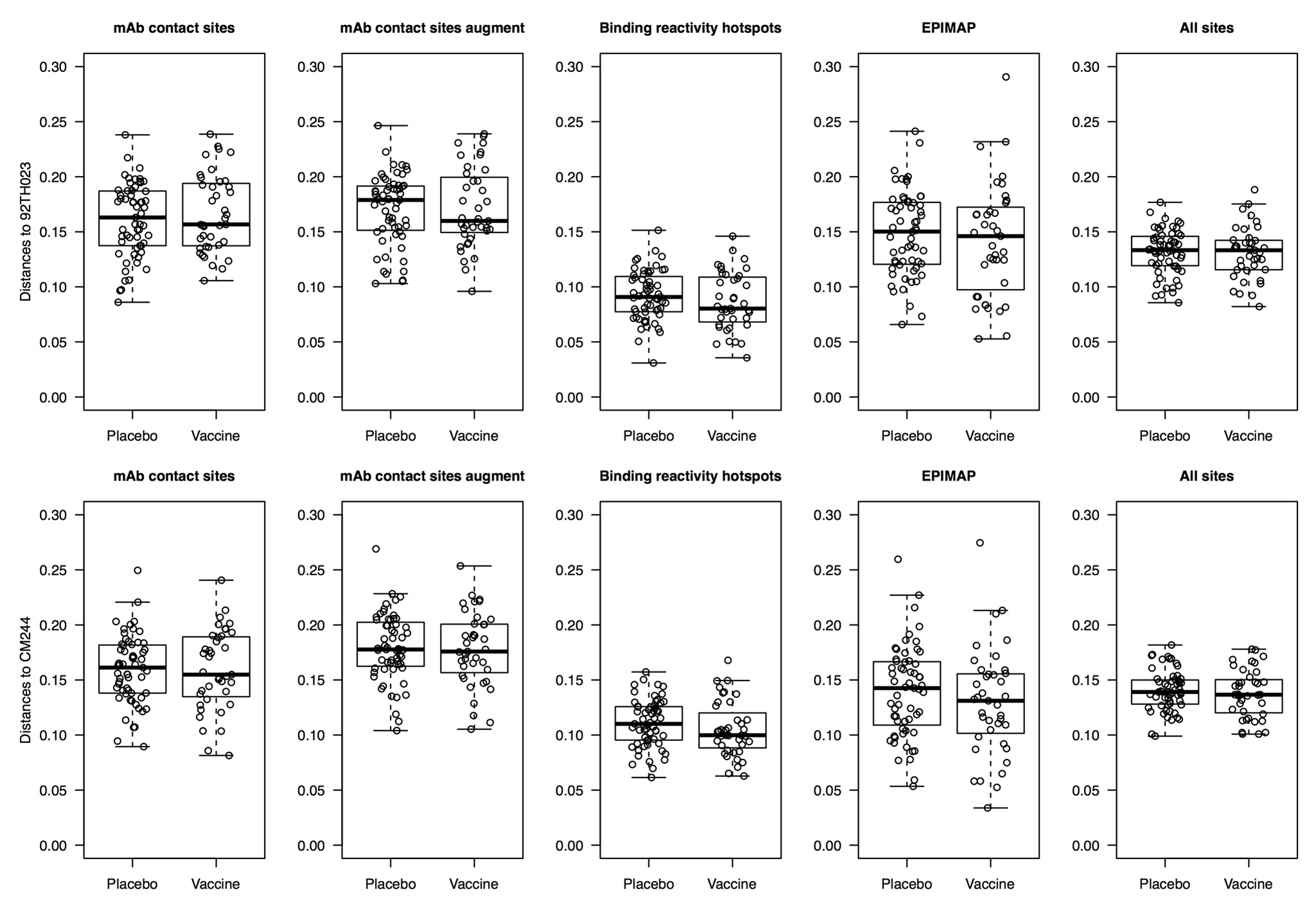

Supplement: S1 Fig — Distances for the contactsites, constactsites-augmented, hotspots, EPIMAP, and all Env-gp120 site sets were computed based on mindist amino acid sequences computed with the HIVb PAM substitution matrix[16]. Box plots show the 25th percentile (lower edge of the box), 50th percentile (horizontal line in the box), and 75th percentile (upper edge of the box). (TIF) [file pcbi.1003973.s001.tif]

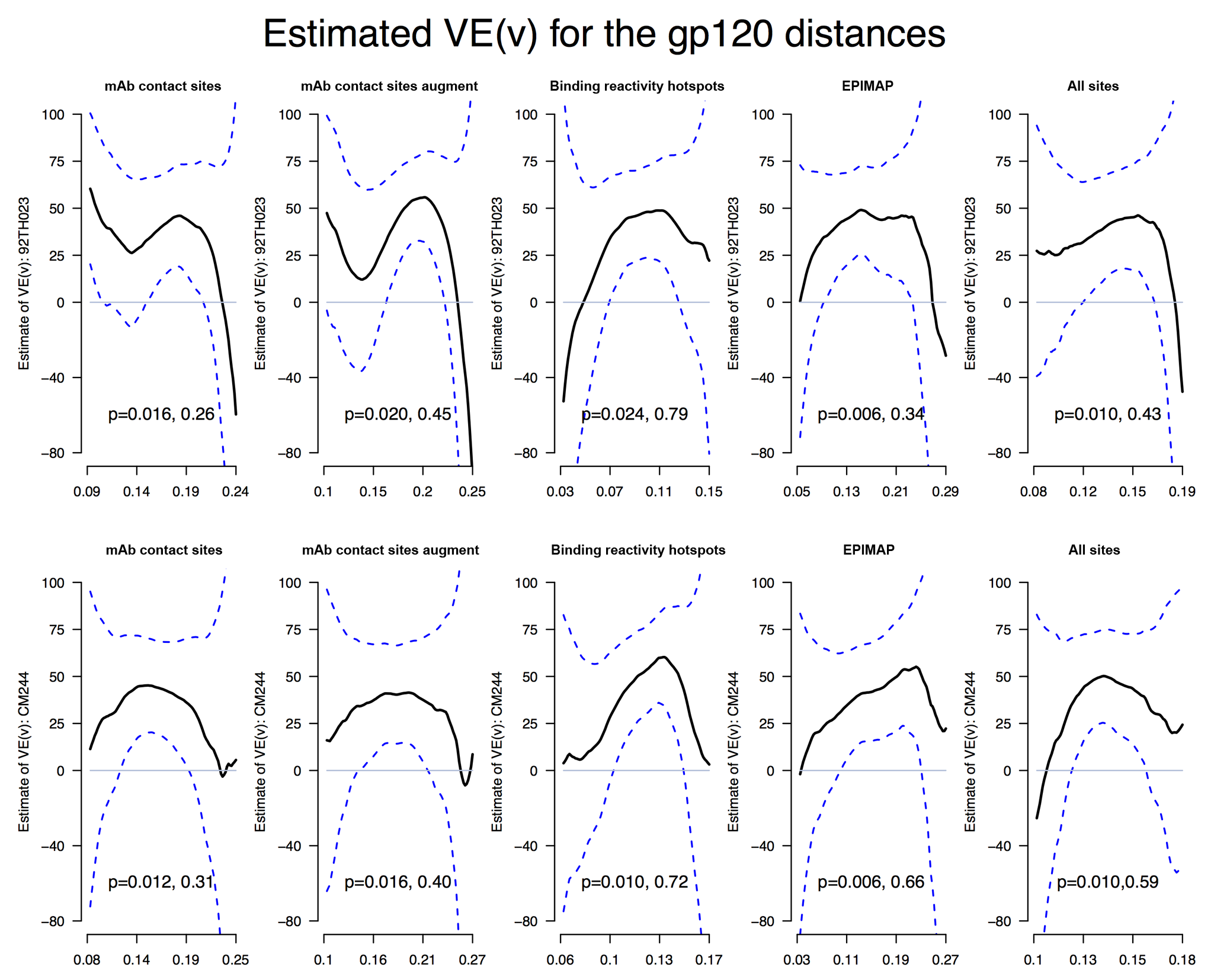

Supplement: S2 Fig — SmoothMarks estimates of vaccine efficacy (VE) against acquisition with an HIV-1 CRF01_AE virus with genetic distance v from the 92TH023 or CM244 vaccine sequences, with 95% confidence intervals, using Env mindist amino acid sequences and computed with the HIVb PAM substitution matrix[11]. For each panel, the first p-value is for testing whether there is any VE against any virus genotype, and the second p-value is for testing whether VE varies with the distance v. With 176 residues in contactsites, distances 0.08 to 0.25 correspond to 13–39 amino acid mismatches; with 194 residues in contactsites-augmented, distances 0.10 to 0.27 correspond to 14–45 mismatches; with 196 residues in hotspots, distances 0.03—0.17 correspond to 7—26 mismatches; with 69 residues in EPIMAP, distances 0.03 to 0.29 correspond to 4—17 mismatches; and with 425 residues in all, distances 0.08—0.19 correspond to 29—67 mismatches. (TIF) [file pcbi.1003973.s002.tif]

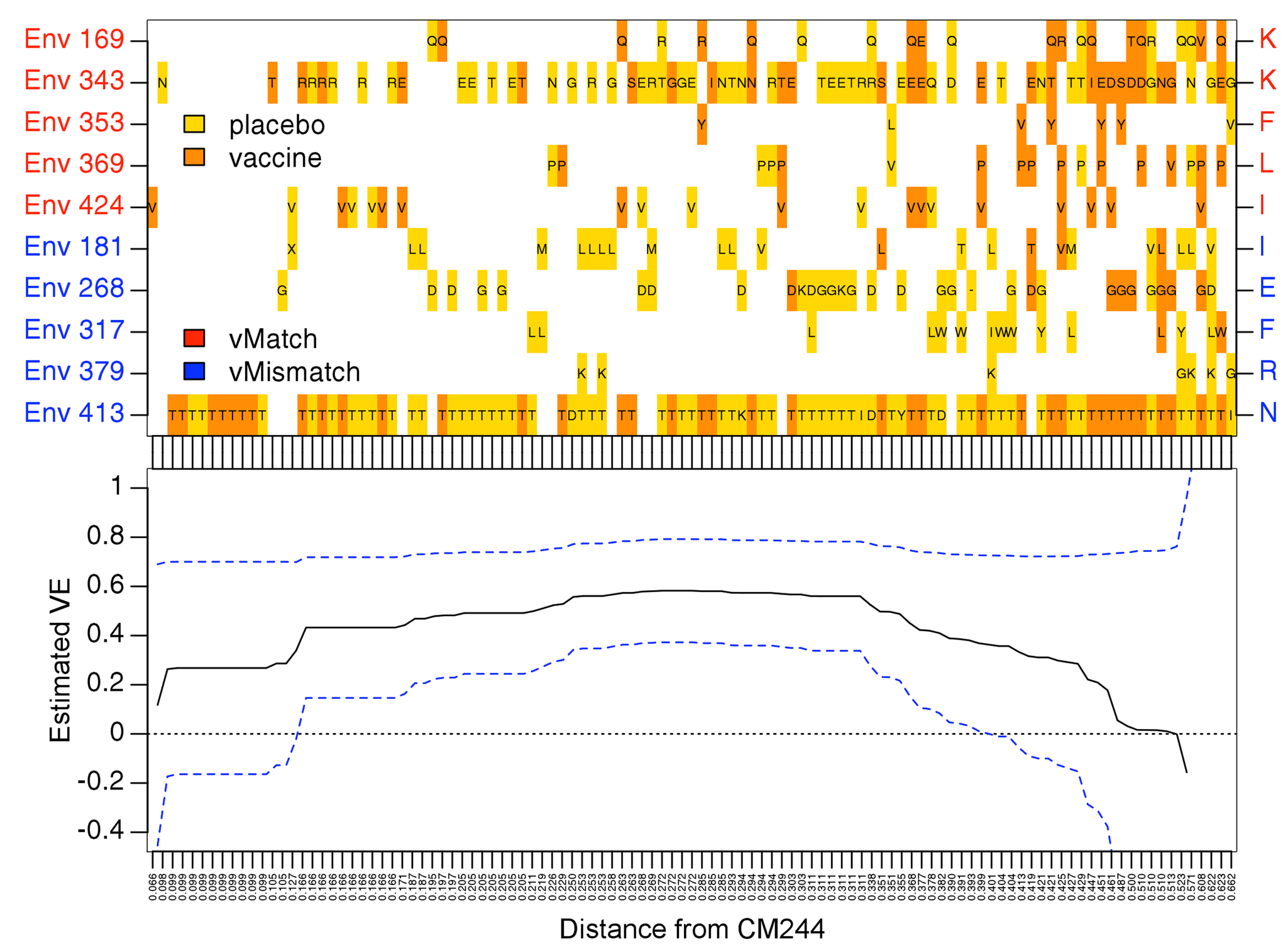

Supplement: S4 Fig — The upper panel shows the AA patterns for the 10 Env signature sites for each of the 109 infected subjects. The subjects’ AAs are shown in columns, sorted by genetic distance measured over these 10 sites. Vaccine-recipient columns are shown in orange, placebo-recipient columns in yellow. The lower panel shows the SmoothMarks-estimated vaccine efficacy (VE) curve as a function of the signature sites distances to the CM244 vaccine sequence for each subject. Note that the distances have ties and are not equally spaced; see S3 Fig., panel D, for an undistorted representation. The initial increase in the VE curve must occur due to a greater proportion of placebo than vaccine recipient mismatches versus CM244 at the 5 “vMatch” sieve effect sites and/or to a greater proportion of vaccine than placebo group mismatches at the 5 “vMismatch” sieve effect sites. During the sharp period of initial increase of VE in the distance region 0.066 to 0.166, the “vMismatch” signature site Env 413 has dominant influence, with 5 more vaccine than placebo recipients having a mismatched residue (10 vs. 5), and no other sites had a differential number of mismatches for vaccine versus placebo recipients. Conversely, the declining VE curve in the distance region 0.28 to 0.53 must occur due to a greater proportion of vaccine than placebo group mismatches at the 5 “vMatch” sieve effect sites and/or a greater proportion of placebo than vaccine group mismatches at the 5 “vMismatch” sieve effect sites. Env 413, Env 268, and Env 317 have the heaviest influence to create the declining VE curve in this region, with 11 (26 vs. 15), 10 (15 vs. 5), and 9 (9 vs. 0) more placebo than vaccine recipients having a mismatched residue. Six of the other ten signature sites (4 “vMatch”, 2 “vMismatch”) also influenced the declining curve in this region to a lesser extent (“vMatch”: Env 424 with 5 more vaccine than placebo recipients having a mismatched residue; Env 169, Env 353, and Env 369 each with 3 more; “vMis [file pcbi.1003973.s004.tif]

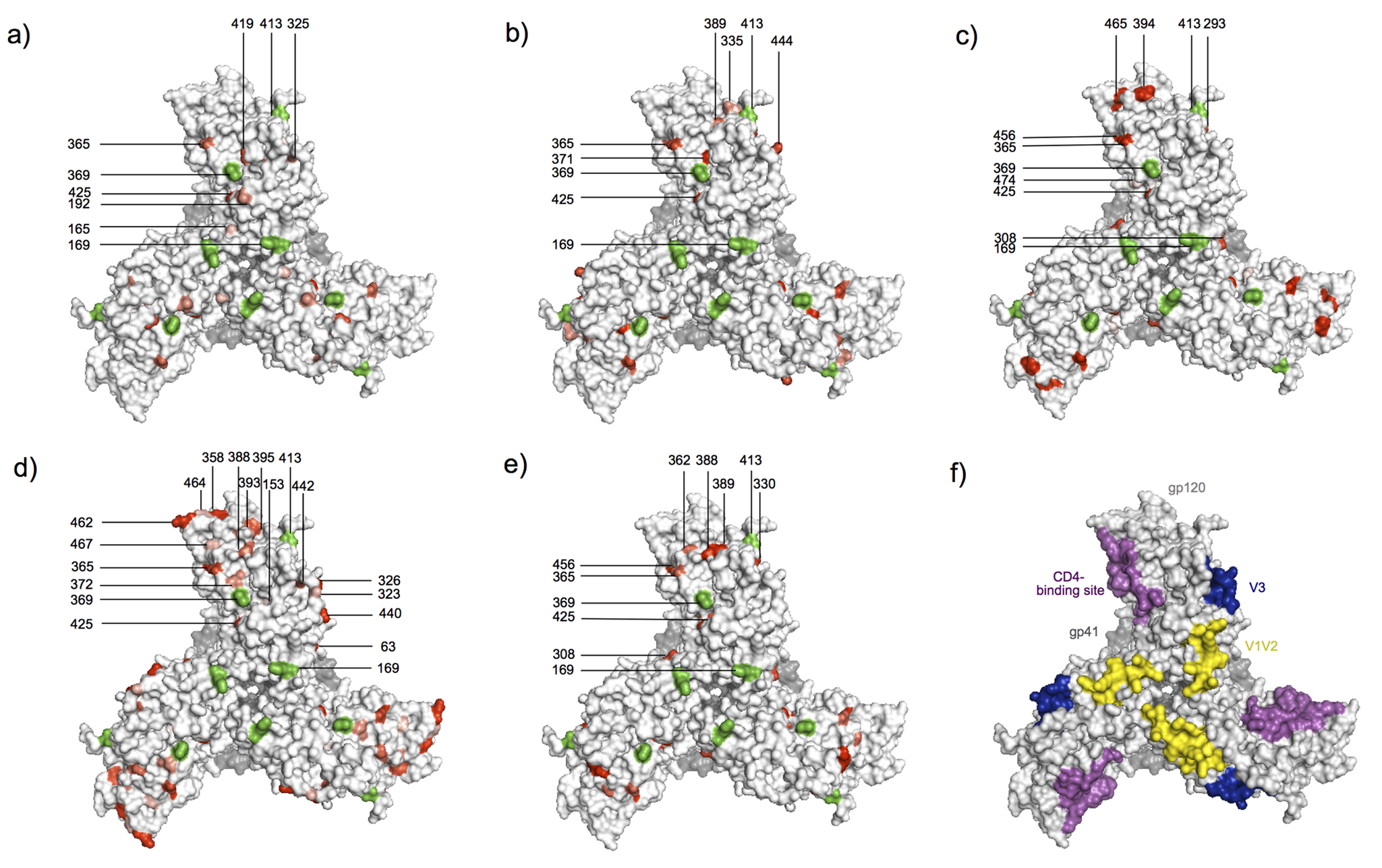

Supplement: S5 Fig — The panels are rotated 90° compared to Fig. 6. (a–e) Panels correspond to the five physico-chemical properties analyzed for evidence of positive selection based on dN/dS: a) chemical composition, b) polarity, c) volume, d) iso-electric point or e) hydropathy [25]. Signature sites identified in Env-gp120 are colored in green, and sites that were under selection are colored from pink to red (corresponding p-values from 0.05 to < 0.0001). (f) Visualization of the major sites of vulnerability on the HIV-1 Env. (TIF) [file pcbi.1003973.s005.tif]

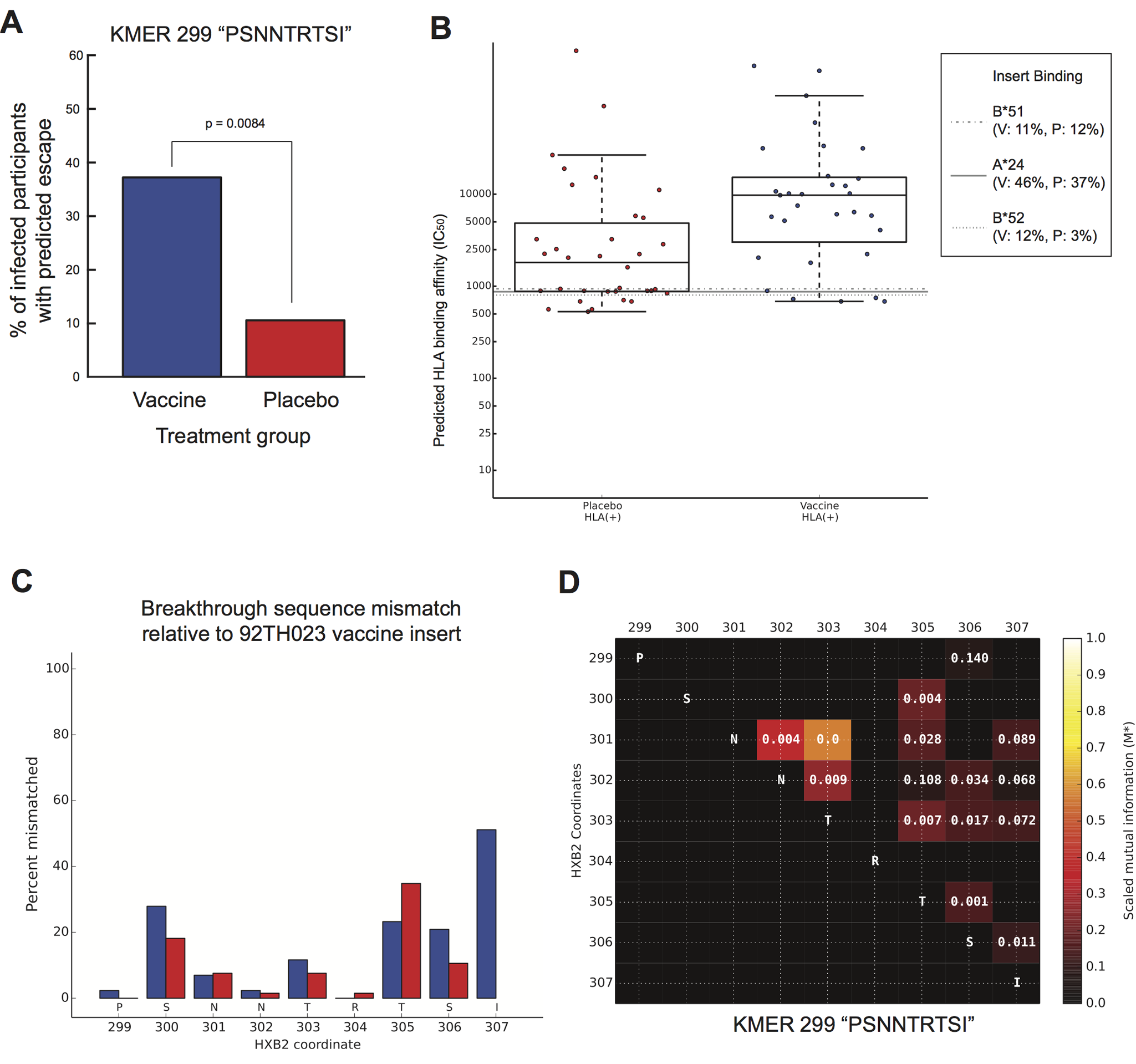

Supplement: S6 Fig — For 9-mer “PSNNTRTSI” (PI9, HXB2 start position 299), there was a greater number of HLA binding escapes in vaccine versus placebo recipients (p = 0.0084, A). (B) Box plots indicate HLA binding affinities of the “breakthrough” 9-mers aligned with PI9 isolated from placebo and vaccine recipients (red and blue filled circles). Plots include only those participants who express one of the HLA alleles that bind the vaccine 9-mer with high affinity (horizontal lines). (C) Within the 9-mer, amino acid substitutions relative to the vaccine underlie the shifts in “breakthrough” binding affinity; thus sieve effects appear to be driven by different substitutions in the vaccine and placebo recipients’ sequences. Identification of potential sieve effects and vaccine-induced T cell epitopes motivates further study both experimentally and computationally, including, for example, testing for amino acid covariation within PI9 among infected participants. (D) Positions on the grid indicate the quantity of scaled mutual information (M*, color scale) shared by the amino acid variation at a pair of sites [58] and the associated unadjusted p-value (white annotation). (TIF) [file pcbi.1003973.s006.tif]

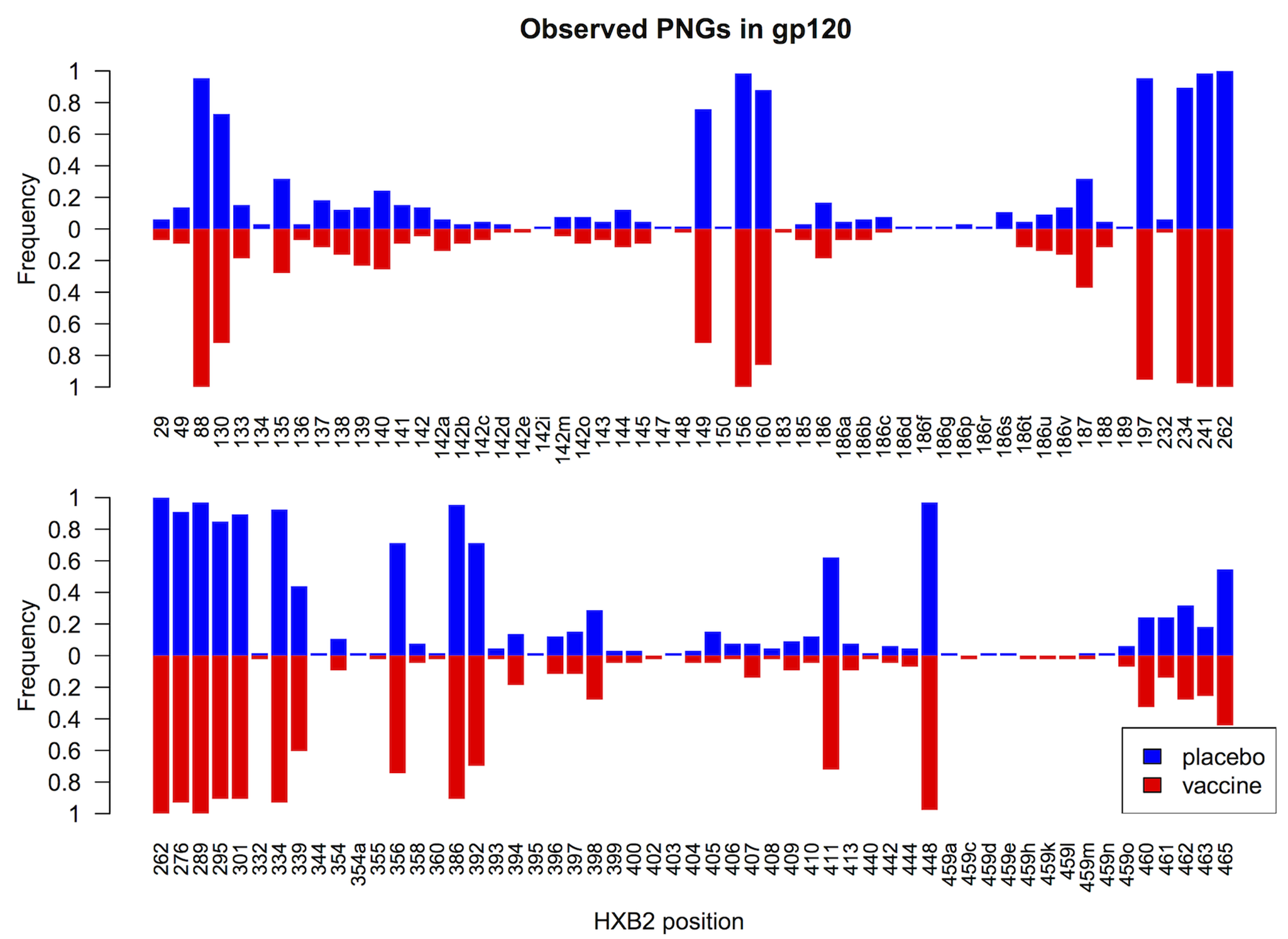

Supplement: S8 Fig — Frequencies of PNG sites at all gp120 sites (excluding sites at which the multiple alignment was poorly resolved) for mindist amino acid sequences. Blue bars above the horizontal line are for placebo sequences and red bars below the line are for vaccine sequences. There was no evidence of differences in PNG frequencies at any sites between vaccine and placebo sequences. (TIF) [file pcbi.1003973.s008.tif]

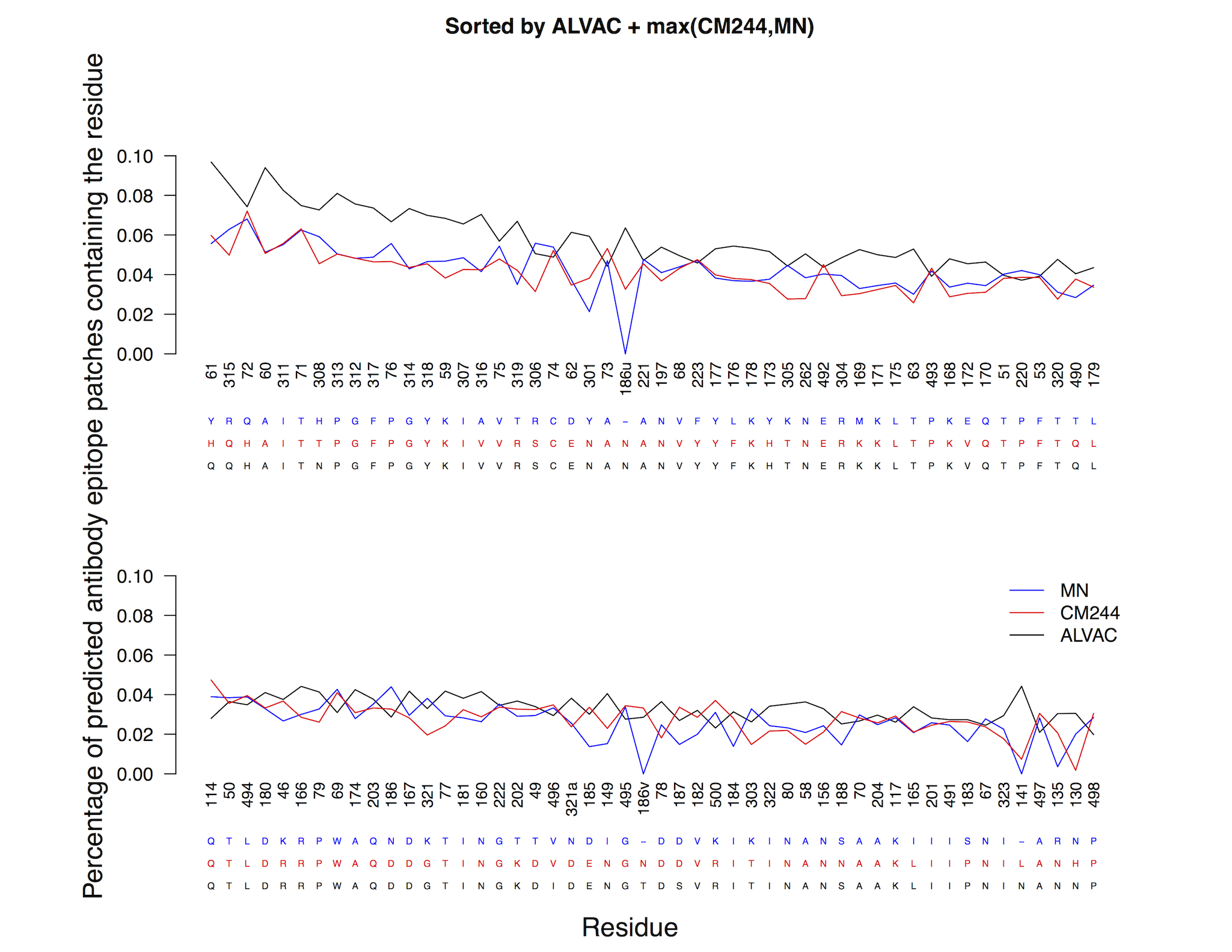

Supplement: S9 Fig — The EPIMAP method used by[7] was applied to estimate potential antibody contact patches of Env sites. Sites were sorted by frequency of inclusion in these patches, showing that some sites are more likely to be on the surface of the Env protein and other sites are more likely to be buried and inaccessible to antibodies. (TIFF) [file pcbi.1003973.s009.tiff]
