# Supplementary material for: Comprehensive Sieve Analysis of Breakthrough HIV-1 Sequences in the RV144 Vaccine Efficacy Trial
Source: PLoS Comput Biol. 2015 Feb 3;11(2):e1003973. doi: 10.1371/journal.pcbi.1003973 (PMC4315437; doi:10.1371/journal.pcbi.1003973)
Supplement: S1 Table — Numbers of HIV-1 protein sequences measured from the n = 109 HIV-1 CRF01_AE infected subjects in the RV144 trial: Vaccine immunogen proteins. (DOC) [file pcbi.1003973.s010.doc]

**Table S1. Numbers of HIV-1 protein sequences measured from the n = 109^a^ HIV-1 CRF01_AE infected subjects in the RV144 trial: Vaccine immunogen proteins.**

|  | **Env** | **Gag** | **Pro** |
| --- | --- | --- | --- |
|  | **All, Vac, Plac** | **All, Vac, Plac** | **All, Vac, Plac** |
| **Total number of sequences** | 922, 344, 578 | 917, 343, 574 | 913, 341, 572 |
| **Minimum seqs per subject** | 3, 4, 3 | 1, 1, 3 | 1, 1, 3 |
| **Median seqs per subject** | 10, 8, 10 | 10, 7, 10 | 10, 7, 10 |
| **Maximum seqs per subject** | 14, 14, 13 | 16, 16, 14 | 16, 16, 14 |

^a^ The HIV-1 CRF01_AE infected subject that was a secondary infection in a transmission pair is excluded.
