# Supplementary material for: Comprehensive Sieve Analysis of Breakthrough HIV-1 Sequences in the RV144 Vaccine Efficacy Trial
Source: PLoS Comput Biol. 2015 Feb 3;11(2):e1003973. doi: 10.1371/journal.pcbi.1003973 (PMC4315437; doi:10.1371/journal.pcbi.1003973)
Supplement: S2 Table — Numbers of HIV-1 protein sequences measured from the n = 109 HIV-1 CRF01_AE infected subjects in the RV144 trial: Non-vaccine immunogen proteins. (DOC) [file pcbi.1003973.s011.doc]

**Table S2. Numbers of HIV-1 protein sequences measured from the n = 109^a^ HIV-1 CRF01_AE infected subjects in the RV144 trial: Non-vaccine immunogen proteins.**

|  | **Nef** | **Rev** | **Tat** | **Vif** | **Vpr** | **Vpu** |
| --- | --- | --- | --- | --- | --- | --- |
|  | **All, Vac, Plac** | **All, Vac, Plac** | **All, Vac, Plac** | **All, Vac, Plac** | **All, Vac, Plac** | **All, Vac, Plac** |
| **Total no. seqs** | 925, 345, 580 | 925, 344, 581 | 920, 341, 579 | 1220, 439, 781 | 1220, 439, 781 | 927, 345, 582 |
| **Min seqs/subject** | 3, 4, 3 | 3, 4, 3 | 3, 4, 3 | 3, 5, 3 | 3, 5, 3 | 3, 4, 3 |
| **Median seqs/subj** | 10, 8, 10 | 10, 8, 10 | 10, 7, 10 | 10, 10, 11 | 10, 10, 11 | 10, 8, 10 |
| **Max seqs/subject** | 14, 14, 13 | 14, 14, 13 | 14, 14, 13 | 24, 24, 22 | 24, 24, 22 | 14, 14, 13 |

^a^ The HIV-1 CRF01_AE infected subject that was a secondary infection in a transmission pair is excluded.
