# Supplementary material for: Comprehensive Sieve Analysis of Breakthrough HIV-1 Sequences in the RV144 Vaccine Efficacy Trial
Source: PLoS Comput Biol. 2015 Feb 3;11(2):e1003973. doi: 10.1371/journal.pcbi.1003973 (PMC4315437; doi:10.1371/journal.pcbi.1003973)
Supplement: S3 Table — Biological annotation of the identified signature sites in vaccine proteins. (DOC) [file pcbi.1003973.s012.doc]

Table S3. Biological annotation of the identified signature sites in vaccine proteins.

| **Position^1^** | **vMismatch^2^** | | **Context^3^** | **Hotspot^4^** | **Contactsite^4^** | **nAb-site^4^** | **EPIMAP^4^** | **MHC-I epitope hotspot^5^** | **MHC-II epitope hotspot^5^** | **Physico chemical sieve^6^** |
| --- | --- | --- | --- | --- | --- | --- | --- | --- | --- | --- |
| Env 6 | T | |  | T | F | F | F | 92TH:B |  |  |
| Env 19 | F | | Signal peptide | T | F | F | F | 92TH:W |  |  |
| Env 169 | | F | V2 | T | T | T | T | MN:W | MN:B |  |
| Env 181 | | T | V2; α4β7 | T | F | T | T | 92TH:B; A244:B; MN:W | MN:W |  |
| Env 268 | | T |  | T | F | T | F | 92TH:W; A244:W; MN:W |  |  |
| Env 317 | | T | V3; conserved co- receptor binding site | T | T | T | T |  | MN:B |  |
| Env 343 | | F |  | F | F | T | F | 92TH:B; A244:B; MN:W | 92TH:B; MN:B |  |
| Env 353 | | F | Co-receptor binding site | F | T | F | F | 92TH:B; A244:B; MN:B | MN:S |  |
| Env 369 | | T^7^ | CD4 binding site; “P” in conserved GGDPE; Contacts: b12, b13, f105, 48d, x5 | F | T | F | F |  |  |  |
| Env 379 | | T |  | F | F | F | F | 92TH:W; A244:B |  |  |
| Env 413 | | T |  | T | F | T | F | 92TH:B | MN:S |  |
| Env 424 | | F | Contacts: f105 | F | T | F | F | 92TH:S; A244:S; MN:B | MN:B |  |
| Gag 11 | | F |  |  |  |  |  | LAI:B |  |  |
| Gag 30 | | F | Putatively always Arg or Lys in clade E; 9 of 43 in Vac are other (vs. 2 of 66 Plac) |  |  |  |  | LAI:B |  |  |
| Gag 482 | | T | Overlaps Pol 51 in reading frame 3 |  |  |  |  | LAI:B | LAI:B |  |
| Pol 12 | | F |  |  |  |  |  |  | LAI:B |  |
| Pol 44 | | F |  |  |  |  |  |  |  |  |
| Pol 51 | | T | Overlaps Gag 482 in reading frame 1 |  |  |  |  |  |  | Hydrophobic, Proline, Tiny, z2, z3, z4, z5 |
| Pol 66 | |  |  |  |  |  |  |  |  |  |

^1^HXB2 Numbering

^2^Indicates whether the putative sieve effect at the position was “vMismatch” (having greater distance to the vaccine amino acid in the placebo group than in the vaccine group, a result previously reported for site Env 181)

^3^Presents information regarding the location (“context”) of the site

^4^Indicates True or False (T/F) whether the site is in each of the indicated site sets (defined in Methods)

^5^Sites that are in significantly more predicted *MHC-I* or *MHC-II* T cell epitopes in the vaccine immunogen sequence(s) than would be expected by uniformly distributed epitope locations, with S, W, and B defined in Methods (sub-section “HLA-associated sites”)

^6^Indication of which of the ten (Taylor) physic-chemical properties or “z-scales” (if any) were found to be significantly associated with treatment group at the site

^7^The signature site at Env 367 shows evidence of a “vMatch” effect versus the clade AE sequence amino acid (L) and evidence of a “vMismatch” sieve effect versus the clade B vaccine sequence amino acid (P)
