# Supplementary material for: Comprehensive Sieve Analysis of Breakthrough HIV-1 Sequences in the RV144 Vaccine Efficacy Trial
Source: PLoS Comput Biol. 2015 Feb 3;11(2):e1003973. doi: 10.1371/journal.pcbi.1003973 (PMC4315437; doi:10.1371/journal.pcbi.1003973)
Supplement: S4 Table — Biological annotation of the identified signature sites in non-vaccine proteins. (DOC) [file pcbi.1003973.s013.doc]

Table S4. Biological annotation of the identified signature sites in non-vaccine proteins.

| **Position^1^** | **vMismatch^2^** | **Context^3^** | **Hotspot^4^** | **Contactsite^4^** | **nAb-site^4^** | **EPIMAP^4^** | **MHC-I epitope hotspot^5^** | **MHC-II epitope hotspot^5^** | **Physico chemical sieve^6^** |
| --- | --- | --- | --- | --- | --- | --- | --- | --- | --- |
| Env 640 | T |  | F | T | F |  |  |  |  |
| Env 732 | F | Overlaps Rev 39 in reading frame 2 | F | F | F |  |  |  | Hydrophobic, z1 |
| Env 777 | T |  | F | F | F |  |  |  | Small, z2 |
| Env 829 | F |  | F | F | F |  |  |  |  |
| Nef 19 | F |  |  |  |  |  |  |  |  |
| Nef 27 | T |  |  |  |  |  |  |  |  |
| Nef 56 | T |  |  |  |  |  | S |  |  |
| Nef 63 | F |  |  |  |  |  |  |  |  |
| Nef 120 | T |  |  |  |  |  | W |  |  |
| Nef 125 | F |  |  |  |  |  |  |  | Charged, z3 |
| Nef 156 | T |  |  |  |  |  |  |  |  |
| Pol 297 | F |  |  |  |  |  |  |  |  |
| Pol 328 | F |  |  |  |  |  |  |  |  |
| Pol 497 | T |  |  |  |  |  |  |  | Polar, z1, z2, z3, z4, z5 |
| Pol 590 | F |  |  |  |  |  |  |  |  |
| Pol 816 | F |  |  |  |  |  |  |  |  |
| Rev 7 | F | Overlaps Tat 53 in reading frame 2 |  |  |  |  |  |  |  |
| Rev 39 | F | Overlaps Env 732 in reading frame 3 |  |  |  |  | B |  |  |
| Rev 55 | F | Overlaps Tat 101 in reading frame 1 |  |  |  |  | W |  |  |
| Rev 92 | T |  |  |  |  |  |  |  | Hydrophobic |
| Tat 3 | T |  |  |  |  |  | S |  |  |
| Tat 17 | F |  |  |  |  |  |  |  |  |
| Tat 36 | T |  |  |  |  |  | W |  |  |
| Tat 53 | F | Overlaps Rev 7 in reading frame 3 |  |  |  |  |  |  |  |
| Tat 64 | F |  |  |  |  |  |  |  |  |
| Tat 81 | F |  |  |  |  |  |  |  |  |
| Tat 101 | F | Overlaps Rev 55 in reading frame 2 |  |  |  |  |  |  |  |
| Vif 22 | F |  |  |  |  |  |  |  |  |
| Vif 31 | T |  |  |  |  |  | B |  | Small |
| Vif 93 | F |  |  |  |  |  |  |  |  |
| Vif 130 | F |  |  |  |  |  |  |  |  |
| Vif 134 | F |  |  |  |  |  |  |  |  |
| Vif 190 | F |  |  |  |  |  | S |  |  |
| Vpr 8 | F |  |  |  |  |  |  |  |  |
| Vpu 27 | T |  |  |  |  |  | B |  |  |
| Vpu 30 | T |  |  |  |  |  | B |  | Hydrophobic, z2, z5 |
| Vpu 46 | T |  |  |  |  |  |  |  | Hydrophobic |

^1^HXB2 Numbering

^2^Indicates whether the putative sieve effect at the position was “vMismatch” (having greater distance to the vaccine amino acid in the placebo group than in the vaccine group, a result previously reported for site Env 181)

^3^Presents information regarding the location (“context”) of the site

^4^Indicates True or False (T/F) whether the site is in each of the indicated site sets (defined in Methods)

^5^Sites that are in significantly more predicted *MHC-I* or *MHC-II* T cell epitopes in the vaccine immunogen sequence(s) than would be expected by uniformly distributed epitope locations, with S, W, and B defined in Methods (sub-section “HLA-associated sites”)

^6^Indication of which of the ten (Taylor) physico-chemical properties or “z-scales” (if any) were found to be significantly associated with treatment group at the site
