# Supplementary material for: Comprehensive Sieve Analysis of Breakthrough HIV-1 Sequences in the RV144 Vaccine Efficacy Trial
Source: PLoS Comput Biol. 2015 Feb 3;11(2):e1003973. doi: 10.1371/journal.pcbi.1003973 (PMC4315437; doi:10.1371/journal.pcbi.1003973)
Supplement: S5 Table — Significant 9-mer sieve effects in vaccine proteins (by KmerScan). (DOC) [file pcbi.1003973.s014.doc]

**Table S5. Significant 9-mer sieve effects in vaccine proteins (by KmerScan)**.

| **Position^1^** | **Ref** | **p-value** | **q-value** | **vMismatch** |
| --- | --- | --- | --- | --- |
| Env 132 | MN | 0.022 | 0.957 | T |
| Env 172 | BothAE | 0.031 | 0.957 | T |
| Env 173 | BothAE | 0.003 | 0.957 | T |
| Env 174 | MN | 0.022 | 0.957 | T |
| Env 198 | BothAE | 0.031 | 0.957 | T |
| Env 199 | BothAE | 0.022 | 0.957 | T |
| Env 292 | MN | 0.023 | 0.957 | T |
| Env 293 | MN | 0.047 | 0.957 | T |
| Env 350 | MN | 0.036 | 0.957 | F |
| Env 358 | MN | 0.042 | 0.957 | T |
| Env 359 | MN | 0.03 | 0.957 | T |
| Env 360 | MN | 0.021 | 0.957 | T |
| Env 361 | MN | 0.002 | 0.655 | T |
| Env 362 | MN | 0.012 | 0.957 | T |
| Env 364 | BothAE | 0.017 | 0.957 | F |
| Env 367 | BothAE | 0.039 | 0.957 | F |
| Env 368 | BothAE | 0.033 | 0.957 | F |
| Env 369 | MN | 0.04 | 0.957 | T |
| Env 381 | BothAE | 0.047 | 0.957 | F |
| Env 422 | MN | 0.011 | 0.957 | F |
| Env 423 | MN | 0.013 | 0.957 | F |
| Env 509 | 92TH | 0.031 | 0.957 | F |
| Gag 52 | LAI | 0.045 | 1 | F |
| Gag 61 | LAI | 0.031 | 1 | T |
| Gag 62 | LAI | 0.029 | 1 | T |
| Gag 63 | LAI | 0.034 | 1 | T |
| Gag 64 | LAI | 0.034 | 1 | T |
| Gag 65 | LAI | 0.033 | 1 | T |
| Gag 384 | LAI | 0.033 | 1 | F |
| Gag 489 | LAI | 0.007 | 1 | F |
| Gag 490 | LAI | 0.025 | 1 | F |
| Gag 491 | LAI | 0.039 | 1 | F |
| Gag 492 | LAI | 0.02 | 1 | F |
| Pol 111 | LAI | 0.043 | 0.994 | T |
| Pol 112 | LAI | 0.049 | 0.994 | T |
| Pol 115 | LAI | 0.036 | 0.994 | T |
| Pol 116 | LAI | 0.031 | 0.994 | T |
| Pol 117 | LAI | 0.034 | 0.994 | T |

^1^HXB2 numbering indicating 9-mer start site.
