# Supplementary material for: Comprehensive Sieve Analysis of Breakthrough HIV-1 Sequences in the RV144 Vaccine Efficacy Trial
Source: PLoS Comput Biol. 2015 Feb 3;11(2):e1003973. doi: 10.1371/journal.pcbi.1003973 (PMC4315437; doi:10.1371/journal.pcbi.1003973)
Supplement: S6 Table — Significant 9-mer sieve effects in non-vaccine proteins (by KmerScan). (DOC) [file pcbi.1003973.s015.doc]

**Table S6. Significant 9-mer sieve effects in non-vaccine proteins (by KmerScan)**.

| **Position^1^** | **Ref** | **p-value** | **q-value** | **vMismatch** |
| --- | --- | --- | --- | --- |
| Nef 13 | ConAE | 0.012 | 0.292 | F |
| Nef 14 | ConAE | 0.027 | 0.372 | F |
| Nef 15 | ConAE | 0.017 | 0.303 | F |
| Nef 16 | ConAE | 0.019 | 0.304 | F |
| Nef 37 | ConAE | 0.035 | 0.372 | T |
| Nef 121 | ConAE | 0.009 | 0.26 | F |
| Nef 122 | ConAE | 0.006 | 0.216 | F |
| Nef 123 | ConAE | 0.007 | 0.216 | F |
| Nef 124 | ConAE | 0.007 | 0.216 | F |
| Nef 125 | ConAE | 0.004 | 0.216 | F |
| Nef 140 | ConAE | 0.043 | 0.413 | T |
| Nef 141 | ConAE | 0.005 | 0.216 | T |
| Nef 148 | ConAE | 0.032 | 0.372 | T |
| Nef 151 | ConAE | 0.033 | 0.372 | T |
| Nef 152 | ConAE | 0.007 | 0.216 | T |
| Nef 153 | ConAE | 0.016 | 0.302 | T |
| Nef 154 | ConAE | 0.014 | 0.302 | T |
| Nef 155 | ConAE | 0.03 | 0.372 | T |
| Nef 156 | ConAE | 0.043 | 0.413 | T |
| Nef 158 | ConAE | 0.03 | 0.372 | T |
| Rev 25 | ConAE | 0.018 | 0.349 | F |
| Rev 34 | ConAE | 0.043 | 0.432 | F |
| Rev 35 | ConAE | 0.021 | 0.349 | F |
| Rev 36 | ConAE | 0.018 | 0.349 | F |
| Rev 37 | ConAE | 0.019 | 0.349 | F |
| Rev 38 | ConAE | 0.014 | 0.349 | F |
| Rev 39 | ConAE | 0.021 | 0.349 | F |
| Rev 81 | ConAE | 0.036 | 0.432 | T |
| Rev 82 | ConAE | 0.028 | 0.405 | T |
| Rev 84 | ConAE | 0.009 | 0.349 | T |
| Rev 85 | ConAE | 0.043 | 0.432 | T |
| **Tat 1** | **ConAE** | **0.008** | **0.085** | **T** |
| **Tat 2** | **ConAE** | **0.008** | **0.085** | **T** |
| **Tat 3** | **ConAE** | **0.013** | **0.085** | **T** |
| **Tat 9** | **ConAE** | **0.039** | **0.135** | **F** |
| **Tat 10** | **ConAE** | **0.022** | **0.12** | **F** |
| **Tat 11** | **ConAE** | **0.047** | **0.146** | **F** |
| **Tat 23** | **ConAE** | **0.035** | **0.135** | **T** |
| **Tat 24** | **ConAE** | **0.033** | **0.135** | **T** |
| **Tat 28** | **ConAE** | **0.005** | **0.085** | **T** |
| **Tat 29** | **ConAE** | **0.005** | **0.085** | **T** |
| **Tat 30** | **ConAE** | **0.002** | **0.085** | **T** |
| **Tat 31** | **ConAE** | **0.003** | **0.085** | **T** |
| **Tat 32** | **ConAE** | **0.033** | **0.135** | **T** |
| **Tat 33** | **ConAE** | **0.039** | **0.135** | **T** |
| **Tat 34** | **ConAE** | **0.046** | **0.146** | **T** |
| **Tat 35** | **ConAE** | **0.046** | **0.146** | **T** |
| **Tat 39** | **ConAE** | **0.013** | **0.085** | **F** |
| **Tat 40** | **ConAE** | **0.008** | **0.085** | **F** |
| **Tat 41** | **ConAE** | **0.031** | **0.135** | **F** |
| **Tat 42** | **ConAE** | **0.014** | **0.085** | **F** |
| **Tat 43** | **ConAE** | **0.014** | **0.085** | **F** |
| **Tat 44** | **ConAE** | **0.013** | **0.085** | **F** |
| **Tat 45** | **ConAE** | **0.002** | **0.085** | **F** |
| **Tat 46** | **ConAE** | **0.011** | **0.085** | **F** |
| **Tat 47** | **ConAE** | **0.011** | **0.085** | **F** |
| **Tat 48** | **ConAE** | **0.025** | **0.121** | **F** |
| **Tat 49** | **ConAE** | **0.025** | **0.121** | **F** |
| **Tat 62** | **ConAE** | **0.039** | **0.135** | **F** |
| **Tat 63** | **ConAE** | **0.015** | **0.085** | **F** |
| **Tat 64** | **ConAE** | **0.034** | **0.135** | **F** |
| Vif 1 | ConAE | 0.035 | 0.809 | T |
| Vif 23 | ConAE | 0.009 | 0.771 | T |
| Vif 24 | ConAE | 0.009 | 0.771 | T |
| Vif 25 | ConAE | 0.049 | 0.809 | T |
| Vif 26 | ConAE | 0.043 | 0.809 | T |
| Vif 27 | ConAE | 0.016 | 0.771 | T |
| Vif 28 | ConAE | 0.017 | 0.771 | T |
| Vif 29 | ConAE | 0.034 | 0.809 | T |
| Vif 132 | ConAE | 0.038 | 0.809 | F |
| Vif 169 | ConAE | 0.044 | 0.809 | F |
| Vif 170 | ConAE | 0.034 | 0.809 | F |
| Vpr 32 | ConAE | 0.024 | 0.792 | F |
| Vpu 22 | ConAE | 0.05 | 0.456 | T |
| Vpu 23 | ConAE | 0.037 | 0.456 | T |
| Vpu 25 | ConAE | 0.046 | 0.456 | T |
| Vpu 26 | ConAE | 0.04 | 0.456 | T |
| Vpu 27 | ConAE | 0.026 | 0.456 | T |
| Vpu 28 | ConAE | 0.045 | 0.456 | T |
| Vpu 29 | ConAE | 0.023 | 0.456 | T |
| **Vpu 30** | **ConAE** | **0.001** | **0.045** | **T** |

^1^HXB2 numbering indicating 9-mer start site.
