# Supplementary material for: Comprehensive Sieve Analysis of Breakthrough HIV-1 Sequences in the RV144 Vaccine Efficacy Trial
Source: PLoS Comput Biol. 2015 Feb 3;11(2):e1003973. doi: 10.1371/journal.pcbi.1003973 (PMC4315437; doi:10.1371/journal.pcbi.1003973)
Supplement: S7 Table — Physico-chemical Properties (PCP) site-scanning results in vaccine proteins. (DOC) [file pcbi.1003973.s016.doc]

**Table S7. Physico-chemical Properties (PCP) site-scanning results in vaccine proteins**.

| **Position^1^** | **Grp^2^|property^3^:p-value (q-value)** | | | | | | |
| --- | --- | --- | --- | --- | --- | --- | --- |
| Env 63 | V|z1:0.029 (1.000) |  |  |  |  |  |  |
| Env 351 | V|z1:0.030 (1.000) | P|hydrophobic:0.040 (1.000) |  |  |  |  |  |
| Gag 465 | P|z2:0.014 (1.000) |  |  |  |  |  |  |
| Pol 30 | P|hydrophobic:0.042 (1.000) |  |  |  |  |  |  |
| Pol 51 | P|z2:0.016 (1.000) | P|z3:0.001 (0.189) | P|z4:0.006 (0.865) | P|z5:0.004 (0.612) | V|hydrophobic:0.039 (1.000) | P|proline:0.039 (1.000) | P|tiny:0.011 (1.000) |

^1^HXB2 Numbering

^2^Direction of effect: the physicochemical property is enriched in the Placebo (Grp = P) or the Vaccine (Grp = V) group

^3^One of the ten (Taylor ) physicochemical properties or five “z-scales” that was found to be significantly associated with treatment group at the 9-mer beginning at the site
