# Supplementary material for: Comprehensive Sieve Analysis of Breakthrough HIV-1 Sequences in the RV144 Vaccine Efficacy Trial
Source: PLoS Comput Biol. 2015 Feb 3;11(2):e1003973. doi: 10.1371/journal.pcbi.1003973 (PMC4315437; doi:10.1371/journal.pcbi.1003973)
Supplement: S8 Table — Physico-chemical Properties (PCP) site-scanning results in non-vaccine proteins. (DOC) [file pcbi.1003973.s017.doc]

**Table S8. Physico-chemical Properties (PCP) site-scanning results in non-vaccine proteins**.

| **Position^1^** | **Grp^2^|property^3^:p-value (q-value)** | | | | | |
| --- | --- | --- | --- | --- | --- | --- |
| Env 732 | P|z1:0.028 (1.000) | V|hydrophobic:0.033 (1.000) |  |  |  |  |
| Env 777 | V|z2:0.045 (1.000) | P|small:0.039 (1.000) |  |  |  |  |
| Env 787 | V|z3:0.043 (1.000) |  |  |  |  |  |
| Pol 497 | V|z1:0.039 (1.000) | V|z2:0.039 (1.000) | P|z3:0.039 (1.000) | P|z4:0.039 (1.000) | P|z5:0.039 (1.000) | V|polar:0.039 (1.000) |
| Pol 638 | P|z2:0.038 (1.000) | V|z5:0.017 (1.000) |  |  |  |  |
| Nef 125 | V|z3:0.033 (1.000) | V|charged:0.016 (1.000) |  |  |  |  |
| Vpu 30 | P|z2:0.032 (1.000) | V|z5:0.028 (1.000) | V|hydrophobic:0.001 (0.103) |  |  |  |
| Vpu 46 | P|hydrophobic:0.037 (1.000) |  |  |  |  |  |
| Rev 84 | P|hydrophobic:0.039 (1.000) |  |  |  |  |  |
| Rev 92 | P|hydrophobic:0.039 (1.000) |  |  |  |  |  |
| Vif 31 | P|small:0.020 (1.000) |  |  |  |  |  |

^1^HXB2 Numbering

^2^Direction of effect: the physicochemical property is enriched in the Placebo (Grp = P) or the Vaccine (Grp = V) group

^3^One of the ten (Taylor ) physicochemical properties or five “z-scales” that was found to be significantly associated with treatment group at the 9-mer beginning at the site
