# Supplementary material for: Comprehensive Sieve Analysis of Breakthrough HIV-1 Sequences in the RV144 Vaccine Efficacy Trial
Source: PLoS Comput Biol. 2015 Feb 3;11(2):e1003973. doi: 10.1371/journal.pcbi.1003973 (PMC4315437; doi:10.1371/journal.pcbi.1003973)
Supplement: S9 Table — Physico-chemical Properties (PCP) 3-mer results in vaccine proteins. (DOC) [file pcbi.1003973.s018.doc]

**Table S9. Physico-chemical Properties (PCP) 3-mer results in vaccine proteins**.

| **Position^1^** | **Grp^2^|property^3^:p-value (q-value)** | | |
| --- | --- | --- | --- |
| Env 23 | P|z1:0.046 (1.000) | P|z3:0.036 (1.000) |  |
| Env 61 | P|z5:0.049 (1.000) | P|proline:0.039 (1.000) |  |
| Env 62 | P|z5:0.029 (1.000) |  |  |
| Env 90 | V|z1:0.039 (1.000) | P|z4:0.035 (1.000) |  |
| Env 169 | V|z3:0.027 (1.000) |  |  |
| Env 173 | P|z2:0.038 (1.000) |  |  |
| Env 199 | P|z1:0.034 (1.000) |  |  |
| Env 200 | P|z1:0.013 (1.000) |  |  |
| Env 315 | V|z3:0.026 (1.000) |  |  |
| Env 378 | V|z1:0.037 (1.000) |  |  |
| Env 379 | V|z1:0.037 (1.000) |  |  |
| Env 390 | P|z4:0.021 (1.000) |  |  |
| Env 422 | V|z1:0.035 (1.000) |  |  |
| Env 696 | V|z5:0.049 (1.000) |  |  |
| Env 698 | V|z5:0.036 (1.000) |  |  |
| Gag 60 | P|z1:0.016 (1.000) |  |  |
| Gag 102 | P|z4:0.035 (1.000) |  |  |
| Gag 103 | V|z5:0.031 (1.000) |  |  |
| Gag 104 | V|z5:0.031 (1.000) |  |  |
| Gag 367 | P|z2:0.040 (1.000) |  |  |
| Gag 477 | V|z2:0.029 (1.000) |  |  |
| Pol 49 | P|z3:0.010 (1.000) | P|z5:<0.001 (0.030) | V|hydrophobic:0.024 (1.000) |
| Pol 50 | P|z2:0.010 (1.000) |  |  |

^1^HXB2 Numbering

^2^Direction of effect: the physicochemical property is enriched in the Placebo (Grp = P) or the Vaccine (Grp = V) group

^3^One of the ten (Taylor ) physicochemical properties or five “z-scales” that was found to be significantly associated with treatment group at the 9-mer beginning at the site
