# Supplementary material for: Comprehensive Sieve Analysis of Breakthrough HIV-1 Sequences in the RV144 Vaccine Efficacy Trial
Source: PLoS Comput Biol. 2015 Feb 3;11(2):e1003973. doi: 10.1371/journal.pcbi.1003973 (PMC4315437; doi:10.1371/journal.pcbi.1003973)
Supplement: S10 Table — Physico-chemical Properties (PCP) 3-mer results in non-vaccine proteins. (DOC) [file pcbi.1003973.s019.doc]

**Table S10. Physico-chemical Properties (PCP) 3-mer results in non-vaccine proteins**.

| **Position^1^** | **Grp^2^|property^3^:p-value (q-value)** | | | | |  |
| --- | --- | --- | --- | --- | --- | --- |
| Env 618 | V|z2:0.048 (1.000) |  |  |  |  |  |
| Env 636 | V|z5:0.037 (1.000) |  |  |  |  |  |
| Env 638 | V|z5:0.016 (1.000) |  |  |  |  |  |
| Env 730 | P|z2:0.032 (1.000) | V|z3:0.029 (1.000) |  |  |  |  |
| Env 731 | P|z2:0.026 (1.000) | V|z3:0.019 (1.000) | V|hydrophobic:0.044 (1.000) | V|small:0.028 (1.000) |  |  |
| Env 732 | P|z2:0.013 (1.000) | V|z3:0.018 (1.000) | P|charged:0.043 (1.000) | V|hydrophobic:0.013 (1.000) | V|small:0.008 (1.000) | V|tiny:0.021 (1.000) |
| Env 785 | V|aromatic:0.037 (1.000) |  |  |  |  |  |
| Env 786 | V|aromatic:0.037 (1.000) |  |  |  |  |  |
| Env 787 | V|aromatic:0.037 (1.000) |  |  |  |  |  |
| Env 816 | V|z2:0.036 (1.000) |  |  |  |  |  |
| Pol 495 | V|z1:0.019 (1.000) | V|z2:0.019 (1.000) | P|z3:0.019 (1.000) | P|z4:0.019 (1.000) | P|z5:0.019 (1.000) | V|polar:0.039 (1.000) |
| Pol 496 | V|z1:0.019 (1.000) | V|z2:0.019 (1.000) | P|z3:0.019 (1.000) | P|z4:0.019 (1.000) | P|z5:0.019 (1.000) | V|polar:0.039 (1.000) |
| Pol 497 | P|z3:0.031 (1.000) | P|z4:0.006 (1.000) | P|z5:0.006 (1.000) | V|polar:0.039 (1.000) |  |  |
| Pol 636 | P|z2:0.019 (1.000) | P|z3:0.033 (1.000) | V|z5:0.007 (1.000) |  |  |  |
| Pol 637 | P|z2:0.019 (1.000) | P|z3:0.033 (1.000) | V|z5:0.007 (1.000) |  |  |  |
| Pol 638 | P|z2:0.019 (1.000) | P|z3:0.049 (1.000) | V|z5:0.009 (1.000) |  |  |  |
| Nef 26 | P|z3:0.042 (1.000) |  |  |  |  |  |
| Nef 27 | P|z3:0.042 (1.000) |  |  |  |  |  |
| Nef 123 | V|z2:0.044 (1.000) | V|z3:0.016 (1.000) | V|charged:0.016 (1.000) |  |  |  |
| Nef 124 | V|z2:0.044 (1.000) | V|z3:0.016 (1.000) | V|charged:0.016 (1.000) |  |  |  |
| Nef 125 | V|z2:0.044 (1.000) | V|z3:0.016 (1.000) | V|charged:0.016 (1.000) |  |  |  |
| Nef 154 | V|charged:0.036 (1.000) |  |  |  |  |  |
| Nef 156 | V|charged:0.047 (1.000) |  |  |  |  |  |
| Rev 38 | P|z5:0.042 (1.000) |  |  |  |  |  |
| Rev 39 | P|z5:0.042 (1.000) |  |  |  |  |  |
| Rev 82 | V|z1:0.033 (1.000) |  |  |  |  |  |
| Rev 83 | V|z3:0.037 (1.000) |  |  |  |  |  |
| Rev 84 | V|z3:0.025 (1.000) |  |  |  |  |  |
| Tat 34 | P|z1:0.027 (1.000) |  |  |  |  |  |
| Tat 35 | P|z1:0.027 (1.000) |  |  |  |  |  |
| Tat 36 | V|z5:0.041 (1.000) |  |  |  |  |  |
| Tat 79 | P|z4:0.019 (1.000) |  |  |  |  |  |
| Tat 80 | P|z4:0.019 (1.000) |  |  |  |  |  |
| Vpu 28 | V|hydrophobic:0.001 (0.050) |  |  |  |  |  |
| Vpu 29 | V|hydrophobic:0.002 (0.050) |  |  |  |  |  |
| Vpu 30 | P|z2:0.016 (1.000) |  |  |  |  |  |
| Vpu 37 | P|z1:0.006 (0.526) |  |  |  |  |  |
| Vpu 45 | V|z1:0.043 (1.000) |  |  |  |  |  |
| Vpu 46 | V|z1:0.043 (1.000) |  |  |  |  |  |
| Vif 29 | P|small:0.044 (1.000) |  |  |  |  |  |
| Vif 30 | P|small:0.044 (1.000) |  |  |  |  |  |
| Vif 31 | P|small:0.020 (1.000) |  |  |  |  |  |

^1^HXB2 Numbering

^2^Direction of effect: the physicochemical property is enriched in the Placebo (Grp = P) or the Vaccine (Grp = V) group

^3^One of the ten (Taylor ) physicochemical properties or five “z-scales” that was found to be significantly associated with treatment group at the 9-mer beginning at the site
