# Supplementary material for: Comprehensive Sieve Analysis of Breakthrough HIV-1 Sequences in the RV144 Vaccine Efficacy Trial
Source: PLoS Comput Biol. 2015 Feb 3;11(2):e1003973. doi: 10.1371/journal.pcbi.1003973 (PMC4315437; doi:10.1371/journal.pcbi.1003973)
Supplement: S11 Table — Physico-chemical Properties (PCP) 9-mer results in vaccine proteins. (DOC) [file pcbi.1003973.s020.doc]

**Table S11. Physico-chemical Properties (PCP) 9-mer results in vaccine proteins**.

| **Position^1^** | **Grp^2^|property^3^:p-value (q-value)** | |
| --- | --- | --- |
| Env 55 | P|proline:0.039 (1.000) |  |
| Env 56 | P|proline:0.039 (1.000) |  |
| Env 57 | P|proline:0.039 (1.000) |  |
| Env 58 | P|proline:0.039 (1.000) |  |
| Env 59 | P|proline:0.039 (1.000) |  |
| Env 60 | P|proline:0.039 (1.000) |  |
| Env 61 | P|proline:0.039 (1.000) |  |
| Env 195 | P|z1:0.012 (1.000) |  |
| Env 196 | P|z1:0.016 (1.000) |  |
| Env 197 | P|z1:0.016 (1.000) |  |
| Env 198 | P|z1:0.010 (1.000) |  |
| Env 199 | P|z1:0.010 (1.000) |  |
| Env 327 | P|z4:0.046 (1.000) |  |
| Env 353 | V|proline:0.044 (1.000) |  |
| Env 378 | V|z1:0.033 (1.000) |  |
| Env 379 | V|z1:0.033 (1.000) |  |
| Env 385 | P|z5:0.039 (1.000) |  |
| Env 688 | P|z3:0.038 (1.000) |  |
| Env 693 | V|z5:0.042 (1.000) |  |
| Env 694 | V|z5:0.035 (1.000) |  |
| Env 695 | V|z5:0.011 (1.000) |  |
| Env 696 | V|z5:0.021 (1.000) |  |
| Env 697 | V|z5:0.021 (1.000) |  |
| Gag 116 | V|z1:0.039 (1.000) |  |
| Gag 361 | P|z2:0.028 (1.000) |  |
| Pol 52 | V|z3:0.031 (1.000) |  |

^1^HXB2 Numbering

^2^Direction of effect: the physicochemical property is enriched in the Placebo (Grp = P) or the Vaccine (Grp = V) group

^3^One of the ten (Taylor ) physicochemical properties or five “z-scales” that was found to be significantly associated with treatment group at the 9-mer beginning at the site
