# Supplementary material for: Comprehensive Sieve Analysis of Breakthrough HIV-1 Sequences in the RV144 Vaccine Efficacy Trial
Source: PLoS Comput Biol. 2015 Feb 3;11(2):e1003973. doi: 10.1371/journal.pcbi.1003973 (PMC4315437; doi:10.1371/journal.pcbi.1003973)
Supplement: S12 Table — Physico-chemical Properties (PCP) 9-mer results in non-vaccine proteins. (DOC) [file pcbi.1003973.s021.doc]

**Table S12. Physico-chemical Properties (PCP) 9-mer results in non-vaccine proteins**.

|  |  | |  | |  | |  | |
| --- | --- | --- | --- | --- | --- | --- | --- | --- |
| **Position^1^** | | **Grp^2^|property^3^:p-value (q-value)** | | | | | | |
| Env 601 | | P|z5:0.037 (1.000) | | P|proline:0.042 (1.000) | |  | |  |
| Env 602 | | P|z5:0.037 (1.000) | | P|proline:0.042 (1.000) | |  | |  |
| Env 603 | | P|proline:0.023 (1.000) | |  | |  | |  |
| Env 604 | | P|proline:0.023 (1.000) | |  | |  | |  |
| Env 605 | | P|proline:0.023 (1.000) | | V|small:0.028 (1.000) | |  | |  |
| Env 632 | | V|z5:0.001 (1.000) | | V|hydrophobic:0.013 (1.000) | |  | |  |
| Env 633 | | V|z5:0.016 (1.000) | |  | |  | |  |
| Env 634 | | V|z5:0.016 (1.000) | |  | |  | |  |
| Env 635 | | V|z5:0.018 (1.000) | |  | |  | |  |
| Env 730 | | V|z3:0.011 (1.000) | |  | |  | |  |
| Env 731 | | V|z3:0.011 (1.000) | |  | |  | |  |
| Env 732 | | V|z3:0.029 (1.000) | | P|z4:0.022 (1.000) | |  | |  |
| Pol 458 | | P|z1:0.037 (1.000) | |  | |  | |  |
| Pol 491 | | P|z3:0.033 (1.000) | | P|z4:0.024 (1.000) | | P|z5:0.006 (1.000) | |  |
| Pol 604 | | V|z5:0.042 (1.000) | |  | |  | |  |
| Pol 608 | | P|z2:0.038 (1.000) | |  | |  | |  |
| Pol 609 | | P|z2:0.038 (1.000) | |  | |  | |  |
| Pol 611 | | V|z3:0.038 (1.000) | |  | |  | |  |
| Pol 612 | | P|z2:0.028 (1.000) | | V|z3:0.026 (1.000) | |  | |  |
| Pol 613 | | V|z3:0.003 (1.000) | |  | |  | |  |
| Pol 614 | | V|z3:0.003 (1.000) | |  | |  | |  |
| Pol 630 | | P|z2:0.010 (1.000) | | V|z5:0.007 (1.000) | |  | |  |
| Pol 631 | | P|z2:0.010 (1.000) | | V|z5:0.007 (1.000) | |  | |  |
| Pol 632 | | P|z2:0.024 (1.000) | | V|z5:0.011 (1.000) | |  | |  |
| Pol 633 | | P|z2:0.025 (1.000) | | P|z3:0.046 (1.000) | | V|z5:0.014 (1.000) | | V|polar:0.021 (1.000) |
| Pol 634 | | P|z2:0.018 (1.000) | | V|z5:0.025 (1.000) | |  | |  |
| Pol 635 | | P|z2:0.018 (1.000) | | V|z5:0.025 (1.000) | |  | |  |
| Pol 636 | | P|z2:0.014 (1.000) | | V|z5:0.014 (1.000) | |  | |  |
| Pol 637 | | V|z5:0.014 (1.000) | |  | |  | |  |
| Pol 638 | | V|z5:0.005 (1.000) | |  | |  | |  |
| Pol 706 | | P|z4:0.039 (1.000) | |  | |  | |  |
| Pol 707 | | P|z4:0.039 (1.000) | |  | |  | |  |
| Pol 708 | | P|z4:0.039 (1.000) | |  | |  | |  |
| Pol 709 | | P|z4:0.039 (1.000) | |  | |  | |  |
| Nef 58 | | V|z4:0.012 (1.000) | |  | |  | |  |
| Nef 59 | | V|z4:0.014 (1.000) | |  | |  | |  |
| Nef 117 | | V|z3:0.016 (0.718) | | V|charged:0.016 (0.291) | |  | |  |
| Nef 118 | | V|z3:0.016 (0.718) | | V|charged:0.016 (0.291) | |  | |  |
| Nef 119 | | V|z3:0.016 (0.718) | | V|charged:0.016 (0.291) | |  | |  |
| Nef 120 | | V|z3:0.016 (0.718) | | V|charged:0.016 (0.291) | |  | |  |
| Nef 121 | | V|charged:0.016 (0.291) | |  | |  | |  |
| Nef 122 | | V|z2:0.013 (0.930) | | V|z3:0.026 (0.718) | | V|charged:0.016 (0.291) | |  |
| Nef 123 | | V|z2:0.013 (0.930) | | V|z3:0.026 (0.718) | | V|charged:0.016 (0.291) | |  |
| Nef 124 | | V|z2:0.013 (0.930) | | V|z3:0.026 (0.718) | | V|charged:0.016 (0.291) | |  |
| Nef 125 | | V|z3:0.014 (0.718) | | V|charged:0.016 (0.291) | |  | |  |
| Nef 147 | | V|negative:0.032 (1.000) | |  | |  | |  |
| Nef 148 | | V|charged:0.010 (0.291) | | V|negative:0.001 (0.063) | |  | |  |
| Nef 149 | | V|charged:0.011 (0.291) | | V|negative:0.001 (0.063) | |  | |  |
| Nef 150 | | V|charged:0.009 (0.291) | | V|negative:<0.001 (0.059) | |  | |  |
| Nef 201 | | P|proline:0.028 (1.000) | |  | |  | |  |
| Rev 9 | | P|hydrophobic:0.036 (1.000) | |  | |  | |  |
| Rev 10 | | P|hydrophobic:0.022 (1.000) | |  | |  | |  |
| Rev 11 | | P|hydrophobic:0.040 (1.000) | |  | |  | |  |
| Rev 38 | | V|z2:0.047 (0.940) | | P|z5:0.032 (1.000) | |  | |  |
| Rev 76 | | V|z1:0.018 (1.000) | |  | |  | |  |
| Rev 77 | | V|z3:0.019 (1.000) | |  | |  | |  |
| Rev 78 | | V|z3:0.015 (1.000) | |  | |  | |  |
| Rev 92 | | V|z2:0.031 (0.940) | |  | |  | |  |
| Rev 93 | | V|z2:0.010 (0.940) | | P|small:0.027 (1.000) | |  | |  |
| Rev 94 | | V|z2:0.024 (0.940) | | P|small:0.024 (1.000) | | P|tiny:0.026 (1.000) | |  |
| Rev 95 | | P|small:0.024 (1.000) | |  | |  | |  |
| Tat 1 | | P|z1:0.048 (1.000) | |  | |  | |  |
| Tat 2 | | P|z1:0.048 (1.000) | |  | |  | |  |
| Tat 32 | | V|z5:0.032 (1.000) | |  | |  | |  |
| Tat 33 | | V|z5:0.019 (1.000) | |  | |  | |  |
| Tat 34 | | V|z5:0.031 (1.000) | |  | |  | |  |
| Tat 35 | | V|z5:0.019 (1.000) | |  | |  | |  |
| Tat 36 | | V|z5:0.036 (1.000) | |  | |  | |  |
| Tat 62 | | P|z1:0.047 (1.000) | |  | |  | |  |
| Tat 63 | | P|z1:0.020 (1.000) | |  | |  | |  |
| Tat 64 | | P|z1:0.011 (1.000) | |  | |  | |  |
| Tat 73 | | P|z4:0.028 (0.194) | |  | |  | |  |
| Tat 73a | | P|z4:0.021 (0.194) | |  | |  | |  |
| Tat 73b | | P|z4:0.029 (0.194) | |  | |  | |  |
| Vif 23 | | P|small:0.035 (1.000) | |  | |  | |  |
| Vif 24 | | P|small:0.035 (1.000) | |  | |  | |  |
| Vif 25 | | P|small:0.035 (1.000) | |  | |  | |  |
| Vif 26 | | P|small:0.044 (1.000) | |  | |  | |  |
| Vif 27 | | P|small:0.044 (1.000) | |  | |  | |  |
| Vif 30 | | V|z5:0.014 (1.000) | |  | |  | |  |
| Vpr 1 | | V|z4:0.041 (1.000) | |  | |  | |  |
| Vpr 2 | | V|z4:0.046 (1.000) | |  | |  | |  |
| Vpu 22 | | V|hydrophobic:0.001 (0.034) | |  | |  | |  |
| Vpu 23 | | V|hydrophobic:0.002 (0.035) | |  | |  | |  |
| Vpu 24 | | V|hydrophobic:0.001 (0.034) | |  | |  | |  |
| Vpu 25 | | V|hydrophobic:<0.001 (0.021) | |  | |  | |  |
| Vpu 26 | | V|hydrophobic:0.004 (0.059) | |  | |  | |  |
| Vpu 27 | | V|hydrophobic:0.004 (0.059) | |  | |  | |  |
| Vpu 29 | | V|hydrophobic:0.023 (0.230) | |  | |  | |  |
| Vpu 30 | | P|z2:0.048 (1.000) | | V|hydrophobic:0.018 (0.209) | |  | |  |
| Vpu 31 | | P|small:0.033 (0.554) | |  | |  | |  |
| Vpu 34 | | P|small:0.035 (0.554) | |  | |  | |  |
| Vpu 35 | | P|small:0.039 (0.554) | |  | |  | |  |
| Vpu 36 | | P|small:0.048 (0.554) | |  | |  | |  |
| Vpu 37 | | P|small:0.048 (0.554) | |  | |  | |  |
| Vpu 38 | | P|small:0.003 (0.232) | |  | |  | |  |
| Vpu 39 | | P|small:0.033 (0.554) | |  | |  | |  |

^1^HXB2 Numbering

^2^Direction of effect: the physicochemical property is enriched in the Placebo (Grp = P) or the Vaccine (Grp = V) group

^3^One of the ten (Taylor ) physicochemical properties or five “z-scales” that was found to be significantly associated with treatment group at the 9-mer beginning at the site
