# Supplementary material for: Comprehensive Sieve Analysis of Breakthrough HIV-1 Sequences in the RV144 Vaccine Efficacy Trial
Source: PLoS Comput Biol. 2015 Feb 3;11(2):e1003973. doi: 10.1371/journal.pcbi.1003973 (PMC4315437; doi:10.1371/journal.pcbi.1003973)
Supplement: S13 Table — dN/dS by Physico-chemical Property (PCP) site scanning results. (DOC) [file pcbi.1003973.s022.doc]

**Table S13. dN/dS by Physico-chemical Property (PCP) site scanning results.**

| **Position^1^** | **Grp^2^|property^3^:p-value (q-value)** | | | | |
| --- | --- | --- | --- | --- | --- |
| Env 4 | V|chemical.composition:<0.001 (0.003) | P|iso-electric.point:0.009 (0.540) | V|polarity:0.006 (0.381) | P|volume:<0.001 (0.018) |  |
| Env 5 | P|chemical.composition:0.001 (0.080) | V|polarity:<0.001 (0.060) |  |  |  |
| Env 5c | V|chemical.composition:0.017 (0.798) |  |  |  |  |
| Env 6 | P|volume:<0.001 (0.018) |  |  |  |  |
| Env 27 | V|hydropathy:0.018 (0.767) |  |  |  |  |
| Env 62 | V|chemical.composition:0.023 (0.828) | P|iso-electric.point:0.024 (0.683) |  |  |  |
| Env 63 | V|iso-electric.point:0.003 (0.242) |  |  |  |  |
| Env 85 | P|chemical.composition:0.001 (0.080) | V|polarity:0.001 (0.071) |  |  |  |
| Env 86 | P|chemical.composition:0.011 (0.594) |  |  |  |  |
| Env 87 | V|iso-electric.point:0.014 (0.568) | P|volume:0.017 (0.637) |  |  |  |
| Env 153 | P|iso-electric.point:0.046 (0.964) |  |  |  |  |
| Env 165 | P|chemical.composition:0.040 (1.000) |  |  |  |  |
| Env 190 | V|hydropathy:0.038 (1.000) | P|polarity:0.007 (0.421) |  |  |  |
| Env 192 | P|chemical.composition:0.032 (0.926) |  |  |  |  |
| Env 208 | V|volume:0.008 (0.376) |  |  |  |  |
| Env 293 | V|hydropathy:<0.001 (0.005) | P|polarity:<0.001 (0.006) | V|volume:0.009 (0.376) |  |  |
| Env 308 | P|hydropathy:0.009 (0.523) | V|volume:0.008 (0.376) |  |  |  |
| Env 316 | P|chemical.composition:0.005 (0.374) |  |  |  |  |
| Env 320 | V|volume:0.016 (0.631) |  |  |  |  |
| Env 321a | P|iso-electric.point:0.012 (0.568) |  |  |  |  |
| Env 323 | P|iso-electric.point:0.036 (0.802) |  |  |  |  |
| Env 326 | V|chemical.composition:0.026 (0.828) | P|iso-electric.point:0.020 (0.603) |  |  |  |
| Env 327 | P|chemical.composition:0.047 (1.000) |  |  |  |  |
| Env 330 | V|hydropathy:0.013 (0.685) |  |  |  |  |
| Env 335 | V|polarity:0.028 (0.943) |  |  |  |  |
| Env 340 | V|iso-electric.point:0.018 (0.568) |  |  |  |  |
| Env 341 | P|chemical.composition:0.022 (0.828) | V|hydropathy:0.014 (0.685) | P|iso-electric.point:0.018 (0.568) | P|polarity:0.008 (0.421) | V|volume:0.019 (0.658) |
| Env 346 | P|polarity:0.048 (1.000) |  |  |  |  |
| Env 353 | P|chemical.composition:0.026 (0.828) | V|hydropathy:0.006 (0.390) | P|iso-electric.point:0.018 (0.568) |  |  |
| Env 358 | V|iso-electric.point:<0.001 (0.006) |  |  |  |  |
| Env 362 | P|hydropathy:0.025 (0.913) |  |  |  |  |
| Env 365 | V|chemical.composition:0.023 (0.828) | V|hydropathy:0.021 (0.819) | P|iso-electric.point:0.013 (0.568) | P|polarity:0.013 (0.637) | V|volume:0.003 (0.207) |
| Env 372 | P|iso-electric.point:0.030 (0.785) |  |  |  |  |
| Env 373 | V|polarity:0.002 (0.225) |  |  |  |  |
| Env 388 | P|hydropathy:<0.001 (<0.001) | V|iso-electric.point:0.016 (0.568) |  |  |  |
| Env 389 | P|hydropathy:0.002 (0.205) | V|polarity:0.017 (0.661) |  |  |  |
| Env 393 | V|iso-electric.point:0.031 (0.785) |  |  |  |  |
| Env 394 | P|volume:0.001 (0.064) |  |  |  |  |
| Env 419 | V|chemical.composition:0.005 (0.374) |  |  |  |  |
| Env 425 | V|chemical.composition:<0.001 (0.070) | V|hydropathy:<0.001 (0.006) | P|iso-electric.point:<0.001 (0.037) | P|polarity:<0.001 (<0.001) | V|volume:<0.001 (0.018) |
| Env 440 | V|iso-electric.point:0.002 (0.227) |  |  |  |  |
| Env 442 | V|iso-electric.point:0.003 (0.242) |  |  |  |  |
| Env 444 | P|polarity:0.015 (0.649) |  |  |  |  |
| Env 456 | V|hydropathy:<0.001 (<0.001) | V|iso-electric.point:<0.001 (<0.001) | P|polarity:0.023 (0.857) | V|volume:<0.001 (<0.001) |  |
| Env 462 | V|iso-electric.point:0.004 (0.257) |  |  |  |  |
| Env 464 | V|iso-electric.point:0.037 (0.802) |  |  |  |  |
| Env 465 | P|volume:<0.001 (0.031) |  |  |  |  |
| Env 467 | P|iso-electric.point:0.037 (0.802) |  |  |  |  |
| Env 474 | P|volume:0.050 (1.000) |  |  |  |  |
| Env 475 | V|hydropathy:0.005 (0.390) |  |  |  |  |
| Env 502 | V|chemical.composition:0.007 (0.400) |  |  |  |  |
| Env 565 | P|chemical.composition:<0.001 (0.064) | P|iso-electric.point:0.031 (0.823) | V|polarity:0.005 (0.254) | P|volume:0.023 (0.858) |  |
| Env 607 | P|volume:0.024 (0.858) |  |  |  |  |
| Env 612 | V|polarity:0.023 (0.771) |  |  |  |  |
| Env 613 | P|hydropathy:<0.001 (0.024) |  |  |  |  |
| Env 619 | V|hydropathy:<0.001 (0.040) |  |  |  |  |
| Env 621 | P|hydropathy:<0.001 (0.002) | V|polarity:0.001 (0.127) |  |  |  |
| Env 624 | V|iso-electric.point:0.004 (0.282) |  |  |  |  |
| Env 629 | P|iso-electric.point:0.013 (0.640) |  |  |  |  |
| Env 636 | P|chemical.composition:0.049 (1.000) |  |  |  |  |
| Env 641 | P|iso-electric.point:0.013 (0.640) |  |  |  |  |
| Env 644 | P|iso-electric.point:0.018 (0.640) |  |  |  |  |
| Env 645 | P|chemical.composition:0.037 (1.000) |  |  |  |  |
| Env 651 | P|hydropathy:0.011 (0.548) | V|polarity:<0.001 (0.045) | P|volume:0.002 (0.141) |  |  |
| Env 667 | P|polarity:0.043 (1.000) |  |  |  |  |
| Env 671 | P|chemical.composition:0.008 (0.485) |  |  |  |  |
| Env 674 | P|chemical.composition:<0.001 (0.001) |  |  |  |  |
| Env 676 | P|hydropathy:0.006 (0.341) |  |  |  |  |
| Env 677 | P|polarity:0.002 (0.141) |  |  |  |  |
| Env 699 | P|chemical.composition:0.033 (0.926) |  |  |  |  |
| Env 720 | P|chemical.composition:0.004 (0.378) |  |  |  |  |
| Env 736 | V|polarity:0.002 (0.160) |  |  |  |  |
| Env 750 | P|hydropathy:0.040 (1.000) |  |  |  |  |
| Env 754 | V|iso-electric.point:0.002 (0.251) |  |  |  |  |
| Env 756 | P|chemical.composition:0.002 (0.172) | V|volume:0.002 (0.139) |  |  |  |
| Env 767 | V|hydropathy:<0.001 (0.019) | P|volume:<0.001 (0.031) |  |  |  |
| Env 784 | P|volume:0.032 (1.000) |  |  |  |  |
| Env 786 | V|iso-electric.point:0.019 (0.662) |  |  |  |  |
| Env 787a | P|iso-electric.point:0.031 (0.823) |  |  |  |  |
| Env 787b | P|iso-electric.point:0.045 (1.000) |  |  |  |  |
| Env 801 | P|hydropathy:<0.001 (0.005) |  |  |  |  |
| Env 804 | P|iso-electric.point:0.024 (0.742) |  |  |  |  |
| Env 817 | P|polarity:0.001 (0.127) | V|volume:0.044 (1.000) |  |  |  |
| Env 821 | P|polarity:0.013 (0.554) | V|volume:0.003 (0.183) |  |  |  |
| Env 832 | P|hydropathy:0.024 (0.891) | V|polarity:0.004 (0.213) |  |  |  |
| Env 835 | P|iso-electric.point:0.004 (0.282) | V|volume:0.013 (0.596) |  |  |  |
| Env 836 | V|polarity:0.019 (0.665) | V|volume:0.002 (0.139) |  |  |  |
| Env 856 | P|hydropathy:0.001 (0.075) | P|iso-electric.point:<0.001 (0.032) | V|polarity:<0.001 (<0.001) |  |  |

^1^HXB2 Numbering

^2^Direction of effect: the physicochemical property is enriched in the Placebo (Grp = P) or the Vaccine (Grp = V) group

^3^One of the ten (Taylor ) physicochemical properties or five “z-scales” that was found to be significantly associated with treatment group at the 9-mer beginning at the site
