# Supplementary material for: Comprehensive Sieve Analysis of Breakthrough HIV-1 Sequences in the RV144 Vaccine Efficacy Trial
Source: PLoS Comput Biol. 2015 Feb 3;11(2):e1003973. doi: 10.1371/journal.pcbi.1003973 (PMC4315437; doi:10.1371/journal.pcbi.1003973)
Supplement: S14 Table — Summary of analyses of predicted T cell epitope sieve effects in vaccine proteins. (DOC) [file pcbi.1003973.s023.doc]

Table S14. Summary of analyses of predicted T cell epitope sieve effects in vaccine proteins.

| **Protein** | **Ref** | **MHC Class** | **Predictor^1^** | **Method^2^** | **Unadjusted p-value** | **Significance^3^** |
| --- | --- | --- | --- | --- | --- | --- |
| Gag | LAI | I | NMP | EDS | 0.998 |  |
| Gag | LAI | I | NMP | EDW | 0.92 |  |
| Gag | LAI | I | ADT | BS | 0.857 |  |
| Gag | LAI | I | ADT | PEM | 0.605 |  |
| Gag | LAI | I | NMP | PEM | 0.604 |  |
| Gag | LAI | II | NMP | EDS | 0.04 | * |
| Gag | LAI | II | NMP | EDW | 0.662 |  |
| Gag | LAI | II | NMP | BS | 0.832 |  |
| Pro | LAI | I | NMP | EDS | 0.361 |  |
| Pro | LAI | I | NMP | EDW | 0.493 |  |
| Pro | LAI | I | ADT | BS | 0.889 |  |
| Pro | LAI | I | ADT | PEM | 0.123 |  |
| Pro | LAI | I | NMP | PEM | 0.359 |  |
| Pro | LAI | II | NMP | EDS | 0.732 |  |
| Pro | LAI | II | NMP | EDW | 0.645 |  |
| Env | 92TH | I | NMP | EDS | 0.859 |  |
| Env | 92TH | I | NMP | EDW | 0.08 |  |
| Env | 92TH | I | ADT | BS | 0.627 |  |
| Env | 92TH | I | ADT | PEM | 0.332 |  |
| Env | 92TH | I | NMP | PEM | 0.603 |  |
| Env | A244 | I | NMP | EDS | 0.403 |  |
| Env | A244 | I | NMP | EDW | 0.942 |  |
| Env | A244 | I | ADT | BS | 0.031 | (*) |
| Env | A244 | I | ADT | PEM | 0.245 |  |
| Env | A244 | I | NMP | PEM | 0.542 |  |
| Env | MN | I | NMP | EDS | 0.356 |  |
| Env | MN | I | NMP | EDW | 0.605 |  |
| Env | MN | I | ADT | BS | 0.61 |  |
| Env | MN | I | ADT | PEM | 0.522 |  |
| Env | MN | I | NMP | PEM | 0.099 |  |
| Env | 92TH | II | NMP | EDS | 0.195 |  |
| Env | 92TH | II | NMP | EDW | 0.734 |  |
| Env | A244 | II | NMP | EDS | 0.108 |  |
| Env | A244 | II | NMP | EDW | 0.52 |  |
| Env | MN | II | NMP | EDS | 0.12 |  |
| Env | MN | II | NMP | EDW | 0.475 |  |

^1^Indicates whether the result is based on epitope binding predictions using the ADT predictor or the NetMHCpan (or NetMHCIIpan) predictor.

^2^Methods are EDS: EpitopeDistance using the strong binding threshold; EDW: EpitopeDistance using the weak binding threshold; PEM: PercentEpitopeMismatch; IS: EscapeCount Indel Scan; BS: EscapeCount Binding Scan; IBS: EscapeCount Indel+Binding Scan.

^3^Indicates whether the putative sieve effect had an unadjusted p-value less than 0.05. Parentheses indicate results that are “vMismatch” (showing greater evidence of T cell epitope escape among placebo recipient sequences than among vaccine recipient sequences).
