# Supplementary material for: Comprehensive Sieve Analysis of Breakthrough HIV-1 Sequences in the RV144 Vaccine Efficacy Trial
Source: PLoS Comput Biol. 2015 Feb 3;11(2):e1003973. doi: 10.1371/journal.pcbi.1003973 (PMC4315437; doi:10.1371/journal.pcbi.1003973)
Supplement: S15 Table — Summary of analyses of predicted T cell epitope sieve effects in non-vaccine proteins. (DOC) [file pcbi.1003973.s024.doc]

Table S15. Summary of analyses of predicted T cell epitope sieve effects in non-vaccine proteins.

| **Protein** | **Ref** | **MHC Class** | **Predictor^1^** | **Method^2^** | **Unadjusted p-value** | **Significance^3^** |
| --- | --- | --- | --- | --- | --- | --- |
| Gag | A244 | I | NMP | EDS | 0.351 |  |
| Gag | A244 | I | NMP | EDW | 0.705 |  |
| Gag | A244 | II | NMP | EDS | 0.806 |  |
| Gag | A244 | II | NMP | EDW | 0.064 |  |
| Nef | A244 | I | NMP | EDS | 0.803 |  |
| Nef | A244 | I | NMP | EDW | 0.468 |  |
| Nef | CON-AE | I | ADT | BS | 0.984 |  |
| Nef | LAI | I | NMP | EDS | 0.458 |  |
| Nef | LAI | I | NMP | EDW | 0.412 |  |
| Nef | A244 | II | NMP | EDS | 0.956 |  |
| Nef | A244 | II | NMP | EDW | 0.686 |  |
| Nef | LAI | II | NMP | EDS | 0.296 |  |
| Nef | LAI | II | NMP | EDW | 0.402 |  |
| Pro | A244 | I | NMP | EDS | 0.851 |  |
| Pro | A244 | I | NMP | EDW | 0.076 |  |
| Pro | A244 | II | NMP | EDS | 0.299 |  |
| Pro | A244 | II | NMP | EDW | 0.022 | * |
| RT-In | A244 | I | NMP | EDS | 0.792 |  |
| RT-In | LAI | I | NMP | EDS | 0.616 |  |
| RT-In | LAI | I | NMP | EDW | 0.068 |  |
| RT-In | A244 | II | NMP | EDS | 0.332 |  |
| RT-In | A244 | II | NMP | EDW | 0.67 |  |
| RT-In | LAI | II | NMP | EDS | 0.65 |  |
| RT-In | LAI | II | NMP | EDW | 0.659 |  |
| Rev | CON-AE | I | ADT | BS | 0.041 | (*) |
| Tat | CON-AE | I | ADT | BS | 0.383 |  |
| Vif | CON-AE | I | ADT | BS | 0.554 |  |
| Vpr | CON-AE | I | ADT | BS | 0.395 |  |
| Vpu | CON-AE | I | ADT | BS | 0.726 |  |

^1^Indicates whether the result is based on epitope binding predictions using the ADT predictor or the NetMHCpan (or NetMHCIIpan) predictor.

^2^Methods are EDS: EpitopeDistance using the strong binding threshold; EDW: EpitopeDistance using the weak binding threshold; PEM: PercentEpitopeMismatch; IS: EscapeCount Indel Scan; BS: EscapeCount Binding Scan; IBS: EscapeCount Indel+Binding Scan.

^3^Indicates whether the putative sieve effect had an unadjusted p-value less than 0.05. Parentheses indicate results that are “vMismatch” (showing greater evidence of T cell epitope escape among placebo recipient sequences than among vaccine recipient sequences).
