# Supplementary material for: Comprehensive Sieve Analysis of Breakthrough HIV-1 Sequences in the RV144 Vaccine Efficacy Trial
Source: PLoS Comput Biol. 2015 Feb 3;11(2):e1003973. doi: 10.1371/journal.pcbi.1003973 (PMC4315437; doi:10.1371/journal.pcbi.1003973)
Supplement: S16 Table — Significant EscapeCount 9-mer and 15-mer results. (DOC) [file pcbi.1003973.s025.doc]

**Table S16. Significant EscapeCount 9-mer and 15-mer results.**

| **Position^1^** | **MHC Class** | **Ref** | **p-value** | **q-value** | **vMismatch** |
| --- | --- | --- | --- | --- | --- |
| Env 5a | I | 92TH | 0.031 | 0.477 | T |
| Env 128 | I | 92TH | 0.018 | 0.45 | T |
| Env 163 | I | MN | 0.017 | 0.809 | F |
| Env 299 | I | 92TH | 0.008 | 0.336 | F |
| Env 299 | I | A244 | 0.01 | 0.355 | F |
| Env 328 | I | 92TH | 0.036 | 0.477 | T |
| Env 328 | I | A244 | 0.036 | 0.628 | T |
| Env 335 | I | 92TH | 0.022 | 0.45 | F |
| Env 335 | I | A244 | 0.025 | 0.573 | F |
| Env 363 | I | 92TH | 0.004 | 0.336 | F |
| Env 363 | I | A244 | 0.004 | 0.269 | F |
| Env 371 | I | MN | 0.027 | 0.809 | T |
| Env 428 | I | MN | 0.04 | 0.809 | T |
| Env 445 | I | MN | 0.002 | 0.237 | F |
| Gag 60 | I | LAI | 0.023 | 0.932 | T |
| Gag 332 | I | LAI | 0.044 | 0.932 | T |
| Rev 30 | I | ConAE | 0.022 | 0.315 | F |
| Rev 31 | I | ConAE | 0.047 | 0.315 | F |
| Vif 23 | I | ConAE | 0.03 | 0.522 | T |
| Vif 47 | I | ConAE | 0.044 | 0.522 | T |
| Vif 122 | I | ConAE | 0.034 | 0.522 | F |

^1^HXB2 numbering indicating 9-mer start site.
