# Supplementary material for: Comprehensive Sieve Analysis of Breakthrough HIV-1 Sequences in the RV144 Vaccine Efficacy Trial
Source: PLoS Comput Biol. 2015 Feb 3;11(2):e1003973. doi: 10.1371/journal.pcbi.1003973 (PMC4315437; doi:10.1371/journal.pcbi.1003973)
Supplement: S17 Table — Comparison of phylogenetic diversity (PD) between vaccine and placebo sequences. (DOC) [file pcbi.1003973.s026.doc]

**Table S17. Comparison of phylogenetic diversity (PD) between vaccine and placebo sequences**.

| **Tree** | **Ref** | **p-value^1^** |
| --- | --- | --- |
| Gag | LAI | 0.089^2^ |
| gp120 minus45 | 92TH | 0.69 |
| gp120 minus45 | CM244 | 0.70 |
| gp120 minus45 | MN | 0.79 |
| gp41 non-prime | CM244 | 0.38 |
| Nef | CM244 | 0.48 |
| Pro | LAI | 0.14 |
| RT+Integrase | CM244 | 0.97 |

^1^p-values are determined by a permutation procedure as described in Methods.

^2^The trend in Gag is “vMatch” (greater diversity in vaccine-recipient sub-tree than in placebo-recipient sub-tree)
