# Supplementary material for: Comprehensive Sieve Analysis of Breakthrough HIV-1 Sequences in the RV144 Vaccine Efficacy Trial
Source: PLoS Comput Biol. 2015 Feb 3;11(2):e1003973. doi: 10.1371/journal.pcbi.1003973 (PMC4315437; doi:10.1371/journal.pcbi.1003973)
Supplement: S18 Table — Comparison of phylogenetic divergence between vaccine and placebo sequences. (DOC) [file pcbi.1003973.s027.doc]

**Table S18. Comparison of phylogenetic divergence between vaccine and placebo sequences.**

| **Tree** | **Ref** | **AA *mindist* p-value^1^** | **AA all-seqs p-value** | **nt all-seqs p-value** |
| --- | --- | --- | --- | --- |
| Gag | LAI | 0.22 | 0.28 | 0.072**^3^** |
| gp120 minus45 | 92TH | 0.70 | 0.80 | 0.67 |
| gp120 minus45 | A244 | 0.70 | 0.80 | 0.70 |
| gp120 minus45 | MN | 0.51 | 0.12 | 0.84 |
| gp41 non-prime | A244 | 0.52 | 0.61 | 0.98 |
| Nef | A244 | 0.76 | 0.62 | 0.99 |
| Pro | LAI | 0.059**^2^** | 0.065**^2^** | 0.65 |
| RT+Integrase | A244 | 0.11 | 0.10 | 0.56 |

^1^p-values are determined by Wilcoxon rank-sum test as described in Methods.

^2^The trend in Pro is “vMismatch” (greater divergence among placebo recipients than among vaccine recipients)

^2^The trend in Gag (nt all-seqs) is “vMatch” (greater divergence among vaccine recipients than among placebo recipients)
