# Supplementary material for: Comprehensive Sieve Analysis of Breakthrough HIV-1 Sequences in the RV144 Vaccine Efficacy Trial
Source: PLoS Comput Biol. 2015 Feb 3;11(2):e1003973. doi: 10.1371/journal.pcbi.1003973 (PMC4315437; doi:10.1371/journal.pcbi.1003973)
Supplement: S1 Text — Sequence data construction and processing. (DOCX) [file pcbi.1003973.s028.docx]

# Text S1: Sequence data construction and processing

## Plasma specimens

HIV-1 testing was conducted at day 0, weeks 24 and 26, and every 6 months thereafter during the 3-year follow-up phase through 42 months. Specimens were screened with an immunoassay and HIV-1 infection was confirmed with a Western blot and two confirmatory HIV nucleic acid tests: the Amplicor HIV Monitor (version 1.5) assay (Roche) in Thailand and the Procleix HIV discriminatory assay (Novartis) in the United States. Earlier samples from HIV seroconverters were also tested to measure more precisely the timing of HIV-1 infection.

## HIV-1 near full-length genome (NFLG) sequencing

Plasma specimens collected at the time of HIV-1 diagnosis were obtained from 124 study volunteers. Viral RNA extracted from plasma (using the QIAamp Viral RNAKit, Qiagen, Valencia, CA) was the template for cDNA synthesis. cDNA synthesis was done using ThermoScript™ RT (Invitrogen Corp., Carisbad, CA) and HIV-specific primers at the Military HIV Research Program (MHRP), or SuperScript III Reverse Transcriptase (Invitrogen) and oligo-dT at the University of Washington (UW). NFLG or half genomes were amplified by nested PCR following endpoint-dilution of cDNA templates using the sets of primers detailed in Rolland and Edlefsen et al. (2012) [[1](#_ENREF_1)]. At MHRP, the PCR protocol followed the protocol described previously [[2](#_ENREF_2)]. At UW, a first round NFLG PCR is done with the Advantage LA Polymerase (50 $\mu$l-reaction), followed by a real-Time PCR (TaqMan® Gene Expression Master Mix) on 5 $\mu$l of the first round product for the detection of HIV-1 gag (186 bp) and env (232 bp). Two $\mu$l of the first round products identified as positive by real-time PCR are subjected to a second-round amplification using KAPA LR HS DNA Polymerase. A re-amplification using 1 $\mu$l of the first round product (dilution factor: 1 to 100) with second-round PCR primers is performed to obtain sufficient material for NFLG sequencing. PCR products were gel purified and directly sequenced with 48 primers [[3](#_ENREF_3),[4](#_ENREF_4)]. Sequencing primers were presented previously [[1](#_ENREF_1)]. Sequences have been deposited in GenBank under accession numbers JX446645–JX448316.

## Sequence alignment and translation

NFLG nucleotide sequences were error-corrected in Sequencher (Gene Codes Corporation, Ann Arbor, Michigan). All sequences from a given volunteer were then aligned manually using the Mesquite program [Maddison, W. P. and D.R. Maddison. 2011. Mesquite: a modular system for evolutionary analysis. Version 2.75 http://mesquiteproject.org] and each mutation rechecked in Sequencher. Sequences with ambiguous base calls were accepted if the contig showed up to two ambiguous calls for half genomes or five ambiguous calls for NFLG. Each intra-host alignment was screened for phylogenetically-informative sites, which were identified by removing all private mutations (mutations occurring only once) from the set of sequences using the InSites algorithm (http://indra.mullins.microbiol.washington.edu/DIVEIN/insites.html). The amplification strategy was to obtain 5 to 10 NFLG genome sequences per specimen, depending on intra-host sequence variation assessments: if 5 or more phylogenetically-informative sites were found in the first 5 NFLG sequences generated per individual, then approximately 5 additional NFLG sequences were amplified and sequenced. Coding sequences were extracted from alignments using Gene Cutter (http://www.hiv.lanl.gov), and multi-subject alignments were created using MUSCLE [[5](#_ENREF_5)], as implemented in Seaview [[6](#_ENREF_6)]. Codon-based alignments of individual genes along with the translated protein sequence output were manually refined as needed in Mesquite.

## Vaccine sequences

Immunizations consisted of four priming injections of a recombinant canarypox vector vaccine (ALVAC-HIV [vCP1521]) and two booster injections of a recombinant env gp 120 subunit vaccine (AIDSVAX B/E). The vaccine sequences corresponded to lab isolates of HIV-1 subtype B and HIV-1 subtype E strains isolated in Thailand in 1990 and 1992. The ALVAC-HIV [vCP1521] is a chimeric construct that concatenates *gag* and *pro* of HIV-1 subtype B (strain LAI) with gp120 of HIV-1 subtype E (strain 92TH023) fused to a 28-AA-long segment of the transmembrane-anchoring portion of gp41 HIV-1-B strain LAI (HXB2 position AA 684:711 of HXB2 gp160). AIDSVAX B/E is composed of two gp120 proteins that are truncated in 5’ (HIV-1 protein start at AA42 of HXB2 gp160): one protein is HIV-1 subtype B (strain MN) and one is HIV-1 subtype E (strain CM244).

## Definition of the *mindist* sequence: genome-level selection process

In our previous sieve analysis [[1](#_ENREF_1)] the *mindist* sequence set used for analysis contained one observed Env sequence from each subject, with the selected sequence having the shortest Hamming distance to the consensus sequence of that subject’s observed sequences. Ties were broken first through selection of the sequence with fewer unknown amino acids (“X”), and then through random selection.

With this comprehensive analysis we used a new selection process that was more resistant to ties, and was employed at the whole- or (when necessary) partial-genome level, to reduce the possibility of selecting genes from multiple unique sequences, which would result in a chimeric recombinant construct. This process incorporated all full-length (FL), right-half (RH) and left-half (LH) sequences that were observed from each subject, and resulted in one selected sequence for each protein from each subject.

The new *mindist* selection process was as follows:

- For each subject:
  - The whole-genome consensus sequence was determined from all the subject's sequences.
  - For each observed sequence:
    - The distance between the sequence and the consensus was calculated using the Tamura-Nei ‘93 (TN93) distance correction model.
    - Gaps were regarded as missing data, so were not included in the calculation.
  - The following sequences were selected, where present:
    - The whole-genome sequence(s) with the shortest distance (deemed *x*).
    - The LH sequence(s) with the shortest distance (deemed *y*).
    - The RH sequence(s) with the shortest distance (deemed *z*).
    - If any significant deletions were present in either *x*, *y*, or *z*, we chose the appropriate sequence with the next-shortest distance that did not contain such deletions.
    - If sequences *y* and *z* did not overlap, then selected the LH or RH sequence with the next-shortest distance that facilitated an overlap.
  - If multiple sequences contended for selection with *x*, *y*, or *z* because of tied distances, ties were broken with the following sequential criteria:
    - The sequences with the most ambiguous, incomplete or stop codons were excluded.
    - For RH genomes, the sequence(s) with the shortest env distance was selected.
    - For LH genomes, the sequence(s) with the shortest gag distance was selected.
    - If any ties remained after the above, they were broken through random selection.
    - At the end of this step, at most one sequence was selected for each of *x*, *y*, and *z*.
  - Of sequences *x*, *y*, and *z*:
    - If there were at least four whole-genome sequences for this subject and *x*'s distances were comparable to those of any present LH or RH sequences, then *x* was chosen as the mindist sequence.
      - Sequences for all proteins were translated from *x*.
    - If there were three or fewer whole-genome sequences or if *x*'s distance was not comparable to the distances of *y* or *z*, then *y* and *z* were chosen as the mindist sequences, and protein-level sequences were selected from *y* and *z*, depending on which side of the genome they were on.
      - Gag, Pol, Vif, and Vpr come from the left half.
      - Tat, Rev, Vpu, Env, and Nef come from the right half.

In our process of *mindist* selection for these observed sequences, five ties remained after the selection procedure and needed to be broken randomly.

The differences between the *mindist* set selected for this analysis and that was selected for the original V1V2 analysis was minimal. There were no amino acid differences at the V2 sites discovered to be significant in the V1V2 analysis (Env 169 and Env 181). Additionally, there were no circumstances where a tie-breaking random selection would have resulted in a change at these two sites.

TN93 was shown to have the highest resolution (resulting in the fewest ties) among the different methods that were evaluated (hamming distance, Felsenstein ’81, Kimura ’80, TN93), although the distances calculated between the different methods were highly correlated (Pearson correlation > 0.97, p < 0.001).
