# Supplementary material for: Comprehensive Sieve Analysis of Breakthrough HIV-1 Sequences in the RV144 Vaccine Efficacy Trial
Source: PLoS Comput Biol. 2015 Feb 3;11(2):e1003973. doi: 10.1371/journal.pcbi.1003973 (PMC4315437; doi:10.1371/journal.pcbi.1003973)
Supplement: S2 Text — The SmoothMarks method. (DOCX) [file pcbi.1003973.s029.docx]

Text S2: The SmoothMarks Method

As discussed in the main text, the SmoothMarks analysis did not find significant evidence for a decline in vaccine efficacy with genetic distance from the vaccine immunogen based on the ten specific site sets studied, as shown in Figure S2. Here we provide complete results and a more detailed description of the method.

For each HIV-1 sequence from a subject, the distance $V$ was computed as the sum of values V′ calculated at each included amino acid site, $V=1-2\frac{S(X,Y)}{S(X,X)+S(Y,Y)}$, where $S$ is the HIVb scoring matrix [[7](#_ENREF_7)] alignment score between the participant’s sequence $X$ and the vaccine immunogen sequence$Y$. 109 infected subjects (42 vaccine, 64 placebo) were included in the analysis, of which 94 (37 vaccine, 57 placebo) had an observed sequence distance (those with sequences available by the HIV-1 infection diagnosis visit). The distances were defined based on the *mindist* sequence as defined earlier. The methods of Sun and Gilbert (2012) [[8](#_ENREF_8)] and Gilbert, Self and Ashby (1998) [[9](#_ENREF_9)] were used to estimate VE(*v*) over the range of observed distances *v*, where the vaccine efficacy against HIV-1 with distance *v*, VE(*v*), is one minus the distance *v*-specific hazard ratio (vaccine/placebo) of HIV-1 infection multiplied by 100%. These methods were also used to construct 95% confidence intervals for VE(*v*) over the range of distances *v*, as well as to test two null hypotheses of interest: first, that VE(*v*) = 0% for all *v* (no vaccine efficacy at all against any HIV-1 genotype), and, secondly, that VE(*v*) = VE for all *v* (vaccine efficacy is the same against all HIV-1 genotypes, i.e., there is no sieve effect).

The method of Sun and Gilbert uses a model to predict whether $V$ is observed among HIV-1 infected subjects, and also uses a model to predict the missing values of $V$, and the method provides consistent/unbiased estimation even if one of these models is incorrect. Ninety-eight of the 109 subjects (89%) had sequences measured at or before the antibody-based diagnosis date (43 vaccine, 66 placebo) and 11 afterwards (39 vaccine, 59 placebo), at 15−217 days after this date, median 30 days after. The sequences of the remaining eleven subjects were measured from a post-diagnosis sample and hence for these subjects *V* was missing. To predict the probability of observing $V$ among the 109 infected subjects, we used all-subsets logistic regression model selection considering demographics, host genetics, and biomarker data post-infection such as plasma viral load. The best model by the Bayesian Information Criterion included only the years from entry until HIV infection diagnosis, $X_{1}$, with model fit estimated logit[P(R=1|infected,X_1_)] = $1.17+0.70X_{1}$. In addition, we studied linear and logistic regression models for relating the mean of various potential auxiliary variables measured in all HIV infected subjects to $V$,X_1_, and treatment indicator $Z$. Model selection did not reveal any significantly predictive auxiliary variables; we expect that HIV-1 sequence information measured after the acute phase would be a good predictor, but these data were not collected. Nevertheless, we select the best available auxiliary predictor variable, gender (X_2_, 1=male; 0=female), and use the resulting fitted logistic regression model in the analysis. The results were nearly identical with and without the auxiliary variable; therefore this factor did not affect the results, and we present only the results that exclude the auxiliary variable. The estimation and testing procedures of Gilbert and Sun (2014) [[10](#_ENREF_10)] were applied with bandwidths $h_{1}=0.5$ and $h_{2}=h=0.3$, and tuning parameters parameters $a=0.05$ for both references 92TH023 and CM244, $b=1$ and $aʹ=1+0.01$. In particular, the $T_{m1}^{(2)}$ test statistic of Gilbert and Sun (2014) was used.
