# Supplementary material for: Comprehensive Sieve Analysis of Breakthrough HIV-1 Sequences in the RV144 Vaccine Efficacy Trial
Source: PLoS Comput Biol. 2015 Feb 3;11(2):e1003973. doi: 10.1371/journal.pcbi.1003973 (PMC4315437; doi:10.1371/journal.pcbi.1003973)
Supplement: S3 Text — HLA-dependent covariation analysis. (DOCX) [file pcbi.1003973.s030.docx]

# Text S3: HLA-dependent Covariation Analysis

### Phylogenetic dependency networks

We used a phylogenetic comparative method, which also explicitly models the evolutionary history of the sequences, to identify covarying interactions between residues [[11](#_ENREF_11)]. We used phylogenetic dependency networks, a statistical model of evolution that simultaneously takes into account HIV-1 AA co-variation, linkage disequilibrium among HLA alleles, and shared ancestry in the HIV-1 phylogeny to identify the primary source of selection pressure acting on each HIV codon [[12](#_ENREF_12)]. For each gene, a maximum likelihood phylogenetic tree was constructed and a model of conditional adaptation was created for the vaccine status and for every HLA gene, amino-acid position and state. The high computational cost of this Bayesian graphical model was reduced by removing from the analysis highly conserved sites at which nonsynonymous substitutions occurred in fewer than 1% of the branches in the tree. The null hypothesis is that the observations depend on the phylogenetic tree structure; then, adaptation due to each variable is modeled along the tree by an additive process. All results were adjusted for multiple comparisons, using Benjamini-Hochberg q-values of ≤ 0.2 as the significance threshold (implying a maximum expected false-positive proportion of 20% among identified associations [[13](#_ENREF_13)]).

We used phylogenetic dependency networks to identify associations among residues and associations to the type I and type II HLA genotypes or the vaccine status. Based on the tree topology, all variables were tested to evaluate whether they were associated with the character state at each branch tip. In this analysis, we included all sequences from each individual. Covariation was treated as independent among sequences (conditional on the phylogeny); in contrast, HLA escape and vaccine-induced effects were modeled using a latent variable that linked selection pressure across all sequences in an individual. For example, when testing for HLA-mediated escape from HLA *h* at position *i*, we included a latent variable *h_ij_* for each subject *j*. If this latent variable was active, then all sequences would be subject to the same selection pressure, and would thus escape with the same probability *α*. If this latent variable was not active, then none of the sequences would be subject to selection pressure. *h_ij_* was active with probability 0 if the individual did not express the HLA allele *h*, and was active with probability *β* if the individual did express *h*. The likelihood of the resulting model was maximized with respect to *α* and *β,* in addition to the phylogenetic parameters.

In Env, we identified 26 covarying interactions between particular AAs at pairs of sites when only the vaccine status was used as a predictor, and a total of 51 associations when the HLA genotypes were also included in the analysis. In addition, we found six HLA-associated sites among the 817 sites in the Env alignment. All six associations were identified with a q-value under 0.2. These results are shown in Dataset S2.

#### V2 is a Hub

Given the role of the gp70-V1V2 reagent in the correlate of risk studies, we assessed the proportion of associations that involved residues from gp70-V1V2: 24.1% (14/58) of the residues in V1/V2 were involved in AA-to-AA interactions, while only 6.7% of residues were involved in associations in the remainder of Env; hence, a significantly higher proportion of gp120-V1V2 residues were found to be co-varying when compared to the rest of Env (two-tailed Fisher’s exact test p < 0.0001). Likewise, V2 residues were significantly more likely (p < 0.0001) to be found as co-varying (11/31 = 35.5%) than residues outside of V2. This result, reported previously [[1](#_ENREF_1)], illustrates that V2 appears as a hub of interactions in the covariation network that we identified.

Among the six HLA-driven associations in the Env alignment, the strongest was found in V2 at Env 165 [with isoleucine (I) as the adapted residue] which was linked to HLA-DRB1*11 with p-value = 1.66E-11, q-value = 7.01E-07. DRB1*11 has been associated with non-responsiveness to ALVAC vCP1521 + AIDSVAX B/E vaccination [[14](#_ENREF_14)]. We also noted HLA-associations at site Env 65, with the presence of HLA-DRB1*07 associated with valine (V) escaping (p = 6.88E-08, q = 0.001), and at Env 95 with carriage of HLA-A*68 associated with the presence of methionine (M) (p = 3.86E-06, q = 0.16).

We found no significant association between pairs of V1/V2 residues. Two residues from V1/V2 (Env 165 and Env 181) were associated with other residues in Env (outside of V1/V2). There was an association between Env 181 and Env 65 (q=0.12) and there were ten associations between Env 165 and other Env residues – four of which showed particularly strong q-values (<6E-06): isoleucine (I) at position 165 was associated with Env 356 (G escaping) at q = 2.10E-07, with Env 507 (D escaping) at q = 5.73E-06, with Env 715 (not L) at q = 7.17E-08, and with Env 842 (not H) at q = 2.42E-09.

The other V2 residue found to be co-varying was site 181: T@181 was negatively associated with V@65 (i.e., one residue was escaping in the presence of the other) (q = 0.12). Interestingly, an analysis restricted to testing the vaccine effect identified two sites that were associated with vaccine status: positions Env 65 and 721, both showing vaccine efficacy associated with escape of V@65 and T@721 (p = 6.34E-05, q = 0.11; and p = 0.0002, q = 0.16, respectively). Importantly, these associations were no longer significant when other variables (namely, HLA class I and II alleles for each individual) were added to the model.

Since we identified a vaccine effect linked to position 65 of the envelope, we wanted to assess CTL epitope predictions corresponding to that region. We focused on epitopes starting at positions 51 (TLFCASDAK, HLA-A*03:01,A*03:02,A*11:01,A*11:02,A*11:04,A*68:01,A*74:01), 61 (H/QETEV/AHNVW; HLA-B*18:01,B*44:03), 63 (TEV/AHNVWAT; HLA-B*18:01,B*40:06,B*41:01,B*50:01), 64 (EVHNVWATH; HLA-A*34:01,A*68:01), 67 (NVWATHACV; HLA-A*02:01,A*02:05,A*02:06,A*02:11,A*02:16). There was no significant difference between treatment arms; for example, if we considered the comparison that showed the most significant p-value (CM244), the mean distance in breakthrough sequences was 0.008 in vaccine recipients (n = 32) and 0.021 in placebo recipients (n = 40) (p = 0.16).
