# Supplementary material for: Comprehensive Sieve Analysis of Breakthrough HIV-1 Sequences in the RV144 Vaccine Efficacy Trial
Source: PLoS Comput Biol. 2015 Feb 3;11(2):e1003973. doi: 10.1371/journal.pcbi.1003973 (PMC4315437; doi:10.1371/journal.pcbi.1003973)
Supplement: S4 Text — The EscapeCount method. (DOCX) [file pcbi.1003973.s031.docx]

### Text S4: The EscapeCount Method

We define three distances that measure differences in amino acid sequence from the perspective of CD4+ and CD8+ T cell epitopes. Each distance models a distinct mode by which changes in the amino-acid sequence relative to a vaccine immunogen sequence reduce the potency of T cell epitopes and allow the virus to “escape”. Specifically we consider immune escape by (1) point-mutations that reduce HLA binding affinity of an epitope, and (2) insertions or deletions that abrogate epitope binding. In each case, we compare the sequence of each vaccine sequence to those of breakthrough infections in both vaccine and placebo recipients (using the *mindist* set of one sequence per subject). Epitopes are identified on the vaccine sequence using HLA binding prediction models for the HLA alleles of trial participants. These methods predict the binding affinity (IC50) for a given HLA-peptide combination. We consider both HLA class I and class II epitopes. HLA binders are defined as peptides whose IC50 < $\gamma_{low}$, the binding threshold. Predictions were computed using the following methods:

- HLA Class I (CD8+ epitopes) – Adaptive Double Threading [[15](#_ENREF_15)]. We consider only 9-mer peptides, the predominant size of class I binders.
- HLA class II (CD4+ epitopes) – NetMHCIIpan [[16](#_ENREF_16)]. Predictions were provided for 15-mer peptides.

With each method, we compute the IC50 of every k-mer of the vaccine amino acid sequence with the HLA alleles genotyped from each study participant. Epitopes are defined as k-mers with IC50 values below the binding threshold $\gamma_{low}$. Each of these epitopes is compared to the corresponding k-mer at the same alignment location in the participant’s breakthrough virus sequence to identify changes that may lead to immune escape. Using these models, we define the following T cell sieve distances:

1. Binding escape – For each participant and each vaccine sequence we compute the number of predicted epitopes in which the predicted IC50 of the corresponding k-mer on the participant’s breakthrough virus sequence is above the escape threshold $\gamma_{high}$.
2. Insertion/deletion escape – For each participant and vaccine sequence we compute the number of predicted epitopes on the vaccine sequence in which there is an insertion or deletion within the epitope in the corresponding breakthrough sequence.
3. Combined escape distance – The above methods that measure binding escape and insertion/deletion escape are combined by summing for each person the total number of escapes identified using each method. Since each type of escape is computed based on independent and complimentary epitope features, if all types contribute to immune escape then the combined distance should be a more sensitive metric for detecting a sieve effect.

The above methods, which rely on MHC binding predictors, all require a binding threshold parameter ($\gamma_{low}$) that defines a predicted epitope. Method 1 also requires an escape threshold ($\gamma_{high}$) that defines an escaped epitope. Canonical definitions of HLA binders include two thresholds for binding: “strong” binders – which have a IC50 < 50nM, and “weak” binders – which have a IC50 < 500nM. However, because of the many factors that contribute to the immunodominance of an epitope and the complexity of selection pressures (which are not all immune-derived), the MHC binding parameters are plausibly different for each epitope. Therefore we varied these parameter values over the ranges $4\leq\gamma_{low}\leq7$ and $4\leq\gamma_{high}\leq10$, where $\gamma_{high}-\gamma_{low}>1$, in units of log(nM). We use a maximum t-statistic permutation test to determine if there is significant evidence for a sieve effect at any parameter combination. The test finds the maximum value of the (two-sample, pooled-variance) t statistic of the difference across groups (of the number of “escapes”) over all parameter combinations in this range, and compares this maximum to a null distribution of the maximum, estimated by permutation over treatment assignment labels. The p-value is determined as the fraction of permutations for which the maximum t statistic is greater than or equal to the observed maximum t-statistic, over 50,000 random permutations.

While the three methods above each define a global sieve distance over an entire vaccine sequence, they can also be used to define local and site-specific sieve distances. Global sieve analyses, which evaluate the sum of escapes over all k-mers, determine whether or not there is a statistically significant sieve effect over an entire protein, but cannot identify which amino-acid residues are driving the effect. In contrast, k-mer-specific and site-specific sieve analyses test if there is significant evidence of a sieve effect over a particular k-mer or at a single site. Single-site analyses sum over the all k-mers that overlap the site. We restrict the site-specific analyses to sites that lie in predicted vaccine epitopes for $\geq$ 10% of participants and to sites that are diverse in the breakthrough sequences (> 3 individuals with a minority variant).

We analyzed predicted HLA epitope binding affinities for evidence of weaker predicted epitope binding in subjects’ viral sequences as compared to vaccine immunogen sequences.

For a particular set of thresholds of binding (to the immunogen sequence) and escape (reduced binding to the *mindist* sequence), we counted the number of escapes, and then compared this count across treatment groups. We also assessed the number of predicted epitopes in which gaps in the breakthrough sequences might abrogate binding. For both analyses, thresholds were varied over a range and p-values were assessed to account for these varying thresholds. Site-specific, k-mer, and global tests were performed, with global tests indicating greater binding escape in Env versus the MN sequence among vaccine recipients than among placebo recipients (p=0.048).

Global EscapeCount results are presented in Table S14 and Table S15; Significant k-mer-specific results are presented in Table S16. Complete k-mer and site-specific results are included in Dataset S1.
