# Supplementary material for: Comprehensive Sieve Analysis of Breakthrough HIV-1 Sequences in the RV144 Vaccine Efficacy Trial
Source: PLoS Comput Biol. 2015 Feb 3;11(2):e1003973. doi: 10.1371/journal.pcbi.1003973 (PMC4315437; doi:10.1371/journal.pcbi.1003973)
Supplement: S5 Text — The PercentEpitopeMismatch method. (DOCX) [file pcbi.1003973.s032.docx]

#### Text S5: The PercentEpitopeMismatch Method

The ‘percent epitope mismatch distance’ is based on predicted peptide binders in the reference sequence. Using NetMHCpan, the first step in computing the distance is to estimate the number of predicted peptide binders, based on each subject’s HLA-A and HLA-B alleles, that are shared between the reference sequence and the *mindist* sequences. A peptide is counted as shared if there is an exact match between the reference sequence and all of the *mindist* breakthrough sequences. The distance is the percent of mismatched peptides, defined as one minus the ratio of the number of shared peptides (computed in the first step) and the peptides in the reference sequence. The denominator is the sum across all 9-mers in the reference sequence that are binders using the ‘weak’ binder threshold (500 nM). The distance was also computed using the Adaptive Double Threading (ADT) [[15](#_ENREF_15)] prediction algorithm, using a threshold of 500 nM. These distances were computed for each immunogen sequence separately. Predicted peptide binders were also required to be listed among the known CTL epitopes listed on LANL. 9-mer regions with 4 or fewer subjects without a mismatch were filtered out. For each distance measure, we used a two-sided Wilcoxon rank-based Choplump test to compare distributions of the percent epitope mismatch between the infected vaccine and placebo groups [[17](#_ENREF_17)]. Results are shown in Table S14 and Table S15.
