# Supplementary material for: Comprehensive Sieve Analysis of Breakthrough HIV-1 Sequences in the RV144 Vaccine Efficacy Trial
Source: PLoS Comput Biol. 2015 Feb 3;11(2):e1003973. doi: 10.1371/journal.pcbi.1003973 (PMC4315437; doi:10.1371/journal.pcbi.1003973)
Supplement: S6 Text — The EpitopeDistance method. (DOCX) [file pcbi.1003973.s033.docx]

### Text S6: The EpitopeDistance Method

We used NetMHCpan to predict CD8+ T cell epitopes (and to identify known epitopes) in all volunteer-derived and vaccine sequences based on each individual’s HLA genotype [[18](#_ENREF_18)] (http://www.cbs.dtu.dk/services/NetMHCpan/). Likewise, NetMHCIIpan was used to predict CD4+ T cell epitopes [[16](#_ENREF_16)] (http://www.cbs.dtu.dk/services/NetMHCIIpan/). Both predictors use artificial neural networks and provide quantitative peptide MHC binding data (given in nM IC50), which are used to discern strong and weak binders. With NetMHCpan, only 9-mers were considered because nine is the favored length for binding, and predictions for other lengths are made from approximations based on 9-mers. With NetMHCIIpan, peptides were retained based on the core 8-mer peptide sequence: between several overlapping peptides with an identical core peptide, the peptide that had the strongest binding affinity was retained. For each individual, we only retained epitope predictions that were unique, meaning a unique allele and peptide combination, i.e., if a peptide is predicted by two different alleles, both peptide/allele combinations are considered.

We considered measures of distance between each of a subject’s sequences and a reference sequence. Genetic distances were calculated using the HIVb model of evolution as described above [[7](#_ENREF_7)]. Epitope distances are defined in three steps. First, potential epitopes were identified as described above in subjects’ and reference sequences, based on each individual HLA genotype. Second, we focused on the subset of epitopes that overlapped (i.e., had the same HXB2 positions) between the subjects’ sequence(s) and the reference sequence (with at most 2 AA differences). Third, we computed all pairwise distances between each epitope sequence and the reference sequence using the HIVb-10 evolutionary model in HyPhy. The epitope distance was then defined for each subject as the average of the different epitope-specific pairwise distances. If there were no known or highly likely epitopes in either sequence, then the distance could not be defined and the subject’s information was not used. For each distance measure, we used a two-sided Wilcoxon to test for a different distribution in the sequence summary measures between the infected vaccine and placebo groups.

We found that the number of predicted epitopes for each individual did not significantly differ between vaccine and placebo recipients. Mean epitope distances to the CM244 immunogen were significantly greater for vaccine than for placebo recipients (0.095 vs. 0.070; p = 0.013) when epitopes identified as strong binders were considered, indicating that within probable epitopes, breakthrough sequences were more divergent from the vaccine among vaccine recipients than among placebo recipients. Epitope distances were also greater for vaccine recipients than placebo recipients when the other vaccine immunogens (92TH023, MN) were considered as the reference; however the difference between these distances was not significant. Additionally, epitope distances based on epitopes derived from other proteins (Gag, Pol) showed no significant difference between the vaccine and placebo groups. There was no significant difference when the analyses were restricted to CTL epitopes derived solely from V2 or the V1/V2 subregion constituting the HIV-1 portion of the gp70-V1V2 fusion protein used to detect the V2 binding antibody correlate of risk in RV144.
