# Supplementary material for: Comprehensive Sieve Analysis of Breakthrough HIV-1 Sequences in the RV144 Vaccine Efficacy Trial
Source: PLoS Comput Biol. 2015 Feb 3;11(2):e1003973. doi: 10.1371/journal.pcbi.1003973 (PMC4315437; doi:10.1371/journal.pcbi.1003973)
Supplement: S7 Text — Supplementary references. (DOCX) [file pcbi.1003973.s034.docx]

**Text S7: Supplementary References**

1. Rolland M, Edlefsen PT, Larsen BB, Tovanabutra S, Sanders-Buell E, et al. (2012) Increased HIV-1 vaccine efficacy against viruses with genetic signatures in Env V2. Nature 490: 417-420.

2. Rolland M, Tovanabutra S, deCamp AC, Frahm N, Gilbert PB, et al. (2011) Genetic impact of vaccination on breakthrough HIV-1 sequences from the STEP trial. Nat Med 17: 366-371.

3. Rodrigo AG, Goracke PC, Rowhanian K, Mullins JI (1997) Quantitation of target molecules from polymerase chain reaction-based limiting dilution assays. AIDS Res and Hum Retrovir 13: 737-742.

4. Frenkel LM, Wang Y, Learn GH, McKernan JL, Ellis GM, et al. (2003) Multiple viral genetic analyses detect low-level human immunodeficiency virus type 1 replication during effective highly active antiretroviral therapy. J Virol 77: 5721-5730.

5. Edgar RC (2004) MUSCLE: multiple sequence alignment with high accuracy and high throughput. Nucleic Acids Res 32: 1792-1797.

6. Galtier N, Gouy M, Gautier C (1996) SEAVIEW and PHYLO_WIN: two graphic tools for sequence alignment and molecular phylogeny. Comput Appl Biosci 12: 543-548.

7. Nickle DC, Heath L, Jensen MA, Gilbert PB, Mullins JI, et al. (2007) HIV-specific probabilistic models of protein evolution. PLoS One 2: e503.

8. Sun Y, Gilbert PB (2012) Estimation of stratified mark-specific proportional hazards models with missing marks. Scand Stat Theory Appl 39: 34-52.

9. Gilbert PB, Self SG, Ashby MA (1998) Statistical methods for assessing differential vaccine protection against human immunodeficiency virus types. Biometrics 54: 799-814.

10. Gilbert PB, Sun Y (2014) Testing for vaccine efficacy against a spectrum of pathogen sequences in stratified mark-specific proportional hazards models with missing marks, with application to the RV144 HIV vaccine efficacy trial. Journal of the Royal Statistical Society, Series C, in press.

11. Poon AF, Lewis FI, Pond SL, Frost SD (2007) An evolutionary-network model reveals stratified interactions in the V3 loop of the HIV-1 envelope. PLoS Comput Biol 3: e231.

12. Carlson JM, Brumme ZL, Rousseau CM, Brumme CJ, Matthews P, et al. (2008) Phylogenetic dependency networks: inferring patterns of CTL escape and codon covariation in HIV-1 Gag. PLoS Comput Biol 4: e1000225.

13. Benjamini Y, Hochberg Y (1995) Controlling the false discovery rate - a practical and powerful approach to multiple testing. Journal of the Royal Statistical Society Series B-Methodological 57: 289-300.

14. Paris R, Bejrachandra S, Thongcharoen P, Nitayaphan S, Pitisuttithum P, et al. (2011) HLA class II restriction of HIV-1 clade-specific neutralizing antibody responses in ethnic Thai recipients of the RV144 prime-boost vaccine combination of ALVAC-HIV and AIDSVAX((R)) B/E. Vaccine.

15. Guindon S, Gascuel O (2003) A simple, fast, and accurate algorithm to estimate large phylogenies by maximum likelihood. Syst Biol 52: 696-704.

16. Nielsen M, Justesen S, Lund O, Lundegaard C, Buus S (2010) NetMHCIIpan-2.0 - Improved pan-specific HLA-DR predictions using a novel concurrent alignment and weight optimization training procedure. Immunome Res 6: 9.

17. Follmann D, Fay MP, Proschan M (2009) Chop-lump tests for vaccine trials. Biometrics 65: 885-893.

18. Nielsen M, Lundegaard C, Blicher T, Lamberth K, Harndahl M, et al. (2007) NetMHCpan, a method for quantitative predictions of peptide binding to any HLA-A and -B locus protein of known sequence. PLoS ONE 2: e796.
